# Comparative effectiveness of non-pharmacological interventions for post-stroke upper limb motor dysfunction: a systematic review and network meta-analysis of randomized controlled trials

**DOI:** 10.3389/fnagi.2026.1852556

**Published:** 2026-07-03

**Authors:** Xuhui Yang, Yuhan Wang, Jingjing Xing, Yaning Liu, Tiechun Zhang, Lichuan Zeng, Xingyu Chen, Can Wang, Peiyan Wang, Ruizhao Lu, Jiangwei Shi, Zihan Yin, Ling Zhao

**Affiliations:** 1 Acupuncture and Tuina School, Chengdu University of Traditional Chinese Medicine, Chengdu, Sichuan, China; 2 First Teaching Hospital of Tianjin University of Traditional Chinese Medicine, Tianjin, China; 3 National Clinical Research Center for Chinese Medicine, Tianjin, China

**Keywords:** motor dysfunction, network meta-analysis, rehabilitation, stroke, upper limb

## Abstract

**Background:**

Post-stroke upper limb motor dysfunction (PS-ULMD) is a common and disabling consequence of stroke. Although multiple non-pharmacological interventions are used to treat PS-ULMD, the optimal treatment remains unclear. Thus, this study aimed to identify effective non-pharmacological interventions for improving upper limb motor function in patients with PS-ULMD.

**Methods:**

Eight databases were searched from database inception to May 18, 2026. Randomized controlled trials (RCTs) evaluating 15 non-pharmacological interventions for PS-ULMD were included based on multiple guidelines. Risk of bias was assessed using the Cochrane Risk of Bias tool (RoB 2.0). Primary outcome was the improvement in the Fugl-Meyer Assessment for Upper Extremity (FMA-UE). Pairwise meta-analyses were conducted using Review Manager (RevMan, version 5.4). Network meta-analyses were performed using STATA (version 15.0) and ADDIS (version 1.16.8). The quality of evidence for outcomes was evaluated using the Confidence in Network Meta-Analysis (CINeMA) online tool.

**Results:**

A total of 89 RCTs, involving 5129 participants, were included. The RoB 2.0 tool indicated that the majority of included studies presented some concerns. Pairwise meta-analyses showed that, compared with conventional therapy, non-pharmacological interventions significantly improved FMA-UE scores [mean difference (MD) = 4.95, 95% confidence interval (CI): 3.74–6.17]. Compared with sham control, the overall effect remained statistically significant (MD = 5.12, 95% CI: 3.43–6.81). Network meta-analysis further indicated that BCI, CIMT, BF, and PES were associated with relatively larger improvements in FMA-UE scores compared with conventional therapy and sham control. However, according to the CINeMA framework, the overall certainty of evidence for most comparisons was rated as low to very low.

**Conclusion:**

Overall, non-pharmacological interventions appear to be effective in improving post-stroke upper limb function. Among them, BCI, CIMT, BF, and PES were associated with relatively greater improvements in upper extremity motor recovery. However, the certainty of the evidence remains limited, and further high-quality RCTs are warranted to confirm these findings and to better define the relative effectiveness of non-pharmacological interventions.

**Systematic review registration:**

https://www.crd.york.ac.uk/PROSPERO/, identifier CRD420251243062.

## 1 Introduction

Stroke is the third leading cause of death and the fourth leading cause of disability worldwide, based on the Global Burden of Disease (GBD) 2021 study. Stroke frequently results in post-stroke upper limb motor dysfunction (PS-ULMD), which represents one of the most disabling and clinically significant sequelae following stroke (Ingram et al., 2021; Langhorne et al., 2009). These impairments involve multiple dimensions of motor function, including deficits in motor control and coordination, impaired fine and gross motor performance, reduced or lost muscle strength, abnormal muscle tone, and substantial limitations in activities of daily living (Lv et al., 2023; Pundik et al., 2019; Tomita et al., 2017; Yamamoto et al., 2022; Yong et al., 2022). Epidemiological studies indicate that PS-ULMD is highly prevalent among patients with stroke. During the acute phase, approximately 50–80% of patients experience upper limb dysfunction, and even in the chronic phase, 55–75% of patients continue to present with upper limb dysfunction (Lawrence et al., 2001; Nakayama et al., 1994; Persson et al., 2012). Impairment of upper limb motor function markedly reduces patients’ independence in daily life and increases their reliance on family members, while also predisposing them to emotional disorders such as depression and anxiety; these emotional problems further hinder the recovery of motor function (Assadi Khalil et al., 2024; Li et al., 2023). Therefore, recovery of PS-ULMD represents a key clinical issue for improving overall functional outcomes.

Currently, therapeutic strategies for PS-ULMD mainly include pharmacological and non-pharmacological interventions. Pharmacological treatments, such as levodopa and fluoxetine, have been investigated. However, their therapeutic effects are often modest and inconsistent, and some studies have reported potential adverse effects (Engelter et al., 2025; Hesse and Werner, 2003). Consequently, current clinical practice guidelines for stroke tend to favor non-pharmacological interventions, including constraint induced movement therapy (CIMT), repetitive facilitation exercise (RFE), task oriented training (TOT), upper limb training (ULT), brain computer interface (BCI), peripheral electrical stimulation (PES), transcranial direct current stimulation (tDCS), vagus nerve stimulation (VNS), transcranial magnetic stimulation (TMS), mirror therapy (MT), virtual reality (VR), biofeedback (BF), robot-assisted training (RAT), acupuncture (Acup), and motor imagery training (MIT). Accumulating evidence indicates that non-pharmacological therapies are effective in improving upper limb motor function in stroke survivors and are generally considered safe, with no obvious adverse effects reported (Bolognini et al., 2020; Dawson et al., 2021; Zhang et al., 2025). However, most of this evidence relies on comparisons with conventional treatment or sham stimulation, and direct head-to-head comparisons between non-pharmacological interventions remain scarce. This gap in comparative evidence prevents a clear understanding of their relative efficacy and limits the development of evidence-based treatment hierarchies. Therefore, establishing the optimal intervention is essential to advance the field and inform clinical practice.

Network meta-analysis (NMA) allows the integration of direct and indirect evidence within a unified analytical framework, enabling simultaneous comparison and ranking of multiple interventions (Faltinsen et al., 2018; Salanti, 2012; Tonin et al., 2017), thereby providing a means to identify the optimal therapy. Therefore, this study employed a Bayesian network meta-analysis to compare and rank various non-pharmacological therapies for PS-ULMD, aiming to provide robust evidence to support the clinical application of non-pharmacological interventions and to guide the selection of optimal treatment strategies.

## 2 Materials and methods

The study design follows the PRISMA-NMA guideline (Hutton et al., 2015; Supplementary Table 1) and has been registered with PROSPERO (Registration No. CRD420251243062).

### 2.1 Inclusion criteria and exclusion criteria

#### 2.1.1 Types of studies

All randomized controlled trials (RCTs) published in English or Chinese were included, without restrictions on region or publication source. Excluded were animal studies, non-randomized clinical studies, quasi-randomized trials, cluster-randomized trials, case reports, and studies from which data could not be extracted.

#### 2.1.2 Types of participants

Participants were aged ≥ 18 years, diagnosed with stroke according to established clinical criteria, and exhibited clear PS-ULMD. Patients with upper limb motor impairments secondary to other comorbidities or unrelated diseases were excluded. Studies involving patients with ischemic or hemorrhagic stroke, different stroke stages (acute, subacute, or chronic) (Bernhardt et al., 2017), first-ever or recurrent stroke, and varying severities of upper limb motor impairment (Ierardi et al., 2026) were eligible for inclusion.

#### 2.1.3 Types of interventions

The following four clinical practice guidelines were reviewed: *Global Stroke Guidelines and Action Plan: A Road Map for Quality Stroke Care* (Lindsay et al., 2019), *Guidelines for Adult Stroke Rehabilitation and Recovery: A Guideline for Healthcare Professionals From the American Heart Association/American Stroke Association* (Winstein et al., 2016), *European Stroke Organisation (ESO) guideline on motor rehabilitation* (Alt Murphy et al., 2025), *and Stroke rehabilitation in adults: Clinical Guideline [NG236]* (National Institute for Health and Care Excellence, 2023). The interventions included: CIMT, RFE, TOT, ULT, BCI, PES, tDCS, VNS, TMS, MT, VR, BF, RAT, Acup, and MIT. Detailed descriptions of these interventions are presented in Supplementary Table 2. Each intervention, alone or combined with conventional therapy, was included as an independent node in the network, while combinations with other non-pharmacological interventions were excluded.

#### 2.1.4 Types of control groups

The control groups included conventional therapy (CON) and sham stimulation (sham).

#### 2.1.5 Types of outcome measures

Studies reporting one or more of the predefined outcomes were included: the primary outcome was the change in Fugl-Meyer Assessment for Upper Extremity (FMA-UE), while the secondary outcomes included the change in Activities of Daily Living (ADL), which was specifically measured using the Modified Barthel Index (MBI), Action Research Arm Test (ARAT), Wolf Motor Function Test (WMFT), Box and Block Test (BBT), overall Grip Strength (GS), and Modified Ashworth Scale (MAS).

### 2.2 Search strategy

To ensure a comprehensive literature search, this study systematically searched eight databases, including PubMed, Web of Science, Embase, Cochrane Library, China National Knowledge Infrastructure (CNKI), VIP Database, Wanfang Database, and SinoMed Database (SinoMed). The search period spanned from database inception to May 18, 2026, and included studies published in both Chinese and English. To ensure effective retrieval, a combination of Medical Subject Headings (MeSH) and free-text terms was employed. Search terms were combined using the Boolean operators “AND” and “OR.” The search strategies were tailored to each database, and the detailed strategies are provided in Supplementary Appendix 1.

### 2.3 Study selection and data extraction

Two researchers independently conducted study screening and data extraction; first, all search results from each database were imported into EndNote (version X21), and duplicates across databases were removed. Second, irrelevant studies were preliminarily excluded by reviewing titles and abstracts. Finally, full texts were assessed to further exclude studies unrelated to this research. Subsequently, the researchers cross-checked their screening results; studies were included when the assessments were consistent, and in cases of disagreement, a third researcher was consulted, with final inclusion determined through discussion and consensus.

For eligible trials, two trained researchers independently extracted data from the included studies using a standardized data extraction form and summarized the risk of bias. Extracted data primarily included: (1) basic information of the included studies (title, year of publication, first author, country); (2) participants’ demographic characteristics (age, sex, stroke type, hemiplegic side, handedness, stroke phase, recurrence status, time since stroke); (3) intervention details (type, duration, and frequency of intervention); and (4) we extracted specific data for each outcome measure (FMA-UE, ADL, ARAT, WMFT, BBT, GS, MAS), including means and standard deviations, and calculated MD to facilitate comparison. The data extracted by the two researchers were cross-checked, and discrepancies were resolved through discussion or adjudication by a third researcher. When necessary, the authors of the studies were contacted to obtain missing information.

### 2.4 Risk of bias assessment

The risk of bias for all RCTs was independently assessed. This process was conducted using the RoB 2.0 tool by two independent reviewers, covering five key domains: the randomization process, deviations from intended interventions, missing outcome data, outcome measurement, and selection of reported results. After the independent assessments, the two reviewers cross-checked their evaluations. In cases of disagreement, a third reviewer was consulted, and consensus was reached through discussion and adjudication.

### 2.5. Statistical methods

#### 2.5.1 Pairwise meta-analysis

This study conducted conventional pairwise meta-analyses of the included studies using Review Manager 5.4. The included RCTs used baseline-to-endpoint change scores as outcome measures. For multi-arm trials, each was split into multiple pairwise comparisons, with the control group sample size evenly divided according to recommendations in the Cochrane Handbook (Axon et al., 2023). Continuous data were expressed as mean differences (MD) with 95% confidence intervals (CI). Heterogeneity among studies was assessed using the I^2^ statistic and the Cochran’s Q test. Given potential clinical and methodological differences between studies, all data were pooled using a random-effects model.

#### 2.5.2 Network meta-analysis

To compare the effects of different non-pharmacological interventions, a Bayesian network analysis was performed using the Aggregate Data Drug Information System (ADDIS, version 1.16.8; Drugis, Groningen, the Netherlands), with the Markov Chain Monte Carlo (MCMC) method (Cipriani et al., 2013). For multi-arm trials, the correlation between effect estimates from the same study was accounted for by the software’s built-in multi-arm correction. Continuous outcomes were reported as MD with 95% CI. A random-effects model was adopted to account for potential between-study heterogeneity. Non-informative priors provided by ADDIS were applied to all model parameters. Model fit was assessed using the deviance information criterion (DIC), with lower values indicating a better balance between model fit and complexity. The parameters were set at 4 chains for simulation, while the simulation iterations were set to 50,000. First, we performed 20,000 adjustment iterations to eliminate the effect of the initial value, then, integrated indirect and direct evidence from all the RCTs according to the node splitting method. Meanwhile, STATA software Version.15.0 (Stata Corp. LP, College Station, TX, United States) was used to generate plots of the network meta-analysis and compare each outcome. Finally, we generated ranking probability plots for all interventions, after which local inconsistency was assessed using the node-splitting method. Generally, all nodes showed *P* > 0.05 in inconsistency tests, implying that no significant statistical difference existed between direct and indirect comparisons. Model convergence was assessed using the potential scale reduction factor (PSRF), with values close to 1 indicating adequate convergence.

### 2.6 Subgroup analysis

Given the clinical heterogeneity across stroke rehabilitation trials, subgroup analyses were conducted for FMA-UE based on stroke stage (acute, subacute, and chronic) and severity of upper-limb motor impairment (mild, moderate, and severe) to explore potential sources of heterogeneity and assess the robustness of the findings.

### 2.7 Sensitivity analysis

Sensitivity analyses were conducted for FMA-UE to assess the robustness of the findings. These analyses included: (1) restriction to sham-controlled trials; (2) exclusion of studies with high risk of bias; (3) exclusion of studies involving recurrent stroke or mixed/unclear stroke populations when data were available; and (4) stratification of interventions into adjunctive (add-on) and stand-alone rehabilitation interventions.

### 2.8 Publication bias

To assess potential publication bias in the included studies, funnel plots were used for qualitative evaluation in conventional pairwise meta-analyses. For network meta-analyses, adjusted funnel plots were generated for each outcome measure to qualitatively evaluate the potential for small-study effects and publication bias.

### 2.9 Certainty of evidence assessment

The certainty of evidence for the NMA estimates was assessed using the Confidence in Network Meta-Analysis (CINeMA) framework (Nikolakopoulou et al., 2020; Papakonstantinou et al., 2020), which applies the GRADE approach to network meta-analyses. CINeMA accounts for the integration of direct and indirect evidence and uses the contribution matrix to quantify the influence of each direct comparison on the network estimates, facilitating the assessment of within-study bias and indirectness. Evidence certainty was evaluated across six domains: within-study bias, reporting bias, indirectness, imprecision, heterogeneity, and incoherence. Each domain was rated as no concerns, some concerns, or major concerns, and an overall certainty level (high, moderate, low, or very low) was assigned for each treatment comparison and outcome. The certainty of evidence was independently assessed by two reviewers. Any disagreements were resolved through discussion and, when necessary, consultation with a third reviewer. The same reviewers and consensus process used in study selection and data extraction were also applied in this process.

## 3 Results

### 3.1 Search results

**FIGURE 1:**
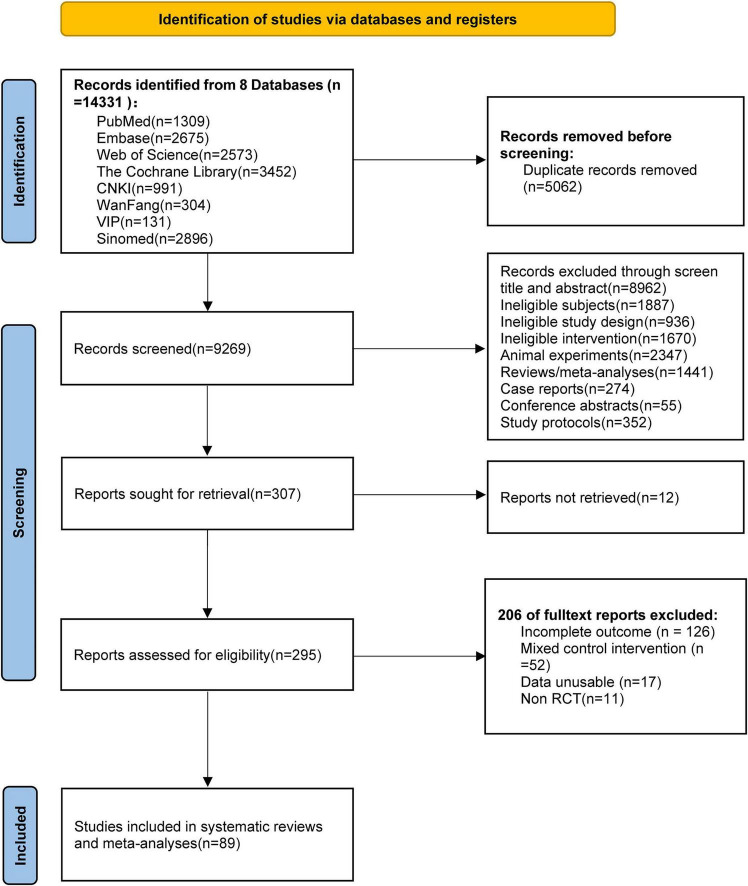
The PRISMA flow chart of selection process.

The literature screening process is illustrated in Figure 1. A total of 14,331 potentially eligible articles were retrieved. After removing 5,062 duplicates, 9,269 articles remained for further screening. After preliminary screening of titles and abstracts, 8,962 articles were excluded, leaving 307 full-text articles for detailed review. Of these, 218 articles were further excluded due to unavailable full texts, incomplete outcome data, mixed interventions, unavailable data, or non-randomized study designs, resulting in 89 studies being included (Adie et al., 2017; Ali et al., 2024; Allman et al., 2016; Ambrosini et al., 2021; Anwar et al., 2022; Aprile et al., 2020; Au-Yeung and Hui-Chan, 2014; Bhattacharjee et al., 2024; Budhota et al., 2021; Chen et al., 2023, 2024; Choi et al., 2014; Dawson et al., 2021; de Sire et al., 2025; Dehem et al., 2019; Dou et al., 2019; Du et al., 2016; Fu et al., 2023; Gurbuz et al., 2016; Han et al., 2016; He et al., 2025; Hou et al., 2019, 2026; Hsieh et al., 2018; Hsu et al., 2022; Ji et al., 2025; Jiang et al., 2022; Kaviraja et al., 2021; Kim et al., 2015, 2016, 2023; Kimberley et al., 2018; Knutson et al., 2026; Kuo et al., 2023; Lee and Chun, 2014; Liang et al., 2020; Lim et al., 2016; Lin et al., 2009, 2015; Liu et al., 2023; Long et al., 2018; Meng et al., 2017; Ming et al., 2025; Morone et al., 2026; NETS Trial Collaboration Group, 2024; Oh et al., 2019; Ohnishi et al., 2022; Park, 2022; Pavan et al., 2024; Prange et al., 2015; Rémy-Néris et al., 2021; Sale et al., 2014; Schuster-Amft et al., 2018; Seniów et al., 2012; Seok et al., 2016; Sharma et al., 2020; Shimodozono et al., 2013; Shin et al., 2016; Singh and Pradhan, 2013; Sun et al., 2022, 2024, 2026; Thrane et al., 2015; Timmermans et al., 2014; Tomić et al., 2017; Vimolratana et al., 2024; Wang et al., 2020, 2022, 2023, 2026, Wei et al., 2022; Wongwatcharanon et al., 2025; Wu et al., 2007, 2013; Xia et al., 2021; Xiao et al., 2019; Yang et al., 2020, 2023; Yin et al., 2015; Peng et al., 2015; Zhang et al., 2016, 2017, 2020, 2021, 2023, 2024, 2025; Zhao et al., 2015; Zhuang et al., 2021). The search cutoff date for these studies was May 18, 2026.

### 3.2 Characteristics of included studies

A total of 89 RCTs were included, of these, 26 studies were published in Chinese and 63 in English, and the trials were conducted across 19 countries (e.g., China, Korea, India, and the United Kingdom). The included studies were published between 2007 and 2026. Interventions evaluated included CIMT, RFE, TOT, ULT, BCI, PES, tDCS, VNS, TMS, MT, VR, BF, RAT, Acup, and MIT. Detailed descriptions of the interventions are provided in Supplementary Table 2, while stimulation parameters for electrophysical and neuromodulation interventions (PES, TMS, tDCS, and VNS) are summarized in Supplementary Table 3. Most RCTs adopted a 1:1 allocation ratio, with treatment durations ranging from 2 to 24 weeks. The included studies involved patients across different stroke stages, including acute (5 studies), subacute (55 studies), chronic phases (22 studies), 1 study enrolled patients across mixed stroke stages, and 6 studies did not report stroke stage. Regarding upper limb motor impairment severity, 1 study included patients with mild impairment, 52 studies included patients with moderate impairment, 23 studies included patients with severe impairment, 1 study enrolled patients with mixed impairment severity, and 12 studies did not report severity. First-ever stroke patients were included in 65 studies, recurrent stroke patients in 2 studies, and recurrence status was not reported in 22 studies. Eleven studies exclusively included patients with ischemic stroke, whereas most studies did not distinguish between ischemic and hemorrhagic stroke. Handedness was reported in 15 studies, and participants were predominantly right-handed. For outcome assessment, the FMA-UE was reported in 81 studies, ADL in 25 studies, ARAT in 21 studies, WMFT in 15 studies, BBT in 15 studies, GS in 8 studies, and MAS in 5 studies. Detailed characteristics of the included studies are presented in Supplementary Table 4.

### 3.3 Risk of bias assessment

**FIGURE 2:**
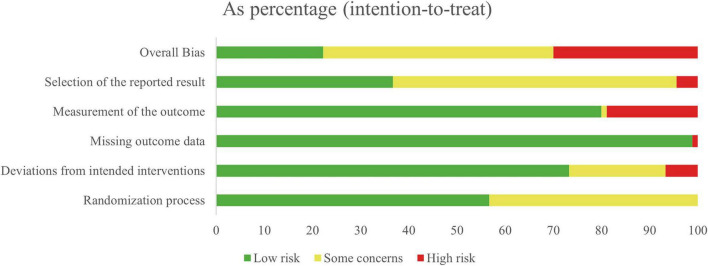
Risk of bias graph.

Among the 89 included studies, risk of bias assessment indicated that 19 studies were at low risk, 43 studies at some risk, and 27 studies at high risk. Regarding specific domains, 84 studies reported randomization methods, such as random number tables, online randomization systems, or drawing lots; 51 studies implemented allocation concealment; 29 studies reported blinding; 70 studies clearly blinded outcome assessors; and 32 studies had a low risk of bias in selective reporting. The risk of bias summary for the included studies is shown in Figure 2, while detailed risk of bias assessments are presented in Supplementary Table 5.

### 3.4 Pairwise meta-analysis

#### 3.4.1 Primary outcome

##### 3.4.1.1 Improvement in FMA-UE

We conducted 10 pairwise meta-analyses using CON as the comparator to evaluate the effectiveness of different non-pharmacological interventions in improving FMA-UE, including CIMT, RFE, ULT, PES, MT, VR, BF, RAT, Acup, and MIT. Detailed results are presented in Supplementary Figure 1A. The pooled analysis demonstrated that these non-pharmacological interventions were overall effective in improving FMA-UE (MD = 4.95, 95%CI: 3.74–6.17). Specifically, statistically significant improvements were observed for RFE (MD = 5.34, 95% CI: 4.58–6.09), ULT (MD = 7.89, 95% CI: 4.37–11.41), BF (MD = 7.62, 95% CI: 1.93–13.30), RAT (MD = 3.23, 95% CI: 1.40–5.07), and MIT (MD = 3.80, 95% CI: 1.31–6.29) compared with CON. In addition, the improvements observed with ULT and BF exceeded the minimal clinically important difference (MCID) threshold of 6.0 (Tenberg et al., 2023), suggesting potentially meaningful clinical benefits. Overall heterogeneity across the CON-controlled comparisons was substantial (*I*^2^ = 84%). Substantial heterogeneity was observed in comparisons involving CIMT (*I*^2^ = 90%) and VR (*I*^2^ = 87%), while moderate heterogeneity was found for RAT (*I*^2^ = 62%), low heterogeneity was identified for RFE (*I*^2^ = 0%) and BF (*I*^2^ = 8%).

In addition, we conducted 8 pairwise meta-analyses using sham stimulation as the comparator, involving BCI, PES, tDCS, VNS, TMS, MT, Acup, and MIT. Detailed results are presented in Supplementary Figure 1B. The pooled analysis also demonstrated an overall beneficial effect of these non-pharmacological interventions on FMA-UE (MD = 5.12, 95%CI: 3.43–6.81). Specifically, statistically significant improvements were observed for PES (MD = 8.45, 95%CI: 1.04–15.85), VNS (MD = 2.46, 95%CI: 1.97–2.95), TMS (MD = 5.45, 95%CI: 3.90–6.99), MT (MD = 5.48, 95%CI: 0.71–10.26), and Acup (MD = 7.50, 95%CI: 1.18–13.82) compared with sham. Notably, the effect sizes of PES and Acup exceeded the MCID threshold, suggesting potentially meaningful clinical benefits. Overall heterogeneity across the sham-controlled comparisons was substantial (*I*^2^ = 87%). Substantial heterogeneity was observed in comparisons involving BCI (*I*^2^ = 95%) and PES (*I*^2^ = 95%), while moderate heterogeneity was found for MIT (*I*^2^ = 63%). Low heterogeneity was identified for tDCS (*I*^2^ = 17%), VNS (*I*^2^ = 0%), and TMS (*I*^2^ = 18%), whereas low to moderate heterogeneity was observed for MT (*I*^2^ = 27%).

#### 3.4.2 Secondary outcomes

##### 3.4.2.1 Improvement in ADL

We conducted three pairwise meta-analyses using CON as the comparator to evaluate the effectiveness of different non-pharmacological interventions in improving ADL, including VR, BF, and RAT. Detailed results are presented in Supplementary Figure 2A. The pooled analysis demonstrated that these non-pharmacological interventions were overall effective in improving ADL (MD = 6.72, 95% CI: 5.39–8.05). Specifically, a statistically significant improvement was observed for RAT (MD = 6.80, 95% CI: 5.15–8.46) compared with CON. Overall heterogeneity across the CON-controlled comparisons was low (*I*^2^ = 6%). Low-to-moderate heterogeneity was observed for VR (*I*^2^ = 30%) and RAT (*I*^2^ = 16%).

In addition, we conducted six pairwise meta-analyses using sham stimulation as the comparator, involving BCI, PES, tDCS, TMS, MT, and MIT. Detailed results are presented in Supplementary Figure 2B. The pooled analysis demonstrated that these non-pharmacological interventions were effective in improving ADL (MD = 9.72, 95% CI: 8.65–10.79). Specifically, statistically significant improvements were observed for BCI (MD = 11.35, 95% CI: 9.48–13.22), PES (MD = 8.96, 95% CI: 7.31–10.62), TMS (MD = 4.70, 95% CI: 1.04–8.37), and MIT (MD = 12.38, 95% CI: 9.40–15.35) compared with sham. Overall heterogeneity across sham-controlled comparisons was moderate (*I*^2^ = 69%). High heterogeneity was observed in PES (*I*^2^ = 89%), whereas no heterogeneity was detected for TMS and MIT (*I*^2^ = 0%).

##### 3.4.2.2 Improvement in ARAT

We conducted three pairwise meta-analyses using CON as the comparator to evaluate the effectiveness of different non-pharmacological interventions in improving ARAT, involving RFE, ULT, and RAT. Detailed results are presented in Supplementary Figure 3A. The pooled analysis demonstrated that these non-pharmacological interventions were effective in improving ARAT (MD = 3.95, 95% CI: 0.81–7.10). Specifically, statistically significant improvements were observed for RFE (MD = 6.52, 95% CI: 5.66–7.38) and ULT (MD = 2.30, 95% CI: 0.98–3.62) compared with CON. Overall heterogeneity across the CON-controlled comparisons was substantial (*I*^2^ = 88%). Moderate heterogeneity was observed for RAT (*I*^2^ = 36%).

In addition, we conducted 2 pairwise meta-analyses using sham stimulation as the comparator, involving PES and tDCS. Detailed results are presented in Supplementary Figure 3B. The pooled analysis demonstrated that these non-pharmacological interventions were effective in improving ARAT (MD = 4.85, 95% CI: 2.78–6.92). Specifically, a statistically significant improvement was observed for tDCS (MD = 4.82, 95% CI: 0.19–9.45) compared with sham stimulation. Overall heterogeneity across sham-controlled comparisons was low (*I*^2^ = 0%). Low heterogeneity was observed for tDCS (*I*^2^ = 0%), whereas moderate heterogeneity was observed for PES (*I*^2^ = 54%).

##### 3.4.2.3 Improvement in WMFT

We conducted 4 pairwise meta-analyses using CON as the comparator to evaluate the effectiveness of different non-pharmacological interventions in improving WMFT, involving CIMT, PES, RAT, and BF. Detailed results are presented in Supplementary Figure 4A. The pooled analysis demonstrated that these non-pharmacological interventions were effective in improving WMFT performance (MD = 5.99, 95% CI: 1.80–10.18). Specifically, statistically significant improvements were observed for PES (MD = 12.60, 95% CI: 8.58–16.62) and RAT (MD = 5.04, 95% CI: 2.32–7.76) compared with CON. Overall heterogeneity across the CON-controlled comparisons was substantial (*I*^2^ = 91%). Very high heterogeneity was observed in CIMT (*I*^2^ = 97%), whereas no heterogeneity was detected for RAT and BF (*I*^2^ = 0%).

In addition, we conducted three pairwise meta-analyses using sham stimulation as the comparator, involving tDCS, VNS, and TMS. Detailed results are presented in Supplementary Figure 4B. The pooled analysis demonstrated that these non-pharmacological interventions were effective in improving WMFT performance (MD = 1.98, 95% CI: 0.50–3.46). Specifically, a statistically significant improvement was observed for TMS (MD = 9.35, 95% CI: 5.26–13.44) compared with sham stimulation. Overall heterogeneity across sham-controlled comparisons was substantial (*I*^2^ = 95%). Low heterogeneity was observed for tDCS (*I*^2^ = 8%), whereas very high heterogeneity was observed for VNS (*I*^2^ = 98%).

##### 3.4.2.4 Improvement in BBT

We conducted three pairwise meta-analyses using CON as the comparator to evaluate the effectiveness of different non-pharmacological interventions in improving BBT, involving MT, VR, and RAT. Detailed results are presented in Supplementary Figure 5A. The pooled analysis indicated that these non-pharmacological interventions were not significantly effective in improving BBT (MD = 0.93, 95% CI: −1.86 to 3.72). Specifically, no statistically significant improvements were observed for MT, VR, or RAT compared with CON. Overall heterogeneity across the CON-controlled comparisons was low (*I*^2^ = 0%). Low heterogeneity was also observed for VR and RAT (both *I*^2^ = 0%).

In addition, only 1 RCT was included in the comparison between sham stimulation and the intervention for BBT. Detailed results are presented in Supplementary Figure 5B. The results showed that the intervention was not superior to sham stimulation (MD = −0.50, 95% CI: −12.59 to 11.59). Heterogeneity could not be assessed due to the inclusion of a single study.

##### 3.4.2.5 Improvement in GS

We conducted three pairwise meta-analyses using CON as the comparator to evaluate the effectiveness of different non-pharmacological interventions in improving GS, involving VR, BF, and RAT. Detailed results are presented in Supplementary Figure 6A. The pooled analysis indicated that these non-pharmacological interventions were not significantly effective in improving GS (MD = 0.31, 95% CI: −1.12 to 1.74). Specifically, no statistically significant improvements were observed for VR, BF, or RAT compared with CON. Overall heterogeneity across the CON-controlled comparisons was low (*I*^2^ = 0%). Low heterogeneity was also observed for VR (*I*^2^ = 0%).

In addition, two pairwise meta-analyses were conducted using sham stimulation as the comparator, involving PES and TMS. Detailed results are presented in Supplementary Figure 6B. The pooled analysis demonstrated that these non-pharmacological interventions were not significantly effective in improving GS (MD = 2.62, 95% CI: −1.09 to 6.33). Specifically, a statistically significant improvement was observed for TMS (MD = 4.10, 95% CI: 2.60–5.60) compared with sham stimulation. Overall heterogeneity across sham-controlled comparisons was moderate (*I*^2^ = 68%). Heterogeneity could not be assessed for the single-study comparison.

##### 3.4.2.6 Reduction in MAS

We conducted one pairwise meta-analyses using CON as the comparator to evaluate the effectiveness of RAT in reducing MAS. The results showed that RAT was not effective in reducing MAS compared with CON (MD = 1.00, 95% CI: −1.28 to 3.28). Detailed results are presented in Supplementary Figure 7A.

In addition, only 1 RCT was included in the comparison between tDCS and sham stimulation. The results showed that tDCS was not effective in reducing MAS compared with sham stimulation (MD = −0.30, 95% CI: −1.01 to 0.41). Heterogeneity could not be assessed due to the inclusion of a single study in each comparison. Detailed results are presented in Supplementary Figure 7B.

### 3.5 Network meta-analysis

#### 3.5.1 Network plot for different interventions

**FIGURE 3:**
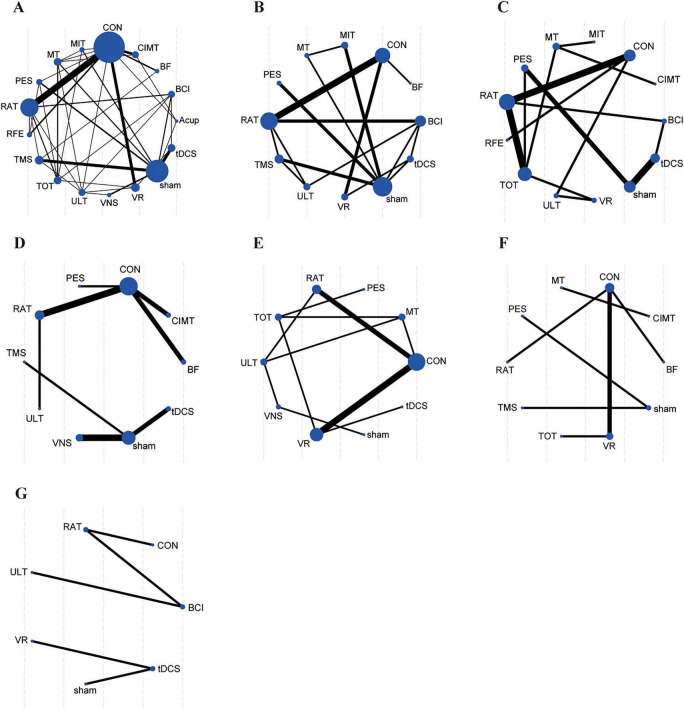
Network plots of outcomes. **(A)** Network plots of FMA-UE. **(B)** Network plots of ADL. **(C)** Network plots of ARAT. **(D)** Network plots of WMFT. **(E)** Network plots of BBT. **(F)** Network plots of GS. **(G)** Network plots of MAS.

We conducted an NMA to evaluate the effects of various interventions on FMA-UE, ADL, ARAT, WMFT, BBT, GS, and MAS in patients with PS-ULMD, aiming to identify optimal rehabilitation strategies for different aspects of upper limb motor function. Figure 3 illustrates the network plots for the different outcome measures, where the size of each node represents the sample size of the corresponding intervention, and the thickness of the lines between nodes reflects the number of direct comparison studies.

#### 3.5.2 Primary outcome

##### 3.5.2.1 Improvement in FMA-UE

**FIGURE 4:**
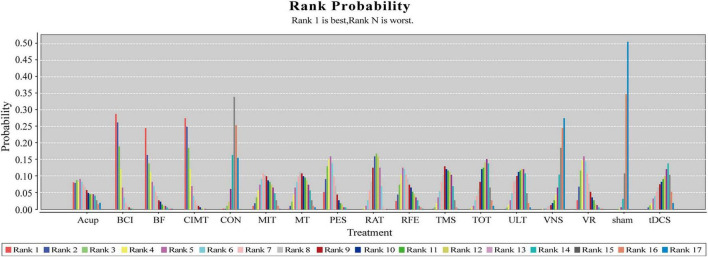
The figure of ranking probability of improvement in FMA-UE.

**FIGURE 5:**
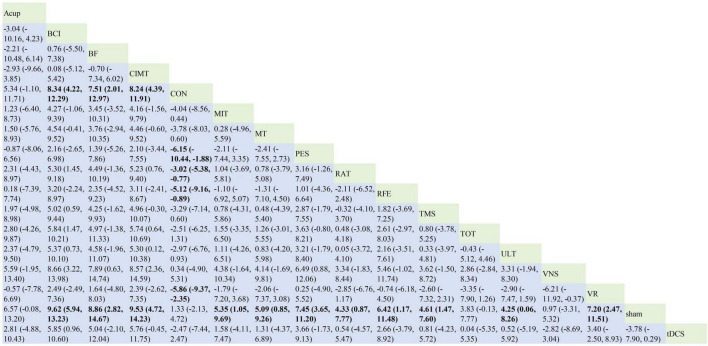
Network meta-analysis league table for FMA-UE. The results are reported based on the lower triangular matrix of the ADDIS league table. Therefore, effect estimates are expressed as column-defining treatments versus row-defining treatments (column vs. row). Bold values indicate statistically significant differences.

Network plots for the 15 interventions were generated using STATA 15.0 (Figure 3A). Based on the PSRF values approaching 1 and the corresponding *p* values (Supplementary Table 6), a consistency model was therefore applied for the network meta-analysis. Subsequently, ranking probability plots (Figure 4) and ranking probability tables (Table 1) were produced. Compared with CON, BCI, BF, CIMT, PES, RAT, RFE, and VR showed statistically significant improvements in FMA-UE. Among these interventions, BCI (MD = 8.34, 95% CI: 4.22–12.29), CIMT (MD = 8.24, 95% CI: 4.39–11.91), and BF (MD = 7.51, 95% CI: 2.01–12.97) demonstrated relatively larger effect sizes. Compared with sham stimulation, BCI, BF, CIMT, MIT, MT, PES, RAT, RFE, TMS, ULT, and VR demonstrated statistically significant benefits. Similarly, BCI (MD = 9.62, 95% CI: 5.94–13.23), CIMT (MD = 9.53, 95% CI: 4.72–14.23), and BF (MD = 8.86, 95% CI: 2.82–14.67) showed relatively greater improvements, as shown in Figure 5. In addition, the ranking analysis suggested that BCI, CIMT, and BF were among the top-ranked interventions for improving FMA-UE. Notably, the effect sizes of BCI, CIMT, BF and PES exceeded the reported MCID threshold for FMA-UE, suggesting potentially meaningful clinical benefits.

**TABLE 1:** Posterior probabilities of interventions ranking first across different outcome measures.

FMA-UE		ADL		ARAT		BBT	
Treatment	Rank1	Treatment	Rank1	Treatment	Rank1	Treatment	Rank1
BCI	0.29	MIT	0.39	MIT	0.29	tDCS	0.53
CIMT	0.27	BCI	0.33	BCI	0.25	PES	0.15
BF	0.24	PES	0.15	CIMT	0.16	RAT	0.15
Acup	0.08	BF	0.09	RFE	0.11	Sham	0.09
PES	0.05	MT	0.03	tDCS	0.08	MT	0.04
RFE	0.03	VR	0.01	PES	0.05	TOT	0.01
VR	0.03	CON	0	MT	0.02	ULT	0.01
MIT	0.01	RAT	0	VR	0.02	VR	0.01
CON	0	TMS	0	ULT	0.01	CON	0
MT	0	ULT	0	Sham	0.01	VNS	0
RAT	0	Sham	0	CON	0		
TMS	0	tDCS	0	RAT	0		
TOT	0			TOT	0		
ULT	0						
VNS	0						
Sham	0						
tDCS	0						

#### 3.5.3 Secondary outcomes

##### 3.5.3.1 Improvement in ADL

**FIGURE 6:**
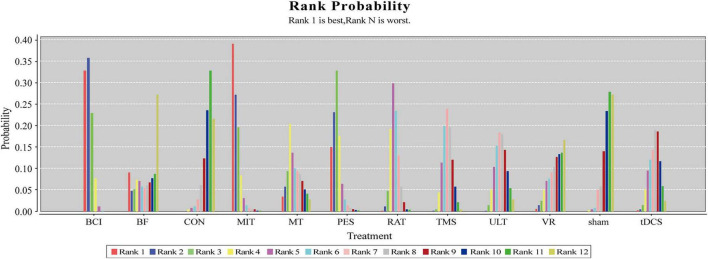
The figure of ranking probability of improvement in ADL.

**FIGURE 7:**
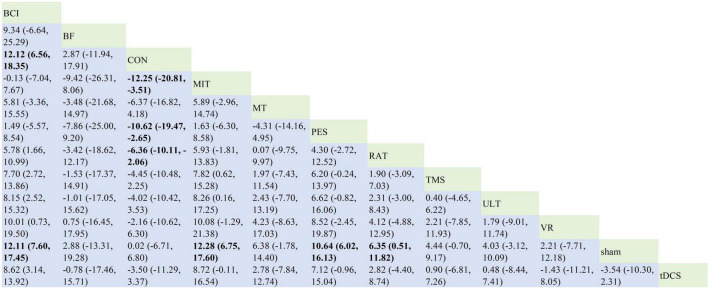
Network meta-analysis league table for ADL. The results are reported based on the lower triangular matrix of the ADDIS league table. Therefore, effect estimates are expressed as column-defining treatments versus row-defining treatments (column vs. row). Bold values indicate statistically significant differences.

Network plots for the 10 interventions were generated using STATA 15.0 (Figure 3B). Based on PSRF values approaching 1 and the corresponding *p* values (Supplementary Table 7), a consistency model was applied for the network meta-analysis. Ranking probability plots (Figure 6) and ranking probability tables (Table 1) were subsequently generated. The results showed that, compared with CON, BCI, MIT, PES, and RAT showed statistically significant improvements in ADL. Among these interventions, MIT (MD = 12.25, 95% CI: 3.51–20.81), BCI (MD = 12.12, 95% CI: 6.56–18.35), and PES (MD = 10.62, 95% CI: 2.65–19.47) demonstrated relatively larger effect sizes. Similarly, compared with sham stimulation, BCI, MIT, PES, and RAT also demonstrated statistically significant benefits. MIT (MD = 12.28, 95% CI: 6.75–17.60), BCI (MD = 12.11, 95% CI: 7.60–17.45), and PES (MD = 10.64, 95% CI: 6.02–16.13) showed relatively greater improvements, with detailed network meta-analysis results presented in Figure 7.

##### 3.5.3.2 Improvement in ARAT

**FIGURE 8:**
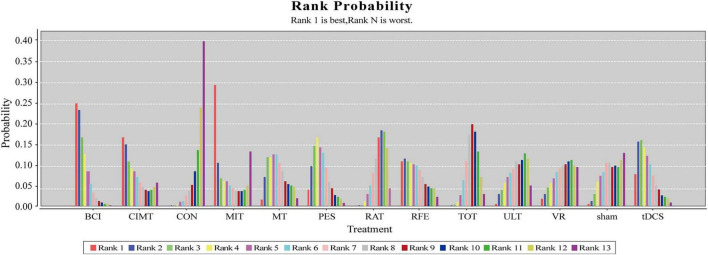
The figure of ranking probability of improvement in ARAT.

**FIGURE 9:**
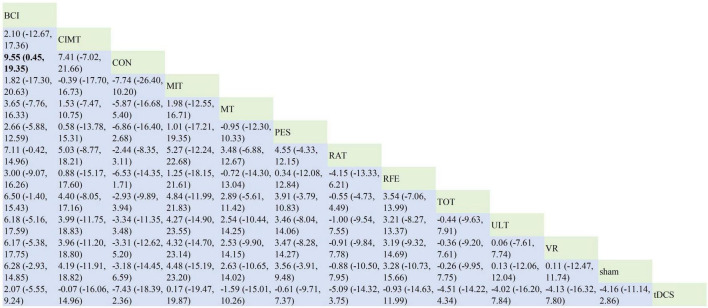
Network meta-analysis league table for ARAT. The results are reported based on the lower triangular matrix of the ADDIS league table. Therefore, effect estimates are expressed as column-defining treatments versus row-defining treatments (column vs. row). Bold values indicate statistically significant differences.

Network plots for the 11 interventions were generated using STATA 15.0 (Figure 3C). Based on PSRF values approaching 1 and the corresponding *p*-values (Supplementary Table 8), a consistency model was applied for the network meta-analysis. Ranking probability plots (Figure 8) and ranking probability tables (Table 1) were subsequently generated. The results showed that, compared with CON, only BCI demonstrated a statistically significant improvement in ARAT scores (MD = 9.55, 95% CI: 0.45–19.35), while the remaining interventions did not reach statistical significance. Compared with sham stimulation, none of the interventions showed statistically significant benefits. Although the ranking probabilities suggested that MIT, BCI, and CIMT had relatively higher rankings, these findings should be interpreted cautiously because no statistically significant differences were observed versus sham stimulation. Detailed network meta-analysis results are presented in Figure 9.

##### 3.5.3.3 Improvement in WMFT

Network plots for the 8 interventions were generated using STATA 15.0 (Figure 3D). The network structure showed that the interventions were divided into disconnected sub-networks due to the separation of CON and sham stimulation, and therefore a connected evidence loop could not be formed. As a result, a network meta-analysis under a consistency model and treatment ranking could not be performed for WMFT.

##### 3.5.3.4 Improvement in BBT

**FIGURE 10:**
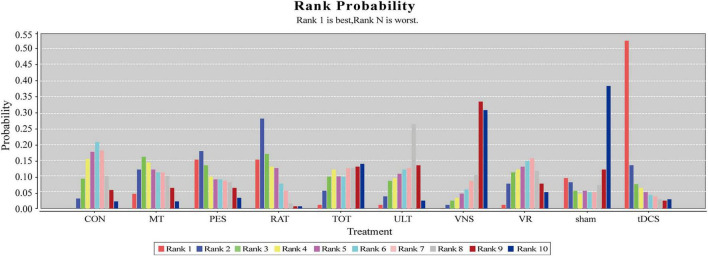
The figure of ranking probability of improvement in BBT.

**FIGURE 11:**
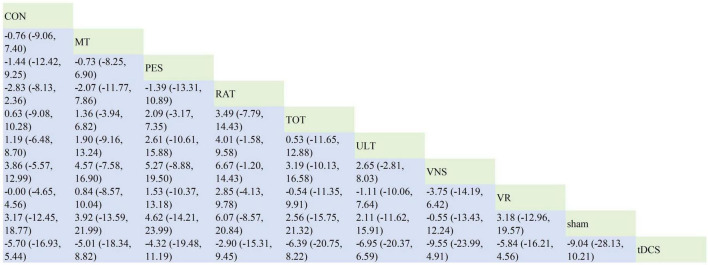
Network meta-analysis league table for BBT. The results are reported based on the lower triangular matrix of the ADDIS league table. Therefore, effect estimates are expressed as column-defining treatments versus row-defining treatments (column vs. row). Bold values indicate statistically significant differences.

Network plots for the 8 interventions were generated using STATA 15.0 (Figure 3E). Based on PSRF values approaching 1 (Supplementary Table 9), a consistency model was applied for the network meta-analysis. Because the BBT network did not contain any closed loops, node-splitting analysis could not be performed to assess local inconsistency. Ranking probability plots (Figure 10) and ranking probability tables (Table 1) were subsequently generated. The results showed that, compared with both CON and sham stimulation, none of the interventions demonstrated statistically significant improvements in BBT scores. Although the ranking probabilities suggested that tDCS, PES, and RAT had relatively higher rankings, these ranking results should be interpreted with caution because none of the interventions showed statistically significant superiority over the comparators, and inconsistency could not be formally assessed owing to the absence of closed loops in the network. Detailed network meta-analysis results are presented in Figure 11.

##### 3.5.3.5 Improvement in GS

Network plots for the eight interventions were generated using STATA 15.0 (Figure 3F). However, the evidence network was disconnected, comprising three independent subnetworks: The network was divided into three disconnected components: one including four studies with CON as the comparator (and a direct comparison between VR and TOT), one including two studies with sham stimulation as the comparator, and one consisting of a direct comparison between CIMT and MT. Because the network was not connected, network meta-analysis could not be performed. Consequently, relative treatment effects, treatment rankings, and inconsistency assessments could not be estimated for this outcome.

##### 3.5.3.6 Reduction in MAS

Network plots for the five interventions were generated using STATA 15.0 (Figure 3G). Although five interventions were identified, the network was divided into two disconnected components and therefore did not meet the requirements for a network meta-analysis. Consequently, neither network meta-analysis nor node-splitting analysis for the assessment of local inconsistency could be performed.

### 3.6 Subgroup analyses

#### 3.6.1 Stage of stroke

We conducted subgroup analyses for FMA-UE on 78 studies that clearly reported the stroke stage of participants, including 4 studies in the acute stage, 52 studies in the subacute stage, and 21 studies in the chronic stage, 1 study enrolled participants across all three stroke stages. Due to the limited number of studies in the acute stage, a network meta-analysis could not be performed for this subgroup. In patients in the subacute stage, compared with CON, CIMT, BCI, PES, and RAT demonstrated statistically significant improvements in FMA-UE. Among these interventions, CIMT (MD = 11.68, 95% CI: 6.22–17.04), BCI (MD = 7.97, 95% CI: 3.25–12.79), and PES (MD = 5.89, 95% CI: 0.80–11.29) showed relatively larger effect sizes. Compared with sham stimulation, CIMT, BCI, MT, PES, RAT, RFE, VR, TOT, and TMS demonstrated statistically significant benefits. CIMT (MD = 13.30, 95% CI: 6.81–19.59), BCI (MD = 9.59, 95% CI: 5.44–13.71), and TOT (MD = 7.67, 95% CI: 0.03–15.04) demonstrated relatively greater improvements. Moreover, regardless of whether compared with CON or sham stimulation, CIMT and BCI exceeded the MCID threshold, and when compared with sham stimulation, CIMT, BCI, PES, RFE, TOT, and VR also reached the MCID threshold, suggesting that these interventions may have potential clinical value for patients with subacute PS-ULMD. Detailed ranking probability plots and league tables are presented in Supplementary Figure 8.

For patients in the chronic stage, 21 studies were eligible for subgroup analysis. However, 4 studies used sham stimulation as the comparator, whereas the remaining 17 studies used CON. Because no intervention provided a connection between these two control conditions, the evidence network was separated into two disconnected subnetworks. Consequently, a network meta-analysis and treatment ranking could not be performed for the chronic-stage subgroup. Overall, these subgroup findings suggest that the effectiveness of non-pharmacological interventions may vary according to stroke stage.

#### 3.6.2 Severity of upper limb impairment

A total of 75 studies reported baseline FMA-UE scores of included patients, which were used to classify the severity of upper-limb impairment. Among these studies, 1 study involved patients with mild impairment, 51 involved patients with moderate impairment, and 22 involved patients with severe impairment, 1 study included patients across all severity levels. Due to the limited number of studies involving mild impairment, only the moderate and severe impairment subgroups were analyzed. In patients with moderate upper-limb impairment, compared with CON, CIMT (MD = 8.09, 95% CI: 3.95–12.00), BF (MD = 7.62, 95% CI: 0.57–14.61), and RAT (MD = 3.39, 95% CI: 0.42–6.37) demonstrated statistically significant improvements. Compared with sham stimulation, CIMT (MD = 11.05, 95% CI: 2.80–19.27), BF (MD = 10.52, 95% CI: 0.41–20.98), and BCI (MD = 9.94, 95% CI: 2.07–18.18) also showed statistically significant benefits. Moreover, CIMT and BF exceeded the MCID threshold regardless of comparison with CON or sham stimulation, while BCI also surpassed the MCID threshold compared with sham stimulation, suggesting their potential benefit in improving functional impairment in patients with moderate PS-ULMD. Detailed ranking probability plots and league tables are presented in Supplementary Figures 9A,B.

In patients with severe upper-limb impairment, compared with CON, VR demonstrated a relatively greater effect size (MD = 11.62, 95% CI: 1.80–21.25). Compared with sham stimulation, PES (MD = 9.76, 95% CI: 1.21–17.99) and BCI (MD = 8.96, 95% CI: 0.20–17.64) demonstrated a relatively greater improvement. Furthermore, the above-mentioned interventions all exceeded the MCID threshold, suggesting that these interventions may also provide potential benefits for patients with severe PS-ULMD. Overall, these findings suggest that the effectiveness of non-pharmacological interventions may vary according to the severity of upper-limb impairment. Detailed ranking probability plots and league tables are presented in Supplementary Figures 9C,D.

### 3.7 Sensitivity analyses

#### 3.7.1 Analysis restricted to sham-controlled studies

A total of 81 RCTs reported FMA-UE outcomes, of which 25 RCTs used sham stimulation as the control group and were included in this sensitivity analysis. Compared with sham stimulation, only PES and TMS demonstrated statistically significant improvements in upper-limb motor function. Notably, PES exceeded the MCID threshold, suggesting potential clinical value. Although the ranking probabilities suggested that PES, Acup, and MT were among the higher-ranked interventions, Acup and MT did not reach statistical significance. Detailed ranking probability plots and league tables are presented in Supplementary Figure 10. This analysis included only eight intervention nodes, with several interventions present in the primary network meta-analysis not included in this restricted analysis, which may have influenced the comparative rankings and effect estimates. Therefore, the ranking results should be interpreted with caution.

#### 3.7.2 Exclusion of studies with high risk of bias

A total of 81 RCTs reported effect sizes for FMA-UE outcomes, of which 24 studies were assessed as having a high risk of bias. Sensitivity analyses were subsequently conducted after excluding high-risk studies. The results showed that, compared with CON, BF, BCI, PES, MT, RAT, TOT, and VR demonstrated statistically significant improvements in FMA-UE scores. Among these interventions, BF, BCI, and PES showed relatively large effect sizes. Compared with sham stimulation, BCI, PES, MT, and TMS achieved statistically significant effects, with BCI, PES, and MT demonstrating comparatively greater effect sizes. Detailed ranking probability plots and league tables are presented in Supplementary Figure 11. Overall, the findings after exclusion of studies with high risk of bias were largely consistent with those of the primary analysis, with BCI, BF, and PES continuing to demonstrate relatively large effect sizes. Minor changes in treatment rankings may be attributable to alterations in the network structure and reduction in available evidence following study exclusion.

#### 3.7.3 Exclusion of studies including recurrent stroke patients

A total of 60 studies exclusively included patients with first-ever stroke and were included in this sensitivity analysis. Compared with CON, CIMT, PES, BF, BCI, VR, RFE, and RAT demonstrated statistically significant improvements in upper-limb motor function. Among these interventions, CIMT, PES, and BF showed relatively larger effect sizes. Compared with sham stimulation, CIMT, PES, BF, BCI, VR, RFE, RAT, MT, MIT, ULT, and VNS demonstrated statistically significant benefits. CIMT, PES and BF demonstrated relatively greater improvements. Detailed ranking probability plots and league tables are presented in Supplementary Figure 12. The findings in patients with first-ever stroke were largely consistent with those of the primary analysis, with CIMT, PES, BF, and BCI continuing to show prominent treatment effects.

#### 3.7.4 Analysis restricted to add-on effects and monotherapy interventions

A total of 38 studies investigated add-on interventions based on CON. For add-on interventions, compared with sham stimulation, CIMT, BCI, MT, and TMS demonstrated statistically significant improvements in upper-limb motor function. Among these interventions, CIMT, BCI, and MT showed relatively larger effect sizes. Detailed ranking probability plots and league tables are presented in Supplementary Figure 13.

A total of 43 studies evaluated monotherapy interventions. The network meta-analysis showed that, compared with CON, BF, CIMT, VR, RFE, and RAT demonstrated statistically significant improvements in upper-limb motor function. Among these interventions, BF, CIMT, and VR showed relatively larger effect sizes. Compared with sham stimulation, BCI, BF, CIMT, MIT, MT, PES, RAT, RFE, TOT, ULT, and VR demonstrated statistically significant benefits. Among these interventions, BCI, BF, and ULT demonstrated relatively greater improvements. Detailed ranking probability plots and league tables are presented in Supplementary Figure 14. Overall, the findings remained generally consistent with the primary analysis, although some variations in treatment rankings were observed between add-on and monotherapy settings.

### 3.8 Publication bias

#### 3.8.1 Pairwise meta-analysis

Funnel plots were constructed for each outcome using CON or sham as the comparator group (Supplementary Figure 15). For FMA-UE, most studies were distributed in the upper region and symmetrically around the vertical line, suggesting no obvious publication bias. Funnel plots for the remaining outcomes were generally symmetrical; however, the limited number of included studies may have restricted the interpretability of these results.

#### 3.8.2 Network meta-analysis

**FIGURE 12:**
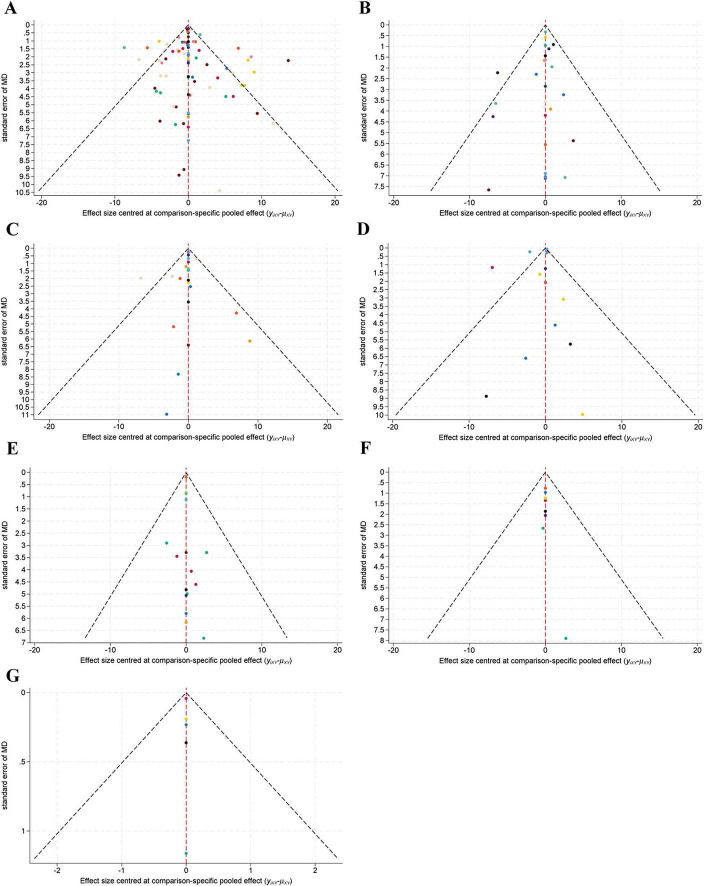
Funnel plot for the network meta-analysis of outcomes. **(A)** Funnel plot for the network meta-analysis of improvement in FMA-UE, **(B)** Funnel plot for the network meta-analysis of improvement in ADL; **(C)** Funnel plot for the network meta-analysis of improvement in ARAT, **(D)** Funnel plot for the network meta-analysis of improvement in WMFT. **(E)** Funnel plot for the network meta-analysis of improvement in BBT. **(F)** Funnel plot for the network meta-analysis of improvement in GS. **(G)** Funnel plot for the network meta-analysis of reduction in MAS.

Comparison-adjusted funnel plots were constructed to evaluate potential publication bias in the network meta-analysis (Figure 12). For FMA-UE, most studies were symmetrically distributed around the vertical line and fell within the pseudo 95% confidence limits. Although a few studies were located outside the confidence limits, the overall distribution appeared symmetrical, suggesting no substantial publication bias. For ADL, ARAT, WMFT, BBT, GS, and MAS, the majority of studies were distributed within the funnel region, with only a few studies falling outside the boundaries, and some outcomes showed complete distribution within the funnel boundaries. However, because the number of included studies for several secondary outcomes was relatively small, the interpretability of these funnel plots was limited. Overall, no substantial evidence of publication bias or small-study effects was observed.

### 3.9 Certainty of evidence

The certainty of evidence assessed using the CINeMA framework ranged from high to very low across outcomes, with detailed results presented in Supplementary Table 10. Overall, most comparisons were rated as having low or very low certainty, mainly due to within-study bias, heterogeneity, and imprecision, whereas concerns regarding indirectness, incoherence, and reporting bias were generally minimal. For WMFT, GS and MAS, the small number of studies and the absence of closed loops in the treatment networks precluded a comprehensive CINeMA assessment.

### 3.10 Adverse events

A total of 32 studies reported information related to adverse events, among which 23 studies reported no adverse events. In studies involving TMS, one study (Sharma et al., 2020) reported a seizure in one patient in the intervention group, while another study (Sun et al., 2024) reported headache in four patients, all of which resolved spontaneously. In studies involving tDCS, one study (NETS Trial Collaboration Group, 2024) reported pain in one patient in the intervention group and one patient in the control group. Among studies involving VNS, one study (Dawson et al., 2021) reported vocal cord paralysis in one patient in the control group. In a study involving CIMT (Thrane et al., 2015), two patients experienced shoulder pain. In studies involving VR (Han et al., 2016), one patient in the intervention group developed shoulder pain, while one patient in the control group experienced worsening of symptoms. In addition, three studies (Adie et al., 2017; Kimberley et al., 2018; Rémy-Néris et al., 2021) reported adverse events that were considered unrelated to the interventions. The remaining studies did not report safety outcomes or adverse event-related information.

## 4 Discussion

### 4.1 Principal findings

Compared with previous NMAs, the present study provides a more comprehensive comparison of multiple non-pharmacological interventions for PS-ULMD by incorporating a broader range of interventions, Chinese-language studies, and multiple outcome measures. In this study, our findings are consistent with those of previous studies (Saikaley et al., 2022; Tenberg et al., 2023; Zheng et al., 2025), further corroborating the effectiveness of several non-pharmacological interventions (e.g., BCI, CIMT, BF) in improving PS-ULMD. Importantly, these improvements may also be clinically meaningful. For example, several interventions, including BCI, CIMT, BF, and PES, demonstrated MD values that substantially exceeded the reported MCID threshold of 6.0 for FMA-UE (Page et al., 2012), suggesting potentially meaningful clinical improvements. Nevertheless, several limitations remain in previous NMAs. For example, one network meta-analysis evaluated the effectiveness of non-conventional therapies for PS-ULMD (Saikaley et al., 2022), but relied on a single outcome measure and did not include conventional interventions, whose therapeutic value in PS-ULMD should not be overlooked. Other studies (Tenberg et al., 2023; Zheng et al., 2025) primarily conducted network meta-analyses focusing on subtypes within a single intervention modality, rather than providing a comprehensive comparison across multiple non-pharmacological interventions. In light of these limitations, our network meta-analysis, which included 89 RCTs involving 5129 participants, provides several advances: (I) we evaluated the overall effectiveness of non-pharmacological interventions rather than focusing on individual therapies alone; (II) we incorporated multiple outcome measures, with FMA-UE as the primary endpoint, and conducted ranking analyses to provide exploratory insights into the comparative effectiveness of interventions across different functional domains. (III) we incorporated MCID thresholds for FMA-UE to help translate statistical effect estimates into clinically meaningful interpretations, thereby enhancing the clinical relevance of the findings.

### 4.2 Clinical implications

Clinically, our findings indicate that non-pharmacological interventions can significantly improve upper limb motor function in patients with PS-ULMD. These findings may help clinicians better understand the comparative effectiveness of different interventions across functional domains and support individualized rehabilitation planning. Some interventions, including BCI, CIMT, BF and PES, demonstrated relatively greater improvements in overall upper limb motor function, whereas other interventions appeared to have advantages in specific functional domains. In clinical rehabilitation planning, clinicians should also consider factors such as feasibility, cost, accessibility, patient adherence, and long-term sustainability of treatment effects when selecting intervention strategies. For example, interventions such as BCI and RAT may require specialized equipment and substantial healthcare resources, potentially limiting their accessibility in certain rehabilitation settings, whereas interventions such as MT and CIMT may be more feasible in routine clinical practice. In addition, these findings also inform healthcare researchers in the rational allocation of research resources. For example, our findings indicate that RAT yielded comparatively smaller improvements in overall upper limb motor function than BF, CIMT, and MIT, which is consistent with previous evidence (Saikaley et al., 2022). Despite being extensively studied, the therapeutic effects of RAT may be less pronounced than those of other non-pharmacological interventions, suggesting that allocating limited research resources to alternative non-pharmacological interventions may provide more informative guidance for clinical practice. Furthermore, these results also provide insights into potential combination strategies for non-pharmacological interventions, considering cost-effectiveness and practical feasibility, such as integrating BCI with CIMT or PES to maximize therapeutic benefits. Given that the certainty of evidence for most outcomes was low or very low, the findings should be interpreted with caution but may serve as a reference for rehabilitation planning. Further well-designed RCTs are needed to confirm these effects and support clinical implementation.

### 4.3 Implications for future research

The findings of this network meta-analysis highlight several important directions for future research in the field of post-stroke upper limb motor rehabilitation. First, there is a clear need for well-designed, adequately powered RCTs with rigorous methodological quality to improve the certainty of evidence. Future studies should ensure appropriate randomization, allocation concealment, protocol registration, and standardized reporting to reduce the risk of bias observed in existing trials (Chan et al., 2013; Schulz et al., 2010). Second, greater consistency in outcome selection is warranted. Given its widespread use and clinical relevance, FMA-UE should be prioritized as a core outcome measure to enhance comparability across studies, while additional functional outcomes may be included to capture complementary dimensions of upper limb recovery. Third, future studies on PS-ULMD should provide more detailed reporting of key clinical characteristics, including stroke stage and severity, as well as safety outcomes and adverse events, to improve the assessment of clinical heterogeneity and the transitivity assumption in network meta-analyses. Fourth, interventions that demonstrated relatively favorable effects or higher ranking probabilities in this analysis, such as BCI, BF, and CIMT, merit further validation through head-to-head trials with sufficient sample sizes. Finally, future research should pay greater attention to intervention dosage, treatment protocols, and follow-up duration. Standardization of intervention parameters and inclusion of longer-term outcomes would facilitate reproducibility and support the translation of research findings into clinical practice.

### 4.4 Strengths and limitations

This network meta-analysis has several strengths. First, by incorporating multidimensional outcome measures, this study provides a comprehensive synthesis of the effectiveness evidence for a broad range of non-pharmacological interventions recommended in current clinical guidelines. Second, we systematically searched multiple Chinese and English databases, thereby incorporating evidence from both Chinese- and English-language literature. This approach enhances the geographical and cultural generalizability of our findings. Third, by applying a network meta-analytic framework, we integrated both direct and indirect evidence to simultaneously compare multiple non-pharmacological therapies against CON or sham stimulation across several clinically relevant outcomes. This approach allows for a more comprehensive and clinically meaningful evaluation of the relative effectiveness of commonly used rehabilitation interventions for PS-ULMD. Finally, we incorporated MCID thresholds for FMA-UE to facilitate the interpretation of statistical effect estimates from a clinical perspective, thereby enhancing the clinical relevance and applicability of the findings.

However, this study has several limitations. First, only studies published in English and Chinese were included, which may have introduced language bias and resulted in the omission of relevant studies published in other languages. Second, although interventions were categorized into distinct nodes for network meta-analysis, substantial clinical heterogeneity may exist within each intervention category, particularly regarding treatment protocols, stimulation parameters, training frequency, and session duration. In addition, treatment dose and intervention duration were not quantitatively modeled because of inconsistent reporting across studies. Moreover, the TMS node was represented exclusively by low-frequency (1 Hz) stimulation protocols because of the limited availability of eligible studies, whereas high-frequency TMS protocols were not included in the analysis. Therefore, the estimated effects of TMS may not be generalizable to other stimulation frequencies, and the conclusions regarding TMS should be considered specific to low-frequency (1 Hz) stimulation protocols. Third, the distribution of studies across stroke stages was unbalanced, with most included studies enrolling patients in the subacute stage. Owing to the limited number of studies in the acute stage and the disconnected evidence network in the chronic stage, subgroup network meta-analysis could only be conducted for subacute-stage patients. Therefore, the findings of this study may be more applicable to patients in the subacute stage, whereas the comparative effectiveness of non-pharmacological interventions in acute and chronic stroke populations remains uncertain. Further high-quality studies focusing on these patient groups are warranted. Fourth, long-term follow-up outcomes were insufficiently reported in most included studies, limiting the ability to determine whether the observed therapeutic benefits could be maintained over time. Fifth, although adverse events and safety outcomes were summarized, the reporting quality varied considerably across studies, and several trials lacked detailed safety assessments. In addition, the overall certainty of evidence was predominantly rated as low to very low for most comparisons, mainly due to study limitations, imprecision, and inconsistency, which may limit the strength of the conclusions. Finally, Due to the limited number of available studies, the evidence networks for WMFT, GS, and MAS were disconnected and therefore could not support network meta-analysis. Consequently, the comparative effectiveness of interventions for these outcomes could not be comprehensively evaluated. Furthermore, some intervention rankings may be unstable because certain comparisons were supported by only a limited number of studies.

## 5 Conclusion

In summary, the present study demonstrates that multiple non-pharmacological interventions can improve upper limb function in patients with PS-ULMD to varying degrees. Among the evaluated interventions, BCI, CIMT, BF, and PES appeared to be associated with relatively larger improvements in FMA-UE. However, given the predominantly low to very low certainty of the overall evidence, these findings should be interpreted with caution, and further high-quality RCTs are warranted to confirm and refine these results.

## Data Availability

The original contributions presented in this study are included in the article/Supplementary material, further inquiries can be directed to the corresponding authors.
