# Supplementary material for: Comparative effectiveness of non-pharmacological interventions for post-stroke upper limb motor dysfunction: a systematic review and network meta-analysis of randomized controlled trials
Source: Front Aging Neurosci. 2026 Jul 3;18:1852556. doi: 10.3389/fnagi.2026.1852556 (PMC13375904; doi:10.3389/fnagi.2026.1852556)
Supplement: Supplementary file 1 [file Table_1.docx]

| Supplemental materials | Page |
| --- | --- |
| Appendix S1. Search strategies of each database. | 2-12 |
| Figure S1. Forest plot of FMA-UE | 13-14 |
| Figure S2. Forest plot of ADL | 15 |
| Figure S3. Forest plot of ARAT | 16 |
| Figure S4. Forest plot of WMFT | 17 |
| Figure S5. Forest plot of BBT | 18 |
| Figure S6. Forest plot of GS | 19 |
| Figure S7. Forest plot of MAS | 20 |
| Figure S8. Network Meta-analysis Results for Subgroup Analysis by Disease Stage: (A) ranking probability plot; (B) league table | 21-22 |
| Figure S9. Network Meta-analysis Results for Subgroup Analysis by Disease Severity: (A) Ranking Probability Plot of Moderate; (B) League Table of Moderate; (C)Ranking Probability Plot of Severe; (D)League Table of Severe | 22-26 |
| Figure S10. Sensitivity analysis restricted to sham-controlled studies: (A) ranking probability plot; (B) league table | 26-28 |
| Figure S11. Sensitivity Analysis Excluding Studies with High Risk of Bias: (A) ranking probability plot; (B) league table | 28-30 |
| Figure S12. Sensitivity Analysis Excluding Studies Including Recurrent Stroke Patients: (A) ranking probability plot; (B) league table | 30-32 |
| Figure S13. Sensitivity Analysis Restricted to Add-On Effect Studies: (A) ranking probability plot; (B) league table | 32-34 |
| Figure S14. Sensitivity Analysis Restricted to Monotherapy Studies: (A) ranking probability plot; (B) league table | 35-36 |
| Figure S15. Funnel plot of FMA-UE, ADL, ARAT, WMFT, BBT, GS and MAS. | 37-39 |
| Table S1. PRISMA NMA Checklist of Items to Include When Reporting A Systematic Review Involving a Network Meta-analysis | 39-43 |
| Table S2. Information Related to 15 Interventions. | 43-46 |
| Table S3. Summary table of stimulation parameters | 47-48 |
| Table S4. Main characteristics of included RCTs. | 49-59 |
| Table S5. Risk of bias summary | 60-63 |
| Table S6. The PSRF value and Node-splitting test result of improvement in FMA-UE | 64-66 |
| Table S7. The PSRF value and Node-splitting test result of improvement in ADL | 66-67 |
| Table S8. The PSRF value and Node-splitting test result of improvement in ARAT | 68-69 |
| Table S9. The PSRF value of improvement in BBT | 69 |
| Table S10. Summary table for credibility assessment using confidence in network meta-analysis (CINeMA) for the FMA-UE, ADL, ARAT and BBT | 70-72 |

**Appendix S1. Search strategies of each database.**

**PubMed：**

#1 (Upper Limb Training[Title/Abstract]) OR (Task Performance and Analysis[Mesh]) OR (Task-specific training[Title/Abstract]) OR (Repetitive task practice[Title/Abstract]) OR (Constraint-Induced Movement Therapy[Mesh]) OR (Bilateral Arm Training[Title/Abstract]) OR (Bimanual Training[Title/Abstract]) OR (Peripheral electrical stimulation[Title/Abstract]) OR (Functional Electrical Stimulation[Title/Abstract]) OR (Transcutaneous Electric Nerve Stimulation[Title/Abstract]) OR (Neuromuscular Electrical Stimulation[Title/Abstract]) OR (transcranial direct current stimulation[Title/Abstract]) OR (Vagus Nerve Stimulation[Title/Abstract]) OR (Transcranial Magnetic Stimulation[Mesh]) OR (repetitive transcranial magnetic stimulation[Title/Abstract]) OR (Mirror Movement Therapy[Mesh]) OR (Mirror Therapy[Title/Abstract]) OR (Virtual Reality Exposure Therapy[Mesh]) OR (Virtual Reality Therapy[Title/Abstract]) OR (Virtual Reality[Title/Abstract]) OR (Biofeedback[Title/Abstract]) OR (Robotics[Mesh]) OR (Robot-Assisted Therapy[Title/Abstract]) OR (Robotic Rehabilitation[Title/Abstract]) OR (robot-assisted movement therapy[Title/Abstract]) OR (robot-assisted[Title/Abstract]) OR (Acupuncture Therapy[Mesh]) OR (Acupuncture[Title/Abstract]) OR (motor imagery training[Title/Abstract]) OR (mental imagery[Title/Abstract]) OR (occupational therapy[Title/Abstract]) OR (physical therapy[Title/Abstract]) OR (action observation[Title/Abstract]) OR (mental practice[Title/Abstract]) OR (non-invasive brain stimulation[Title/Abstract]) OR (brain computer interface[Title/Abstract]) OR (exoskeleton [Title/Abstract]) OR (telerehabilitation[Title/Abstract]) OR (augmented reality [Title/Abstract])

#2 (Stroke[MeSH Terms]) OR (stroke[Title/Abstract]) OR (Strokes[Title/Abstract]) OR (Cerebrovascular Accident[Title/Abstract]) OR (Cerebrovascular Accidents[Title/Abstract]) OR (Cerebral Stroke[Title/Abstract]) OR (Cerebral Strokes[Title/Abstract]) OR (Stroke, Cerebral[Title/Abstract]) OR (Strokes, Cerebral[Title/Abstract]) OR (Cerebrovascular Apoplexy[Title/Abstract]) OR (Apoplexy, Cerebrovascular[Title/Abstract]) OR (Vascular Accident, Brain[Title/Abstract]) OR (Brain Vascular Accident[Title/Abstract]) OR (Brain Vascular Accidents[Title/Abstract]) OR (Vascular Accidents, Brain[Title/Abstract]) OR (Cerebrovascular Stroke[Title/Abstract]) OR (Cerebrovascular Strokes[Title/Abstract]) OR (Stroke, Cerebrovascular[Title/Abstract]) OR (Strokes, Cerebrovascular[Title/Abstract]) OR (Apoplexy[Title/Abstract]) OR (CVA (Cerebrovascular Accident[Title/Abstract])) OR (CVAs (Cerebrovascular Accident[Title/Abstract])) OR (Stroke, Acute[Title/Abstract]) OR (Acute Stroke[Title/Abstract]) OR (Acute Strokes[Title/Abstract]) OR (Strokes, Acute[Title/Abstract]) OR (Cerebrovascular Accident, Acute[Title/Abstract]) OR (Acute Cerebrovascular Accident[Title/Abstract]) OR (Acute Cerebrovascular Accidents[Title/Abstract]) OR (Cerebrovascular Accidents, Acute[Title/Abstract]) OR (Brain Infarction[Title/Abstract]) OR (Cerebral Infarction [Title/Abstract]) OR (Hemorrhagic Stroke[Title/Abstract]) OR (Ischemic Stroke[Title/Abstract]) OR (Embolic Stroke[Title/Abstract]) OR (Thrombotic Stroke [Title/Abstract])

#3 (Upper extremity [MeSH Terms]) OR (Upper extremity [Title/Abstract]) OR (upper limb [Title/Abstract]) OR (arm [Title/Abstract]) OR (Axilla [Title/Abstract]) OR (elbow [Title/Abstract]) OR (Forearm [Title/Abstract]) OR (hand [Title/Abstract]) OR (wrist [Title/Abstract]) OR (shoulder [Title/Abstract]) OR (finger [Title/Abstract]) OR (arm function[Title/Abstract]) OR (hand function[Title/Abstract])

#4 (Dyskinesias[MeSH Terms]) OR (Dyskinesia[Title/Abstract]) OR (dyskinesias[Title/Abstract]) OR (Abnormal Movement[Title/Abstract]) OR (Abnormal Movements[Title/Abstract]) OR (Paralyses[Title/Abstract]) OR (Plegia[Title/Abstract]) OR (Plegias[Title/Abstract]) OR (Palsy[Title/Abstract]) OR (Palsies[Title/Abstract]) OR (spasm[Title/Abstract]) OR (spasms[Title/Abstract]) OR (Spastic Paraparesis[Title/Abstract]) OR (Spastic Parapareses[Title/Abstract]) OR (motor function[Title/Abstract]) OR (motor impairment[Title/Abstract]) OR (Motor Disorder[Title/Abstract]) OR (Movement Disorder[Title/Abstract]) OR (Movement impairment[Title/Abstract]) OR (Movement function[Title/Abstract]) OR (motor recovery[Title/Abstract]) OR (neurorehabilitation[Title/Abstract]) OR (hemiparesis [Title/Abstract]) OR (hemiplegia[Title/Abstract]) OR (paresis[Title/Abstract])

#5 (clinical trials, randomized [MeSH Terms]) OR (controlled clinical trials, randomized [MeSH Terms]) OR (clinical trials as topic [MeSH Terms]) OR (random allocation [MeSH Terms]) OR ((clinical [Title/Abstract]) OR (trial [Title/Abstract])) OR (random*[Title/Abstract]) OR (clinical trial [Publication Type])

#6 #1 AND #2 AND #3 AND #4 AND #5

**Embase**

#1 'Upper Limb Training':ti,ab,kw OR 'Task Performance and Analysis'/exp OR 'Task-specific training':ti,ab,kw OR 'Repetitive task practice':ti,ab,kw OR 'Constraint-Induced Movement Therapy'/exp OR 'Bilateral Arm Training':ti,ab,kw OR 'Bimanual Training':ti,ab,kw OR 'Peripheral electrical stimulation':ti,ab,kw OR 'Functional Electrical Stimulation':ti,ab,kw OR 'Transcutaneous Electric Nerve Stimulation':ti,ab,kw OR 'Neuromuscular Electrical Stimulation':ti,ab,kw OR 'transcranial direct current stimulation':ti,ab,kw OR 'Vagus nerve stimulation':ti,ab,kw OR 'Transcranial Magnetic Stimulation'/exp OR 'repetitive transcranial magnetic stimulation':ti,ab,kw  OR 'Mirror Movement Therapy'/exp OR 'Mirror Therapy':ti,ab,kw OR 'Virtual Reality Exposure Therapy'/exp OR 'Virtual Reality Therapy':ti,ab,kw OR 'Virtual Reality':ti,ab,kw OR 'Biofeedback':ti,ab,kw OR 'Robotics'/exp OR 'Robot-Assisted Therapy':ti,ab,kw OR 'Robotic Rehabilitation':ti,ab,kw OR 'robot-assisted movement therapy':ti,ab,kw OR 'robot-assisted':ti,ab,kw OR 'Acupuncture Therapy'/exp OR 'Acupuncture':ti,ab,kw OR 'motor imagery training':ti,ab,kw OR 'mental imagery':ti,ab,kw OR 'occupational therapy':ti,ab,kw OR 'physical therapy'/exp OR 'action observation':ti,ab,kw OR 'mental practice':ti,ab,kw OR 'non-invasive brain stimulation':ti,ab,kw OR 'brain computer interface':ti,ab,kw OR 'exoskeleton':ti,ab,kw OR 'telerehabilitation':ti,ab,kw OR 'augmented reality':ti,ab,kw

#2 'stroke'/exp OR 'stroke':ti,ab,kw OR 'strokes':ti,ab,kw OR 'cerebrovascular accident':ti,ab,kw OR 'cerebrovascular accidents':ti,ab,kw OR 'cerebral stroke':ti,ab,kw OR 'cerebral strokes':ti,ab,kw OR 'cerebrovascular apoplexy':ti,ab,kw OR 'brain vascular accident':ti,ab,kw OR 'brain vascular accidents':ti,ab,kw OR 'cerebrovascular stroke':ti,ab,kw OR 'cerebrovascular strokes':ti,ab,kw OR 'apoplexy':ti,ab,kw OR 'cva cerebrovascular accident':ti,ab,kw OR 'cvas cerebrovascular accident':ti,ab,kw OR 'acute stroke':ti,ab,kw OR 'acute strokes':ti,ab,kw OR 'acute cerebrovascular accident':ti,ab,kw OR 'acute cerebrovascular accidents':ti,ab,kw OR 'brain infarction':ti,ab,kw OR 'cerebral infarction':ti,ab,kw OR 'hemorrhagic stroke':ti,ab,kw OR 'ischemic stroke':ti,ab,kw OR 'embolic stroke':ti,ab,kw OR 'thrombotic stroke':ti,ab,kw

#3 'Upper extremity'/exp OR 'Upper extremity':ti,ab,kw OR 'upper limb':ti,ab,kw OR 'arm':ti,ab,kw OR 'Axilla':ti,ab,kw OR 'elbow':ti,ab,kw OR 'Forearm':ti,ab,kw OR 'hand':ti,ab,kw OR 'wrist':ti,ab,kw OR 'shoulder':ti,ab,kw OR 'finger':ti,ab,kw OR 'arm function':ti,ab,kw OR 'hand function':ti,ab,kw

#4 'dyskinesia'/exp OR 'dyskinesia':ti,ab,kw OR 'dyskinesias':ti,ab,kw OR 'abnormal movement':ti,ab,kw OR 'abnormal movements':ti,ab,kw OR 'paralyses':ti,ab,kw OR 'plegia':ti,ab,kw OR 'plegias':ti,ab,kw OR 'palsy':ti,ab,kw OR 'palsies':ti,ab,kw OR 'spasm':ti,ab,kw OR 'spasms':ti,ab,kw OR 'spastic paraparesis':ti,ab,kw OR 'spastic parapareses':ti,ab,kw OR 'motor function':ti,ab,kw OR 'motor impairment':ti,ab,kw OR 'motor disorder':ti,ab,kw OR 'movement disorder':ti,ab,kw OR 'movement impairment':ti,ab,kw OR 'movement function':ti,ab,kw OR 'motor recovery':ti,ab,kw OR 'neurorehabilitation':ti,ab,kw OR 'hemiparesis':ti,ab,kw OR 'hemiplegia':ti,ab,kw OR 'paresis':ti,ab,kw

#5 'clinical trials as topic'/exp OR 'clinical studies as topic'/exp OR 'controlled clinical trials as topic'/exp OR 'random allocation':ti,ab,kw OR ('clinical':ti,ab,kw AND 'trial':ti,ab,kw) OR 'clinical trial':ti,ab,kw OR 'random*':ti,ab,kw

#6 #1 AND #2 AND #3 AND #4 AND #5

**Web of Science**

#1 TS= (Upper Limb Training OR ULT OR Task Performance and Analysis OR Task-specific training OR Repetitive task practice OR Constraint-Induced Movement Therapy OR Bilateral Arm Training OR Bimanual Training OR Peripheral electrical stimulation OR Functional Electrical Stimulation OR Transcutaneous Electric Nerve Stimulation OR Neuromuscular Electrical Stimulation OR transcranial direct current stimulation OR Vagus Nerve Stimulation OR Transcranial Magnetic Stimulation OR repetitive transcranial magnetic stimulation OR Mirror Movement Therapy OR Mirror Therapy OR Virtual Reality Exposure Therapy OR Virtual Reality Therapy OR Virtual Reality OR Biofeedback OR Robotics OR Robot-Assisted Therapy OR Robotic Rehabilitation OR robot-assisted movement therapy OR robot-assisted OR Acupuncture Therapy OR Acupuncture OR motor imagery training OR mental imagery OR occupational therapy OR physical therapy OR action observation OR mental practice OR non-invasive brain stimulation OR brain computer interface OR exoskeleton OR telerehabilitation OR augmented reality)

#2 TS= (stroke OR Strokes OR Cerebrovascular Accident OR Cerebrovascular Accidents OR Cerebral Stroke OR Cerebral Strokes OR Cerebrovascular Apoplexy OR Brain Vascular Accident OR Brain Vascular Accidents OR Cerebrovascular Stroke OR Cerebrovascular Strokes OR Apoplexy OR CVA Cerebrovascular Accident OR CVAs Cerebrovascular Accident OR Acute Stroke OR Acute Strokes OR Acute Cerebrovascular Accident OR Acute Cerebrovascular Accidents OR Brain Infarction OR Cerebral Infarction OR Hemorrhagic Stroke OR Ischemic Stroke OR Embolic Stroke OR Thrombotic Stroke)

#3 TS= (Upper extremity OR upper limb OR arm OR Axilla OR elbow OR Forearm OR hand OR wrist OR shoulder OR finger OR arm function OR hand function)

#4 TS= (Dyskinesia OR dyskinesias OR Abnormal Movement OR Abnormal Movements OR Paralyses OR Plegia OR Plegias OR Palsy OR Palsies OR spasm OR spasms OR Spastic Paraparesis OR Spastic Parapareses OR motor function OR motor impairment OR Motor Disorder OR Movement Disorder OR Movement impairment OR Movement function OR motor recovery OR neurorehabilitation OR hemiparesis OR hemiplegia OR paresis)

#5 TS= (clinical trials as topic OR Clinical Studies as Topic OR Controlled Clinical Trials as Topic OR random allocation OR (clinical AND trial) OR clinical trial OR random*)

#6 #1 and #2 and #3 and #4 and #5

**Cochrane library**

#1 MeSH descriptor: [Task Performance and Analysis] explode all trees

#2 MeSH descriptor: [Transcranial Magnetic Stimulation] explode all trees

#3 MeSH descriptor: [Mirror Movement Therapy] explode all trees

#4 MeSH descriptor: [Virtual Reality Exposure Therapy] explode all trees

#5 MeSH descriptor: [Robotics] explode all trees

#6 MeSH descriptor: [Acupuncture Therapy] explode all trees

#7 MeSH descriptor: [Transcranial Direct Current Stimulation] explode all trees

#8 MeSH descriptor: [Transcutaneous Electric Nerve Stimulation] explode all trees

#9 MeSH descriptor: [Transcranial Direct Current Stimulation] explode all trees

#10 MeSH descriptor: [Biofeedback, Psychology] explode all trees

#11 MeSH descriptor: [occupational therapy] explode all trees

#12 MeSH descriptor: [physical therapy] explode all trees

#13 MeSH descriptor: [brain computer interface] explode all trees

#14 MeSH descriptor: [exoskeleton] explode all trees

#15 MeSH descriptor: [telerehabilitation] explode all trees

#16 MeSH descriptor: [augmented reality] explode all trees

#17 (Upper Limb Training):ti,ab,kw OR (Task Performance and Analysis):ti,ab,kw OR (Task-specific training):ti,ab,kw OR (Repetitive task practice):ti,ab,kw OR (Constraint-Induced Movement Therapy):ti,ab,kw OR (Bilateral Arm Training):ti,ab,kw OR (Bimanual Training):ti,ab,kw OR (Peripheral electrical stimulation):ti,ab,kw OR (Functional Electrical Stimulation):ti,ab,kw OR (Transcutaneous Electric Nerve Stimulation):ti,ab,kw OR (Neuromuscular Electrical Stimulation):ti,ab,kw OR (transcranial direct current stimulation):ti,ab,kw OR (Vagus Nerve Stimulation):ti,ab,kw OR (Transcranial Magnetic Stimulation):ti,ab,kw OR (repetitive transcranial magnetic stimulation):ti,ab,kw OR (Mirror Movement Therapy):ti,ab,kw OR (Mirror Therapy):ti,ab,kw OR (Virtual Reality Exposure Therapy):ti,ab,kw OR (Virtual Reality Therapy):ti,ab,kw OR (Virtual Reality):ti,ab,kw OR (Biofeedback):ti,ab,kw OR (Robotics):ti,ab,kw OR (Robot-Assisted Therapy):ti,ab,kw OR (Robotic Rehabilitation):ti,ab,kw OR (robot-assisted movement therapy):ti,ab,kw OR (robot-assisted):ti,ab,kw OR (Acupuncture Therapy):ti,ab,kw OR (Acupuncture):ti,ab,kw OR (motor imagery training):ti,ab,kw OR (mental imagery):ti,ab,kw OR (action observation):ti,ab,kw OR (mental practice):ti,ab,kw OR (non-invasive brain stimulation):ti,ab,kw

#18 #1 OR #2 OR #3 OR #4 OR #5 OR #6 OR #7 OR #8 OR #9 OR #10 OR #11 OR #12 OR #13 OR #14 OR #15 OR #16 OR #17

#19 MeSH descriptor: [Stroke] explode all trees

#20 (stroke):ti,ab,kw OR (strokes):ti,ab,kw OR (cerebrovascular accident):ti,ab,kw OR (cerebrovascular accidents):ti,ab,kw OR (cerebral stroke):ti,ab,kw OR (cerebral strokes):ti,ab,kw OR (cerebrovascular apoplexy):ti,ab,kw OR (brain vascular accident):ti,ab,kw OR (brain vascular accidents):ti,ab,kw OR (cerebrovascular stroke):ti,ab,kw OR (cerebrovascular strokes):ti,ab,kw OR (apoplexy):ti,ab,kw OR (cva cerebrovascular accident):ti,ab,kw OR (cvas cerebrovascular accident):ti,ab,kw OR (acute stroke):ti,ab,kw OR (acute strokes):ti,ab,kw OR (acute cerebrovascular accident):ti,ab,kw OR (acute cerebrovascular accidents):ti,ab,kw OR (brain infarction):ti,ab,kw OR (cerebral infarction):ti,ab,kw OR (hemorrhagic stroke):ti,ab,kw OR (ischemic stroke):ti,ab,kw OR (embolic stroke):ti,ab,kw OR (thrombotic stroke):ti,ab,kw

#21 #19 OR #20

#22 MeSH descriptor: [Upper Extremity] explode all trees

#23 (Upper extremity):ti,ab,kw OR (upper limb):ti,ab,kw OR (arm):ti,ab,kw OR (Axilla):ti,ab,kw OR (elbow):ti,ab,kw OR (Forearm):ti,ab,kw OR (hand):ti,ab,kw OR (wrist):ti,ab,kw OR (shoulder):ti,ab,kw OR (finger):ti,ab,kw OR (arm function):ti,ab,kw OR (hand function):ti,ab,kw

#24 #22 OR #23

#25 MeSH descriptor: [Dyskinesias] explode all trees

#26 (dyskinesia):ti,ab,kw OR (dyskinesias):ti,ab,kw OR (abnormal movement):ti,ab,kw OR (abnormal movements):ti,ab,kw OR (paralyses):ti,ab,kw OR (plegia):ti,ab,kw OR (plegias):ti,ab,kw OR (palsy):ti,ab,kw OR (palsies):ti,ab,kw OR (spasm):ti,ab,kw OR (spasms):ti,ab,kw OR (spastic paraparesis):ti,ab,kw OR (spastic parapareses):ti,ab,kw OR (motor function):ti,ab,kw OR (motor impairment):ti,ab,kw OR (motor disorder):ti,ab,kw OR (movement disorder):ti,ab,kw OR (movement impairment):ti,ab,kw OR (movement function):ti,ab,kw OR (motor recovery):ti,ab,kw OR (neurorehabilitation):ti,ab,kw OR (hemiparesis):ti,ab,kw OR (hemiplegia):ti,ab,kw OR (paresis):ti,ab,kw

#27 25 OR #26

#28 MeSH descriptor: [Randomized Controlled Trials as Topic] explode all trees

#29 MeSH descriptor: [Clinical Trials as Topic] explode all trees

#30 MeSH descriptor: [Random Allocation] explode all trees

#31 (clinical):ti,ab,kw OR (trial):ti,ab,kw OR (random*):ti,ab,kw

#32 #28 OR #29 OR #30 OR #31

#33 #18 AND #21 AND #24 AND #27 AND #32

**CNKI**

((SU =任务特异性训练 + 限制性运动疗法 + 上肢训练 + 周围电刺激 + 功能电刺激 + 神经肌肉电刺激 + 经皮电刺激 + 经颅直流电刺激 + 迷走神经刺激 + 经颅磁刺激 + 镜像运动疗法+ 虚拟现实疗法 + 生物反馈 + 机器人辅助疗法 + 针刺 + 运动想象训练) OR TKA = (任务特异性训练 + 限制性运动疗法 + 上肢训练 + 上臂训练 + 周围电刺激 + 功能电刺激 + 神经肌肉电刺激 + 经皮电刺激 + 经颅直流电刺激 + 迷走神经刺激 + 重复经颅磁刺激 + 镜像运动疗法 + 镜像疗法 + 虚拟现实暴露疗法 + 虚拟现实 + 生物反馈 + 机器人辅助运动治疗 + 机器人辅助 + 针灸 + 针刺 + 心理想象 + 运动想象 + 作业治疗 + 物理治疗 + 动作观察疗法 + 运动意象训练 + 非侵入性脑刺激 + 脑机接口 + 外骨骼 + 远程康复 + 增强现实)) AND (SU = (中风 + 卒中) OR TKA = (中风 + 卒中 + 脑卒中 + 缺血性中风 + 出血性中风 + 出血性卒中 + 缺血性卒中 + 脑梗死 + 脑梗塞 + 脑栓塞 + 脑出血 + 脑血管病 + 脑血管意外 + 卒中后遗症)) AND (SU = (上肢) OR TKA = (上肢 + 肩 + 肘 + 臂 + 腕 + 手 + 手指 + 掌)) AND (SU = (运动障碍) OR TKA = (运动障碍 + 运动功能 + 运动损伤 + 偏瘫 + 瘫痪 + 偏枯 + 半身不遂 **+** 轻瘫 + 运动恢复 + 神经康复)) AND (SU = (随机对照试验) OR TKA = (随机对照试验 + 随机对照研究 + 临床研究 + 临床试验 + RCT))

**Wanfang**

(题名或关键词:(任务特异性训练 OR 限制性运动疗法 OR 上肢训练 OR 上臂训练 OR 周围电刺激 OR 功能电刺激 OR 神经肌肉电刺激 OR 经皮电刺激 OR 经颅直流电刺激 OR 迷走神经刺激 OR经颅磁刺激 OR 重复经颅磁刺激 OR 镜像运动疗法 OR 镜像疗法 OR 虚拟现实暴露疗法 OR 虚拟现实 OR 生物反馈 OR 机器人辅助运动治疗 OR 机器人辅助 OR 针灸 OR 针刺 OR 心理想象 OR 运动想象 OR 作业治疗 OR 物理治疗 OR 动作观察疗法 OR 运动意象训练 OR 非侵入性脑刺激 OR 脑机接口 OR 外骨骼 OR 远程康复 OR 增强现实)) AND (题名或关键词:(中风 OR 卒中 OR 脑卒中 OR 缺血性中风 OR 出血性中风 OR 出血性卒中 OR 缺血性卒中 OR 脑梗死 OR 脑梗塞 OR 脑栓塞 OR 脑出血 OR 脑血管病 OR 脑血管意外 OR 卒中后遗症)) AND (题名或关键词:(上肢 OR 肩 OR 肘 OR 臂 OR 腕 OR 手 OR 手指 OR 掌)) AND (题名或关键词:(运动障碍 OR 运动功能 OR 运动损伤 OR 偏瘫 OR 瘫痪 OR 偏枯 OR 半身不遂OR轻瘫 OR 运动恢复 OR 神经康复)) AND (题名或关键词:(随机对照试验 OR 随机对照研究 OR 临床研究 OR 临床试验 OR RCT))

**VIP**

M=(任务特异性训练 OR 限制性运动疗法 OR 上肢训练 OR 上臂训练 OR 周围电刺激 OR 功能电刺激 OR 神经肌肉电刺激 OR 经皮电刺激 OR 经颅直流电刺激 OR 迷走神经刺激 OR经颅磁刺激 OR 重复经颅磁刺激 OR 镜像运动疗法 OR 镜像疗法 OR 虚拟现实暴露疗法 OR 虚拟现实 OR 生物反馈 OR 机器人辅助运动治疗 OR 机器人辅助 OR 针灸 OR 针刺 OR 心理想象 OR 运动想象 OR 作业治疗 OR 物理治疗 OR 动作观察疗法 OR 运动意象训练 OR 非侵入性脑刺激 OR 脑机接口 OR 外骨骼 OR 远程康复 OR 增强现实) AND M=(中风 OR 卒中 OR 脑卒中 OR 缺血性中风 OR 出血性中风 OR 出血性卒中 OR 缺血性卒中 OR 脑梗死 OR 脑梗塞 OR 脑栓塞 OR 脑出血 OR 脑血管病 OR 脑血管意外 OR 卒中后遗症) AND M=(上肢 OR 肩 OR 肘 OR 臂 OR 腕 OR 手 OR 手指 OR 掌) AND M=(运动障碍 OR 运动功能 OR 运动损伤 OR 偏瘫 OR 瘫痪 OR 偏枯 OR 半身不遂OR 轻瘫 OR 运动恢复 OR 神经康复) AND R=(随机对照试验 OR 随机对照研究 OR 临床研究 OR 临床试验 OR RCT)

**SinoMed**

#1 "任务特异性训练"[加权:扩展] OR "限制性运动疗法"[加权:扩展] OR "上肢训练"[加权:扩展] OR "周围电刺激"[加权:扩展] OR "功能电刺激"[加权:扩展] OR "神经肌肉电刺激"[加权:扩展] OR "经皮电刺激"[加权:扩展] OR "经颅直流电刺激"[加权:扩展] OR "迷走神经刺激"[加权:扩展] OR "经颅磁刺激"[加权:扩展] OR "镜像运动疗法"[加权:扩展] OR "虚拟现实疗法"[加权:扩展] OR "生物反馈"[加权:扩展] OR "机器人辅助疗法"[加权:扩展] OR "针刺"[加权:扩展] OR "运动想象训练"[加权:扩展]

#2 "任务特异性训练"[常用字段:智能] OR "限制性运动疗法"[常用字段:智能] OR "上肢训练"[常用字段:智能] OR "上臂训练"[常用字段:智能] OR "周围电刺激"[常用字段:智能] OR "功能电刺激"[常用字段:智能] OR "神经肌肉电刺激"[常用字段:智能] OR "经皮电刺激"[常用字段:智能] OR "经颅直流电刺激"[常用字段:智能] OR "迷走神经刺激"[常用字段:智能] OR "重复经颅磁刺激"[常用字段:智能] OR "镜像运动疗法"[常用字段:智能] OR "镜像疗法"[常用字段:智能] OR "虚拟现实暴露疗法"[常用字段:智能] OR "虚拟现实"[常用字段:智能] OR "生物反馈"[常用字段:智能] OR "机器人辅助运动治疗"[常用字段:智能] OR "机器人辅助"[常用字段:智能] OR "针灸"[常用字段:智能] OR "针刺"[常用字段:智能] OR "心理想象"[常用字段:智能] OR "运动想象"[常用字段:智能] OR "作业治疗"[常用字段:智能] OR "物理治疗"[常用字段:智能] OR "动作观察疗法"[常用字段:智能] OR "运动意象训练"[常用字段:智能] OR "非侵入性脑刺激"[常用字段:智能] OR "脑机接口"[常用字段:智能] OR "外骨骼"[常用字段:智能] OR "远程康复"[常用字段:智能] OR "增强现实"[常用字段:智能]

#3 (#2) OR (#1)

#4 "中风" [加权:扩展] OR "卒中" [加权:扩展]

#5 "中风"[常用字段:智能] OR "卒中"[常用字段:智能] OR "脑卒中"[常用字段:智能] OR "缺血性中风"[常用字段:智能] OR "出血性中风"[常用字段:智能] OR "出血性卒中"[常用字段:智能] OR "缺血性卒中"[常用字段:智能] OR "脑梗死"[常用字段:智能] OR "脑梗塞"[常用字段:智能] OR "脑栓塞"[常用字段:智能] OR "脑出血"[常用字段:智能] OR "脑血管病"[常用字段:智能] OR "脑血管意外"[常用字段:智能] OR "卒中后遗症"[常用字段:智能]

#6 (#5) OR (#4)

#7 "上肢" [加权:扩展]

#8 "上肢"[常用字段:智能] OR "肩"[常用字段:智能] OR "肘"[常用字段:智能] OR "臂"[常用字段:智能] OR "腕"[常用字段:智能] OR "手"[常用字段:智能] OR "手指"[常用字段:智能] OR "掌"[常用字段:智能]

#9 (#8) OR (#7)

#10 "运动障碍" [加权:扩展]

#11 "运动障碍"[常用字段:智能] OR "运动功能"[常用字段:智能] OR "运动损伤"[常用字段:智能] OR "偏瘫"[常用字段:智能] OR "瘫痪"[常用字段:智能] OR "偏枯"[常用字段:智能] OR "半身不遂"[常用字段:智能] OR "轻瘫"[常用字段:智能] OR "运动恢复"[常用字段:智能] OR "神经康复"[常用字段:智能]

#12 (#11) OR (#10)

#13 "随机对照试验"[不加权:扩展]

#14 "随机对照试验"[常用字段:智能] OR "随机"[常用字段:智能] OR "对照"[常用字段:智能] OR "随机对照研究"[常用字段:智能] OR "临床研究"[常用字段:智能] OR "临床试验"[常用字段:智能] OR "RCT "[常用字段:智能]

#15 (#14) OR (#13)

#16 (#15) AND (#12) AND (#9) AND (#6) AND (#3)

**Figure S1. Forest plot of FMA-UE**

**(A)** **The control group was CON**


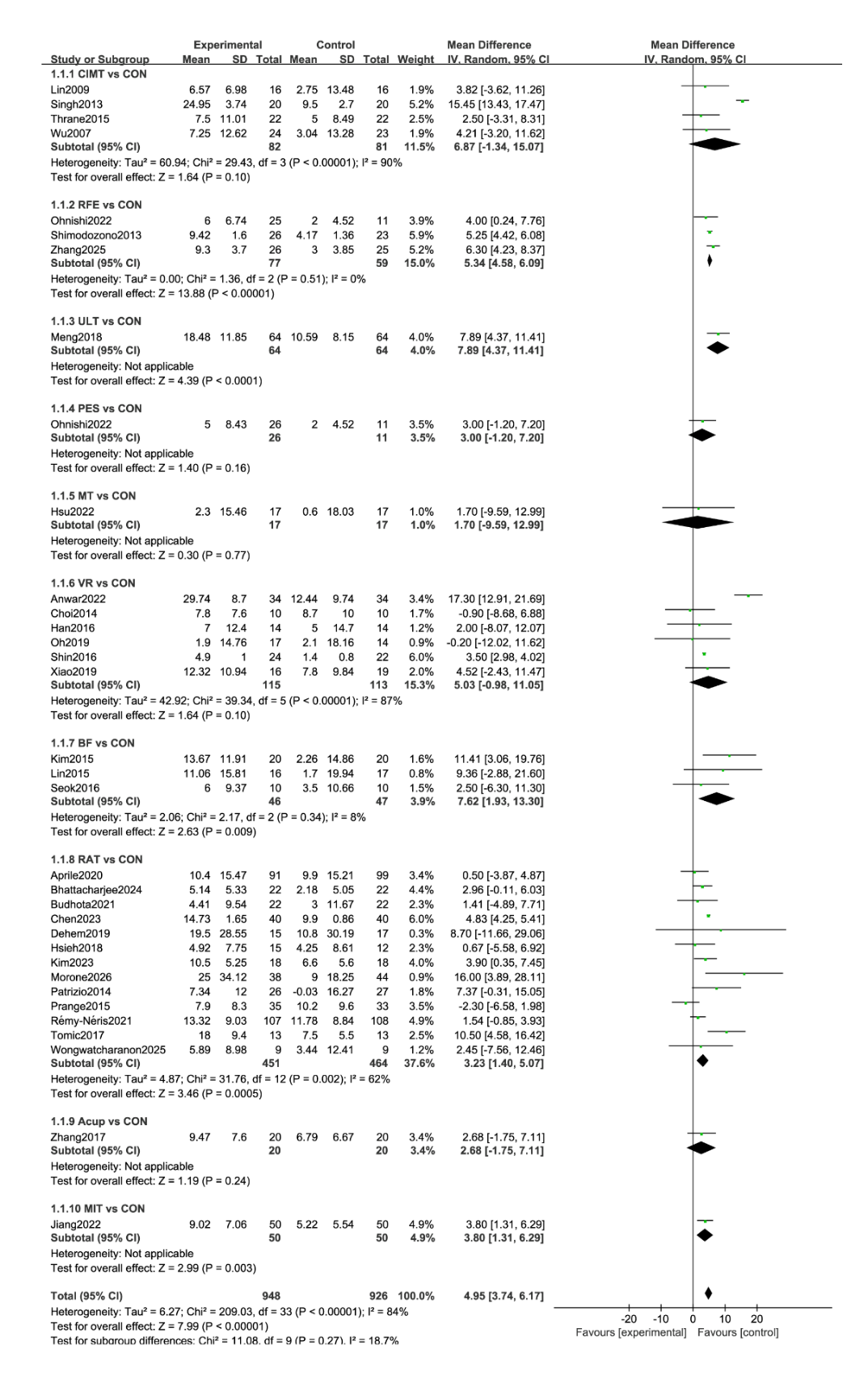


**(B) The control group was sham**


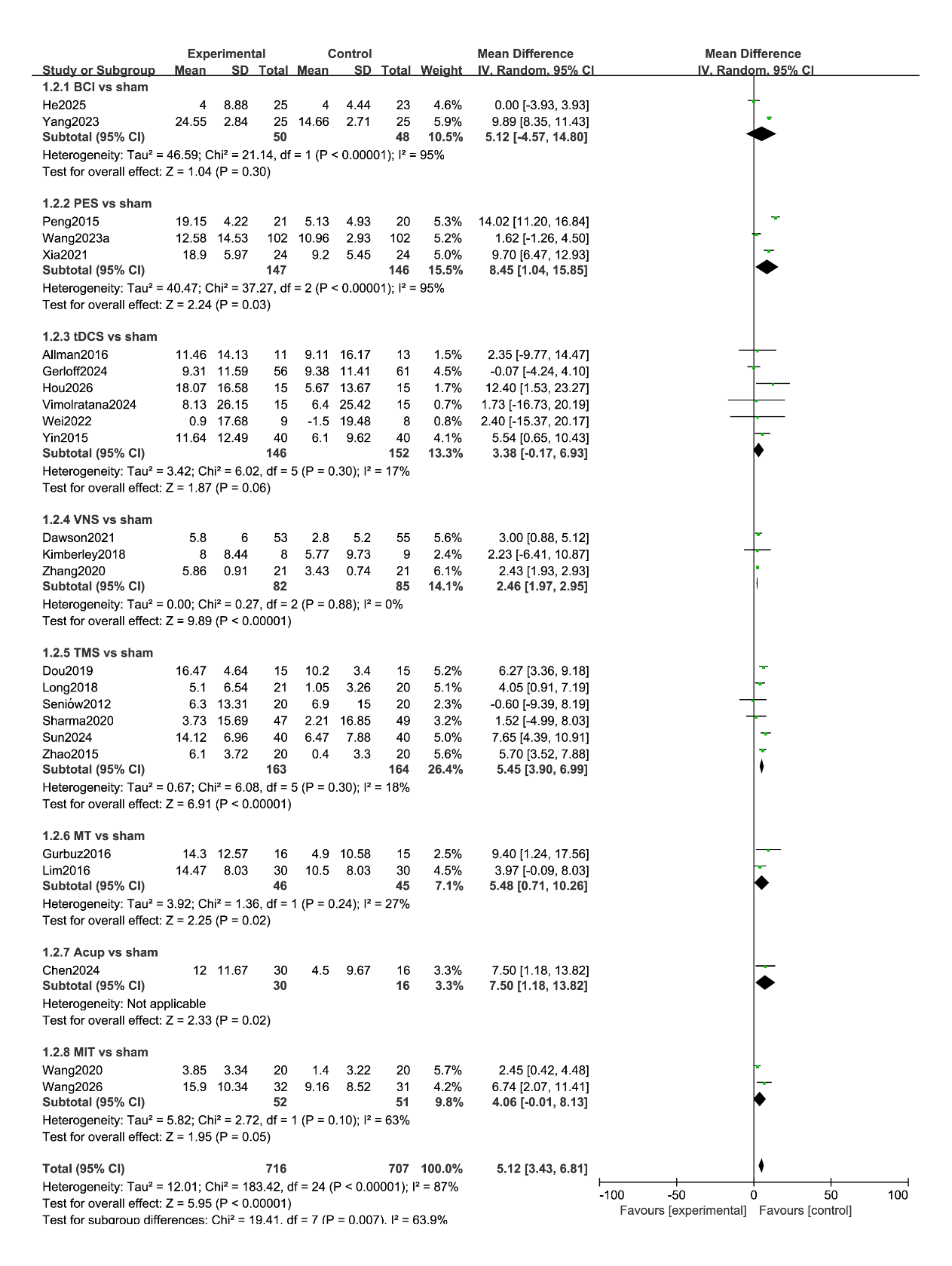


**Figure S2. Forest plot of ADL**

**(A)** **The control group was CON**


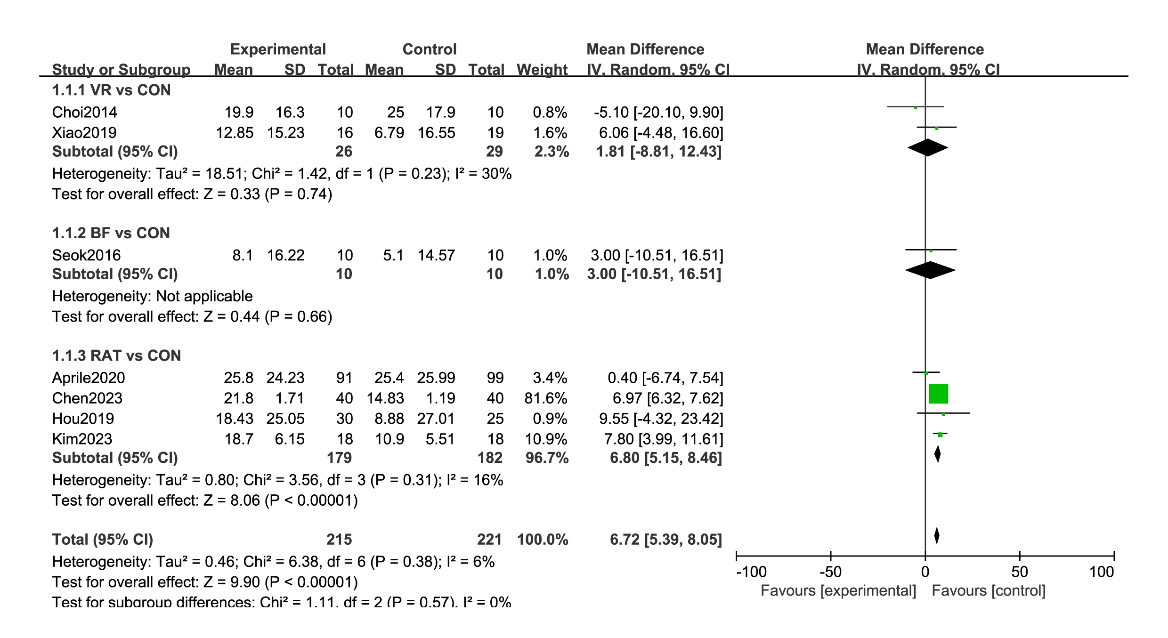


**(B) The control group was sham**
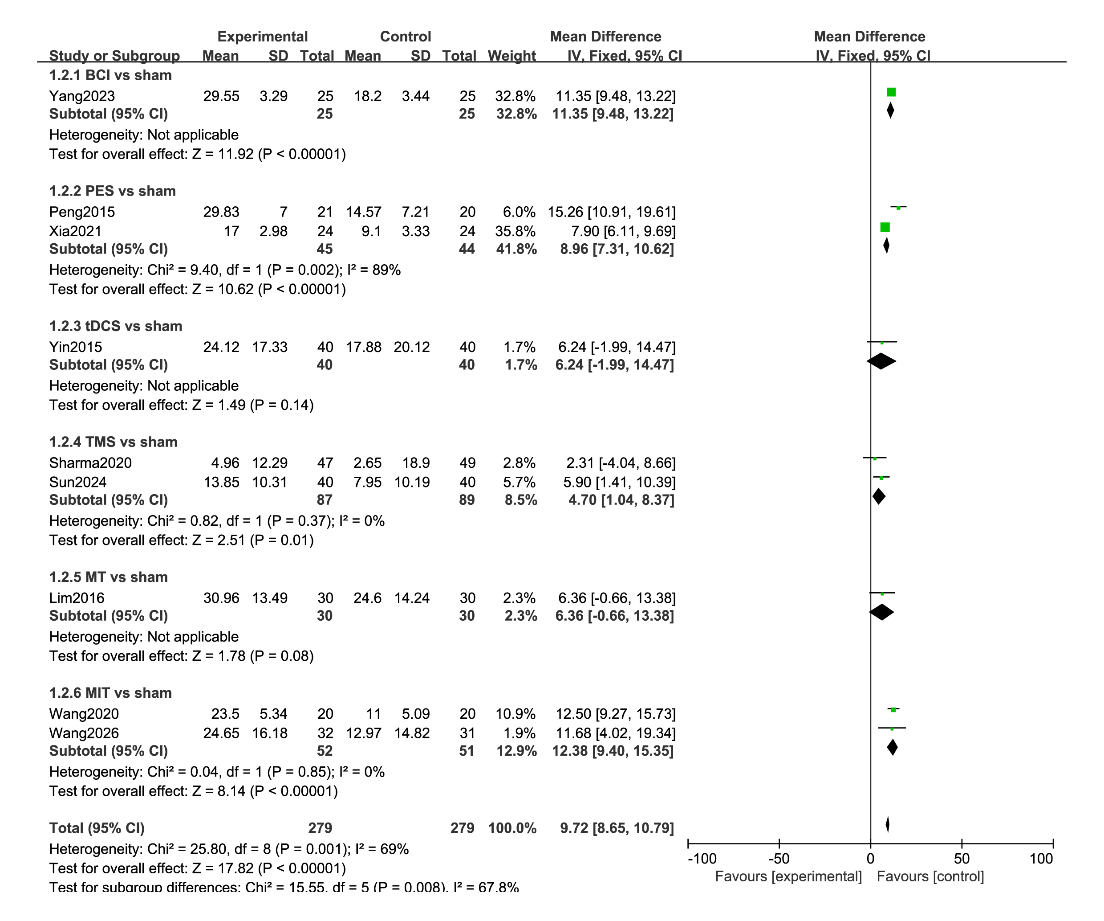


**Figure S3. Forest plot of ARAT**

**(A)** **The control group was CON**


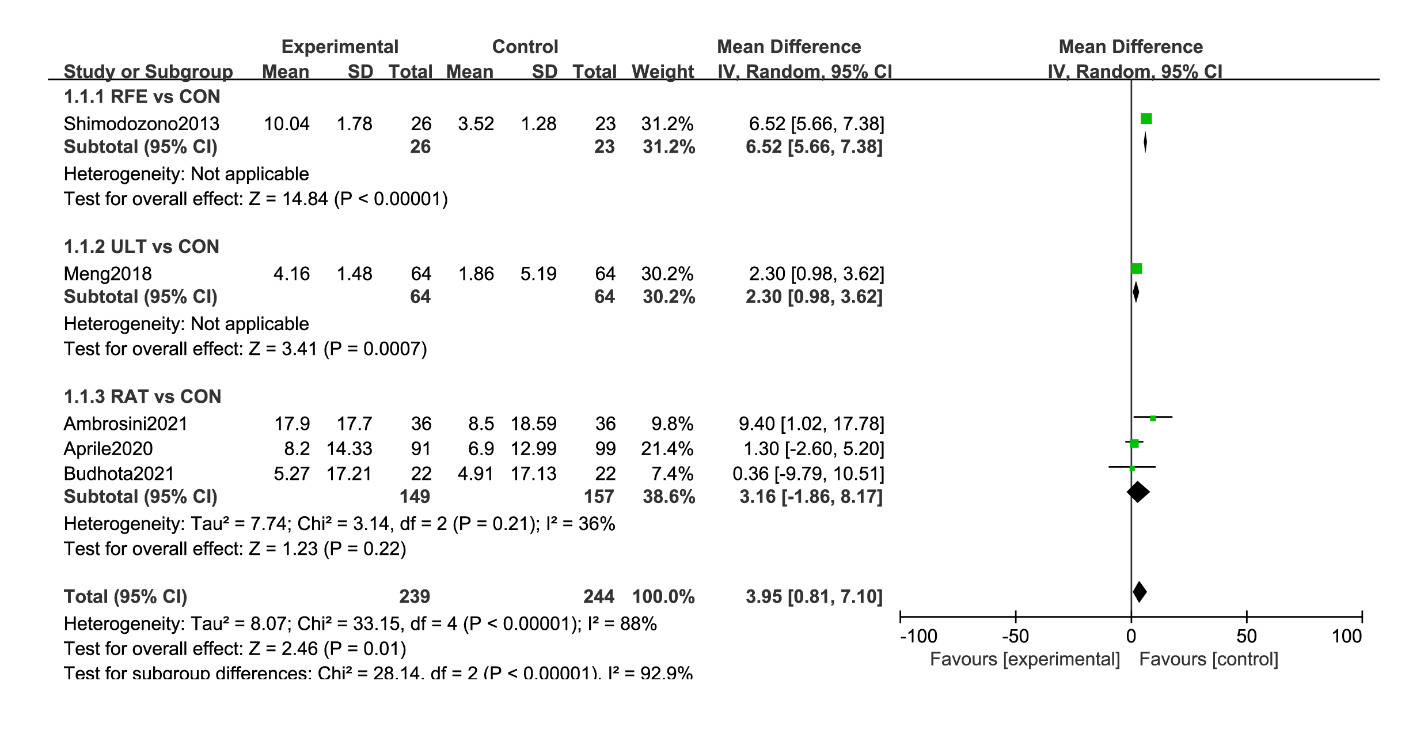


**(B) The control group was sham**


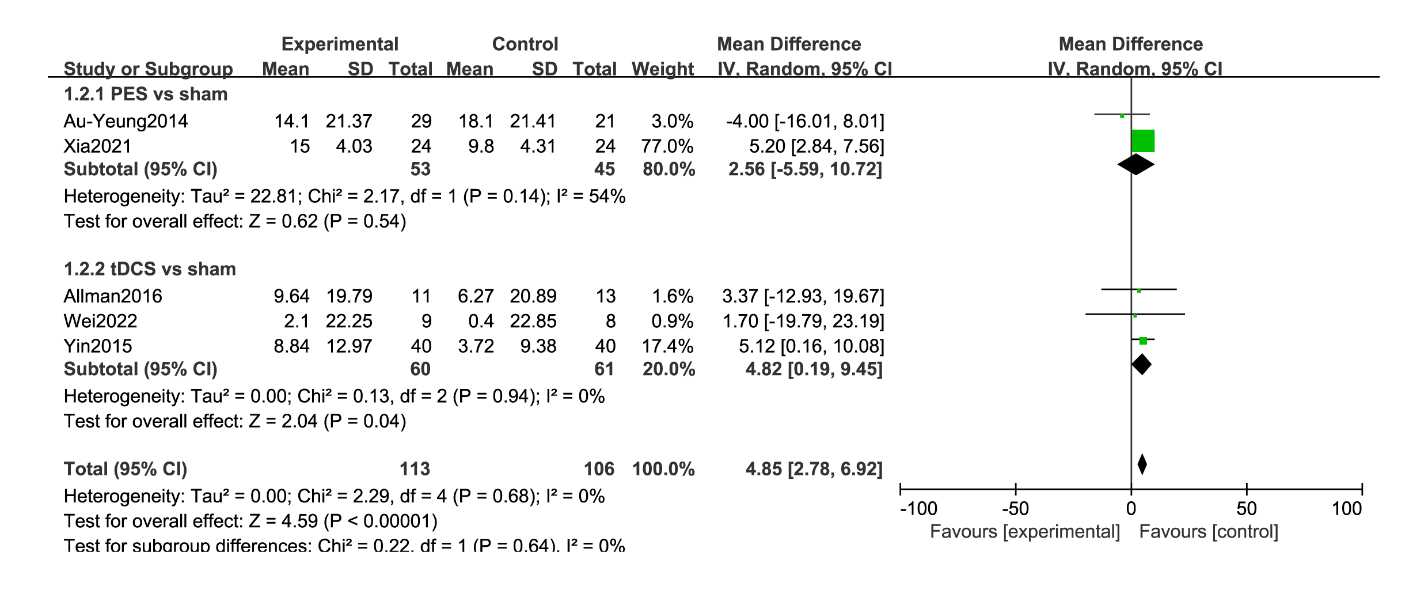


**Figure S4. Forest plot of WMFT**

**(A)** **The control group was CON**


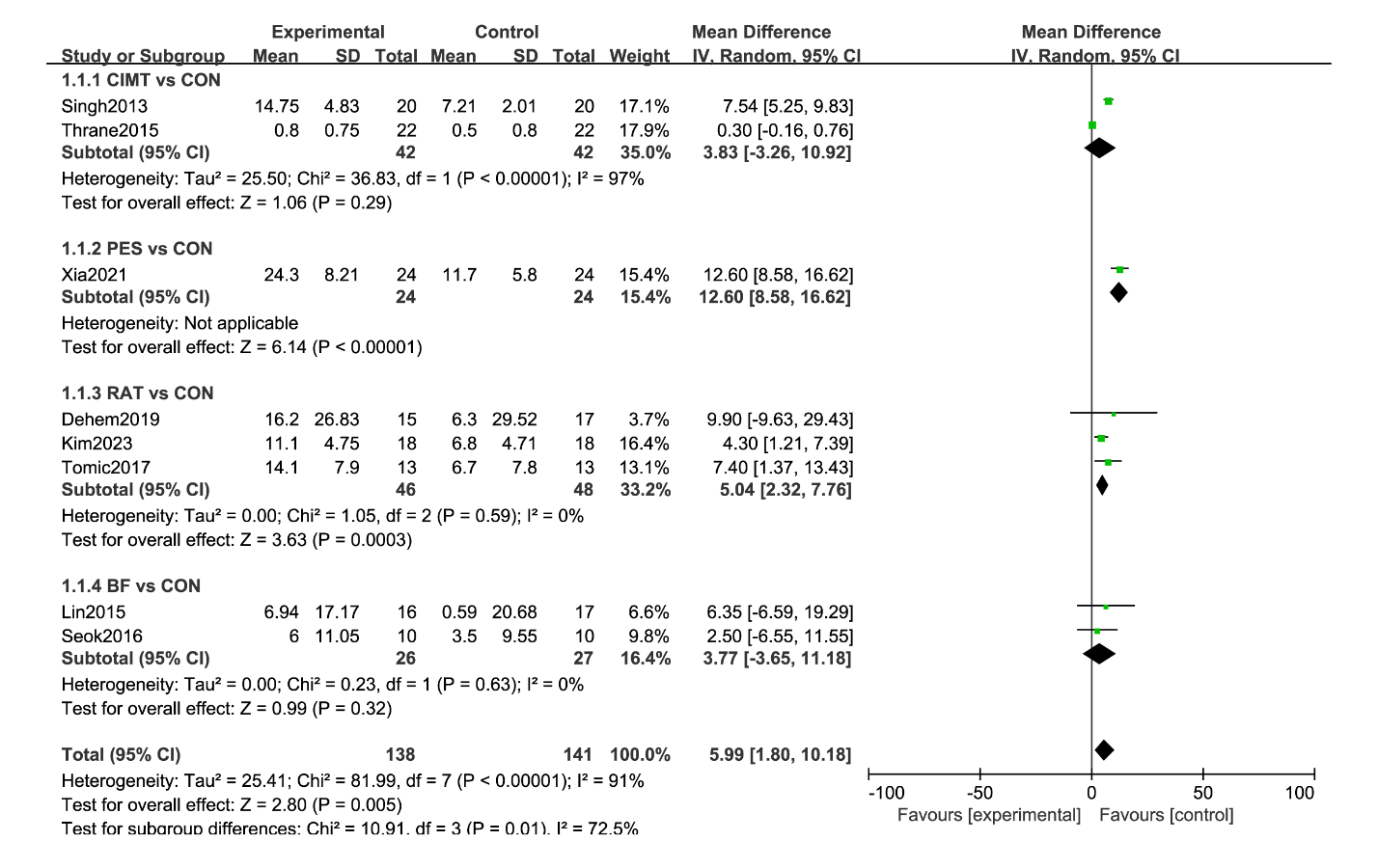


**(B) The control group was sham**


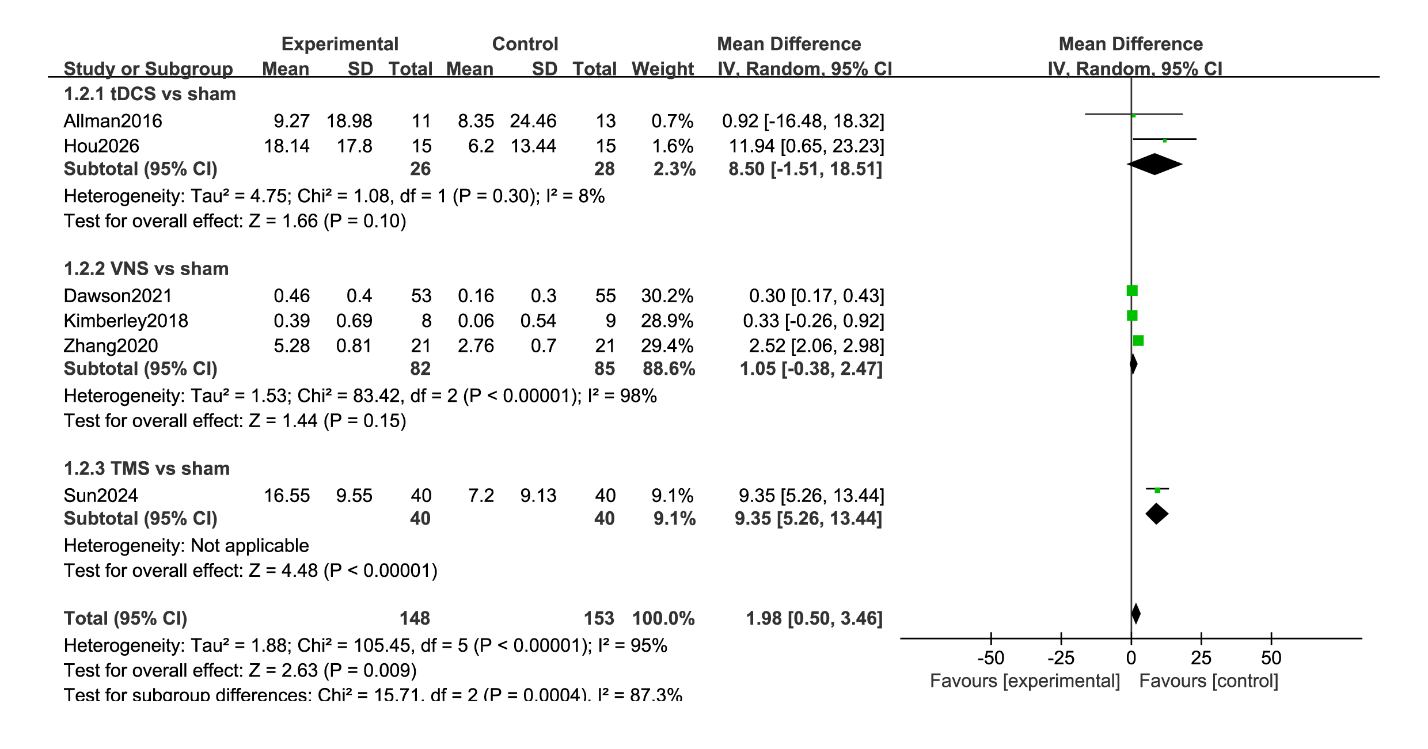


**Figure S5. Forest plot of BBT**

**(A)** **The control group was CON**


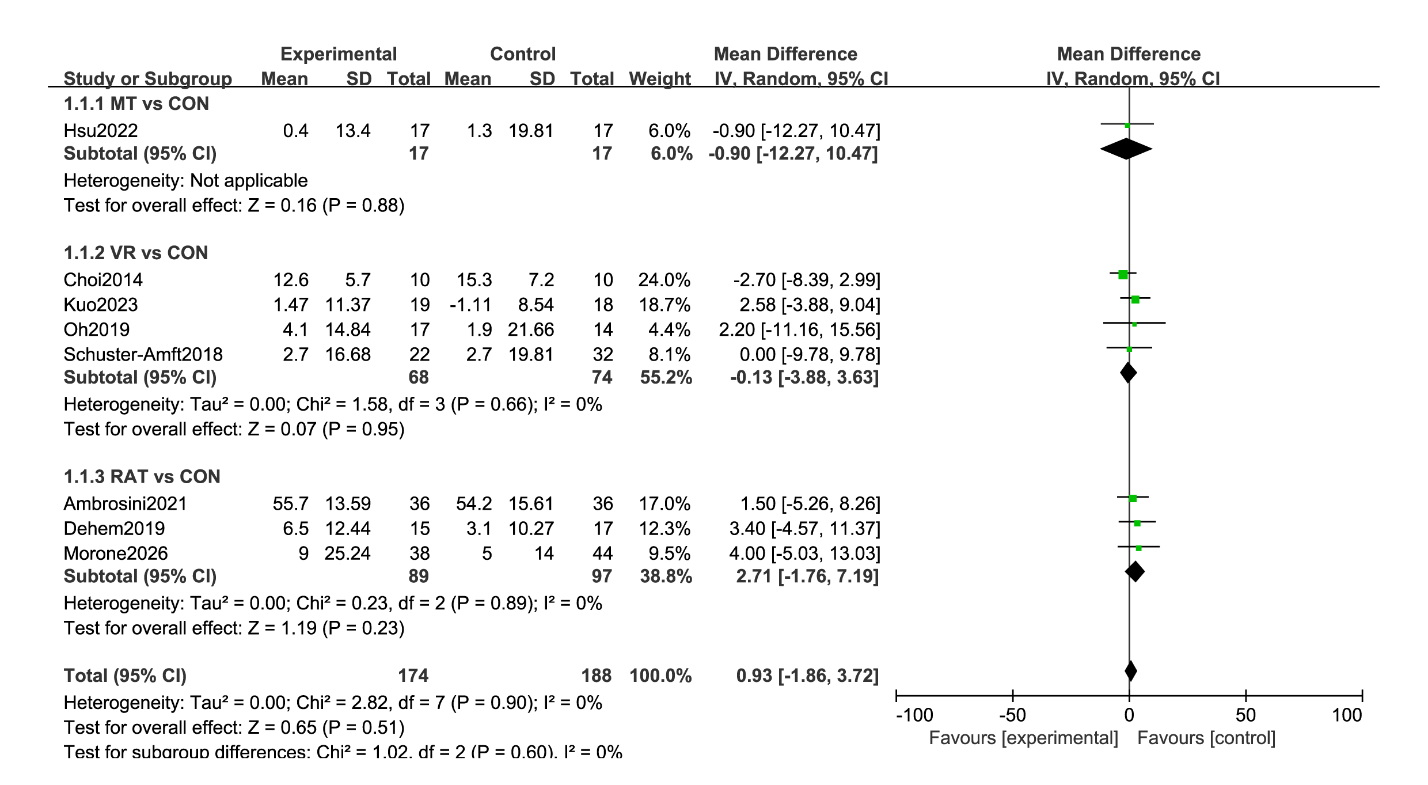


**(B) The control group was sham**


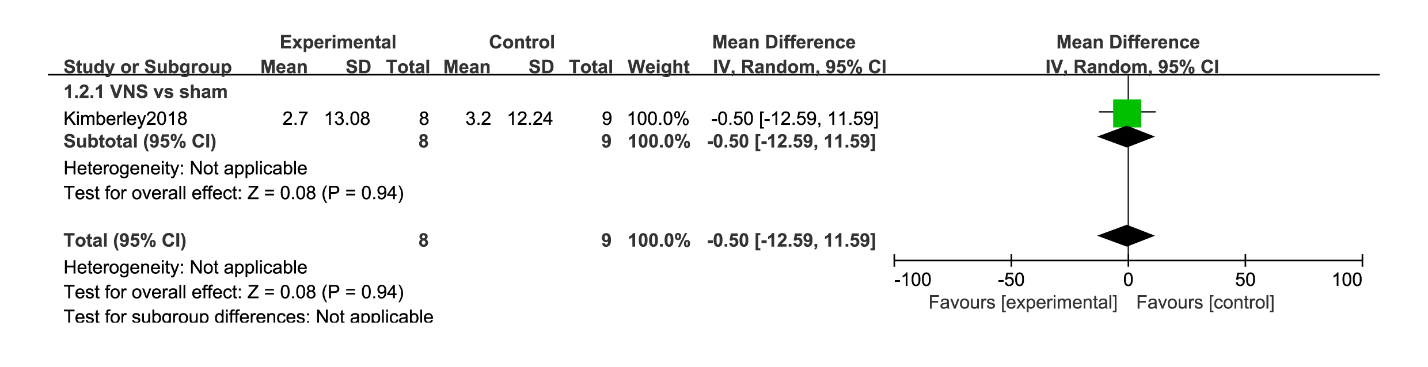


**Figure S6. Forest plot of GS**

**(A)The control group was CON**


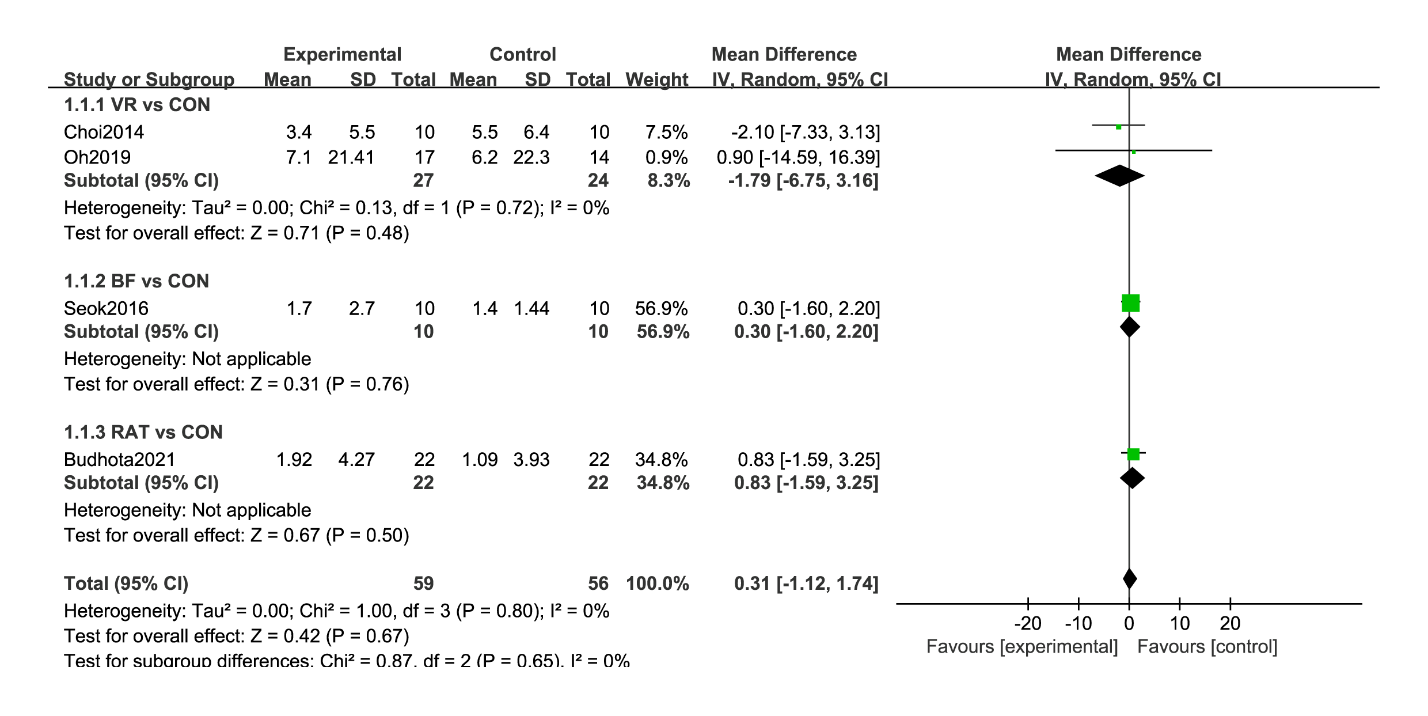


**(B)The control group was sham**


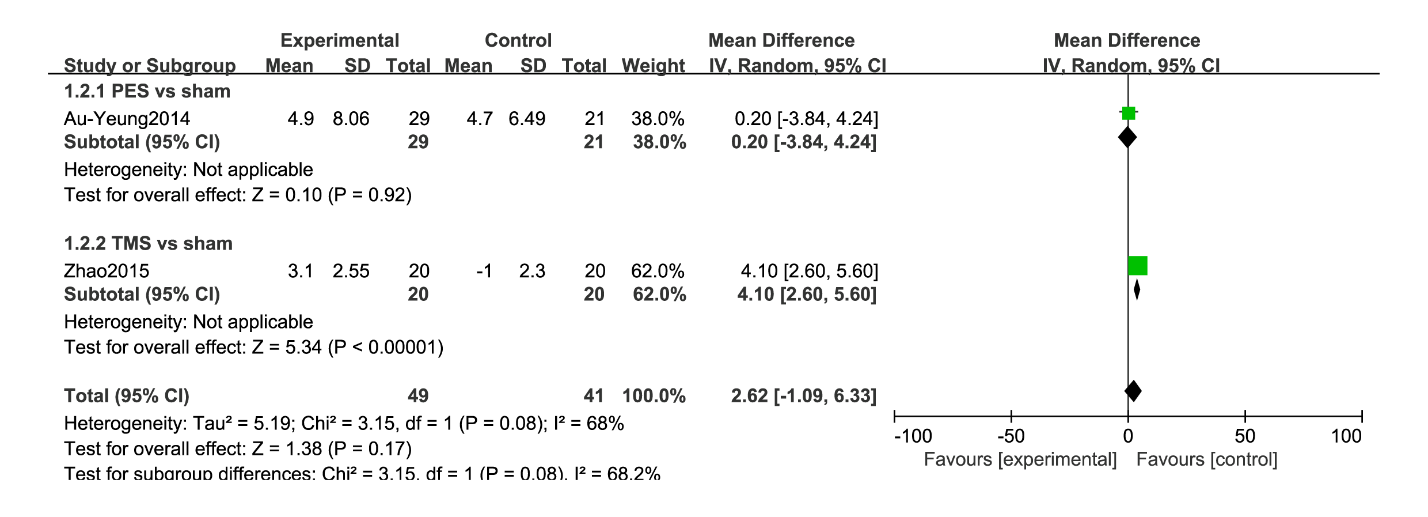


**Figure S7. Forest plot of MAS**

**(A)The control group was CON**


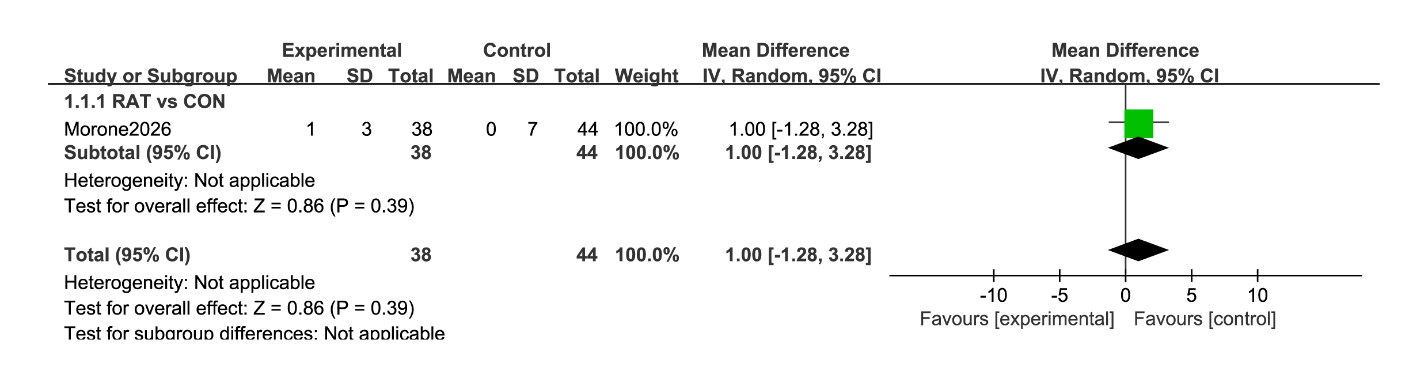


**(B) The control group was sham**


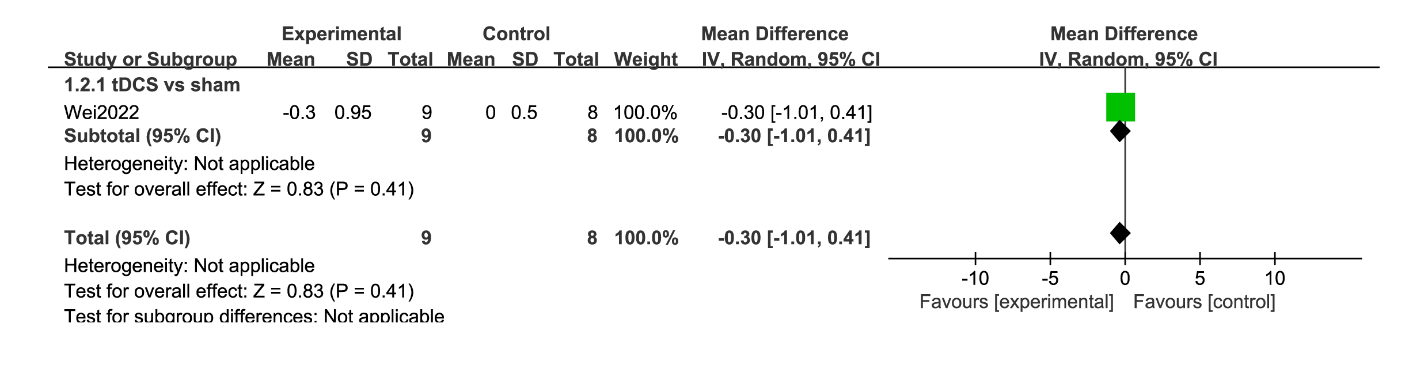


**Figure S8. Network Meta-analysis Results for Subgroup Analysis by Disease Stage**

1. **Ranking Probability Plot of Subacute**

**
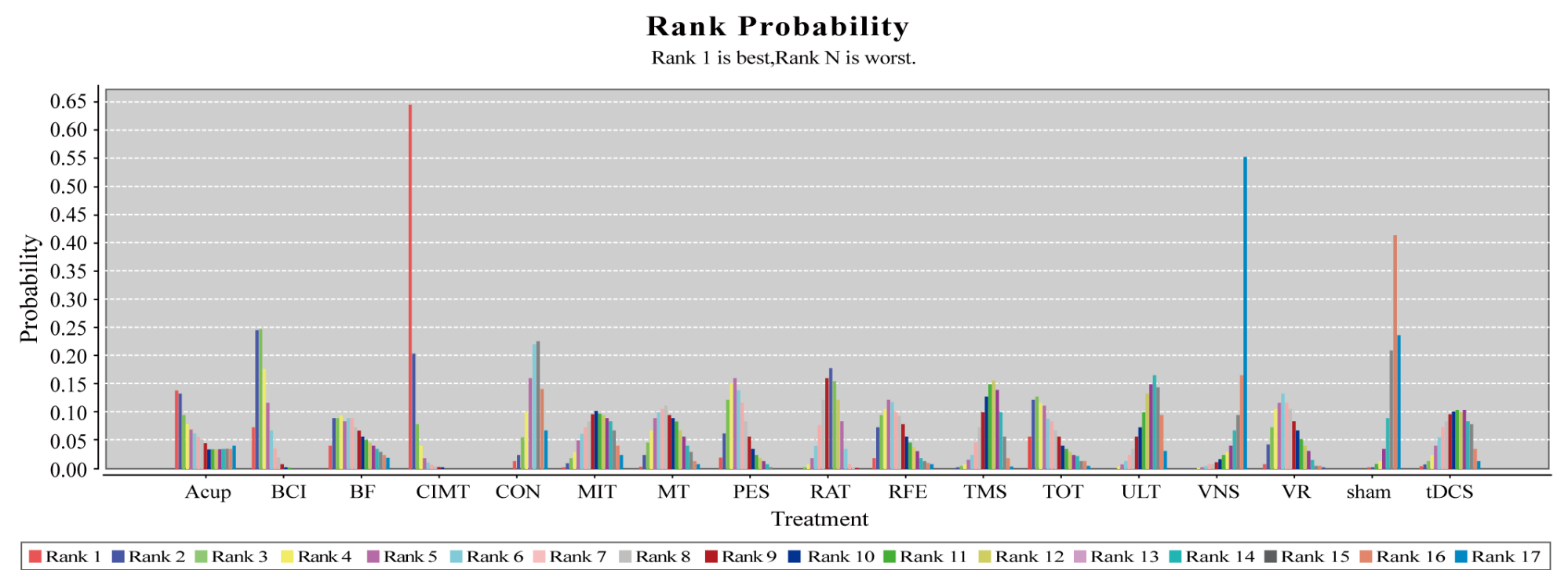
**

1. **League Table of Subacute**
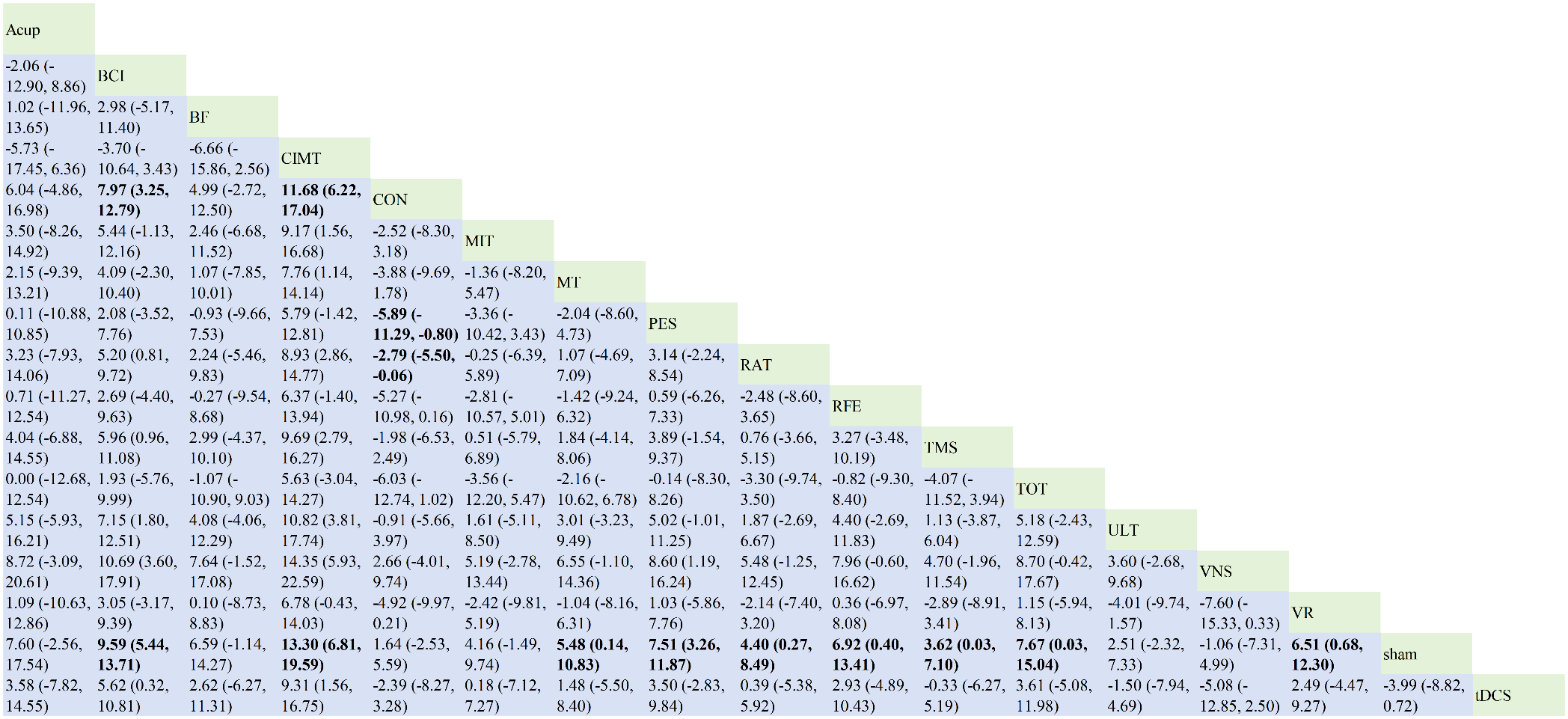


Note: The results are reported based on the lower triangular matrix of the ADDIS league table. Therefore, effect estimates are expressed as column-defining treatments versus row-defining treatments (column vs row). Bold values indicate statistically significant differences.

**Figure S9. Network Meta-analysis Results for Subgroup Analysis by Disease Severity**

**(A)** **Ranking Probability Plot of Moderate**


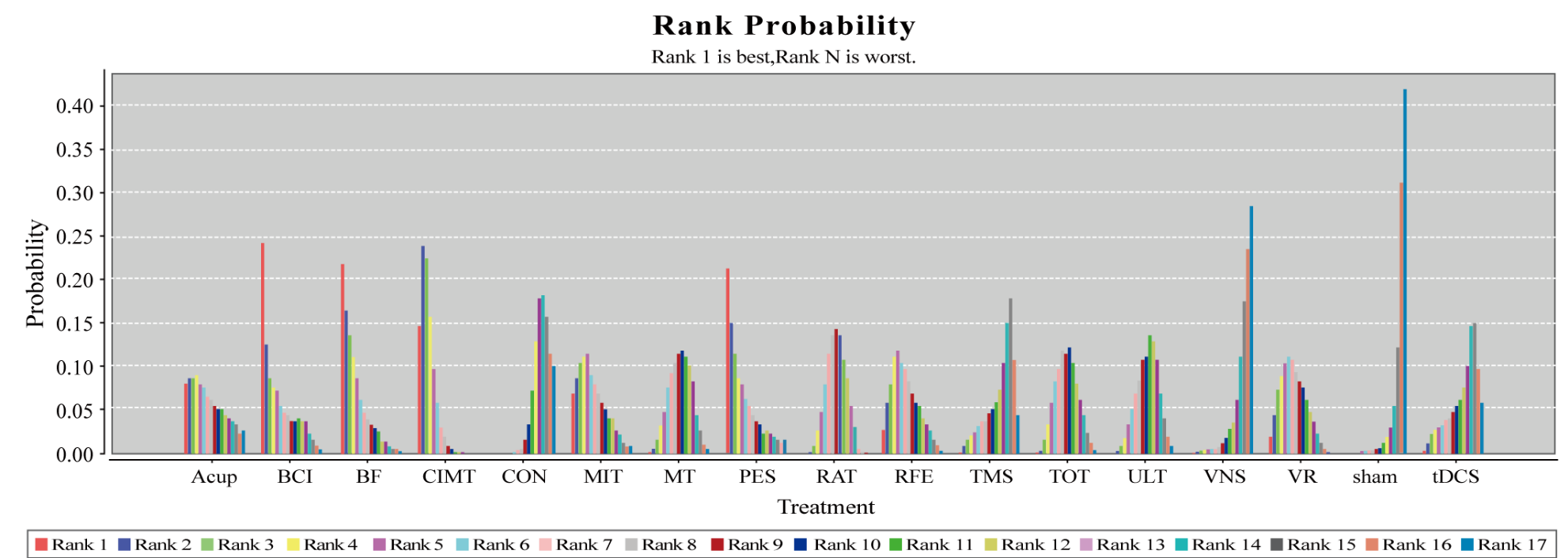


**(B) League Table of Moderate**

**
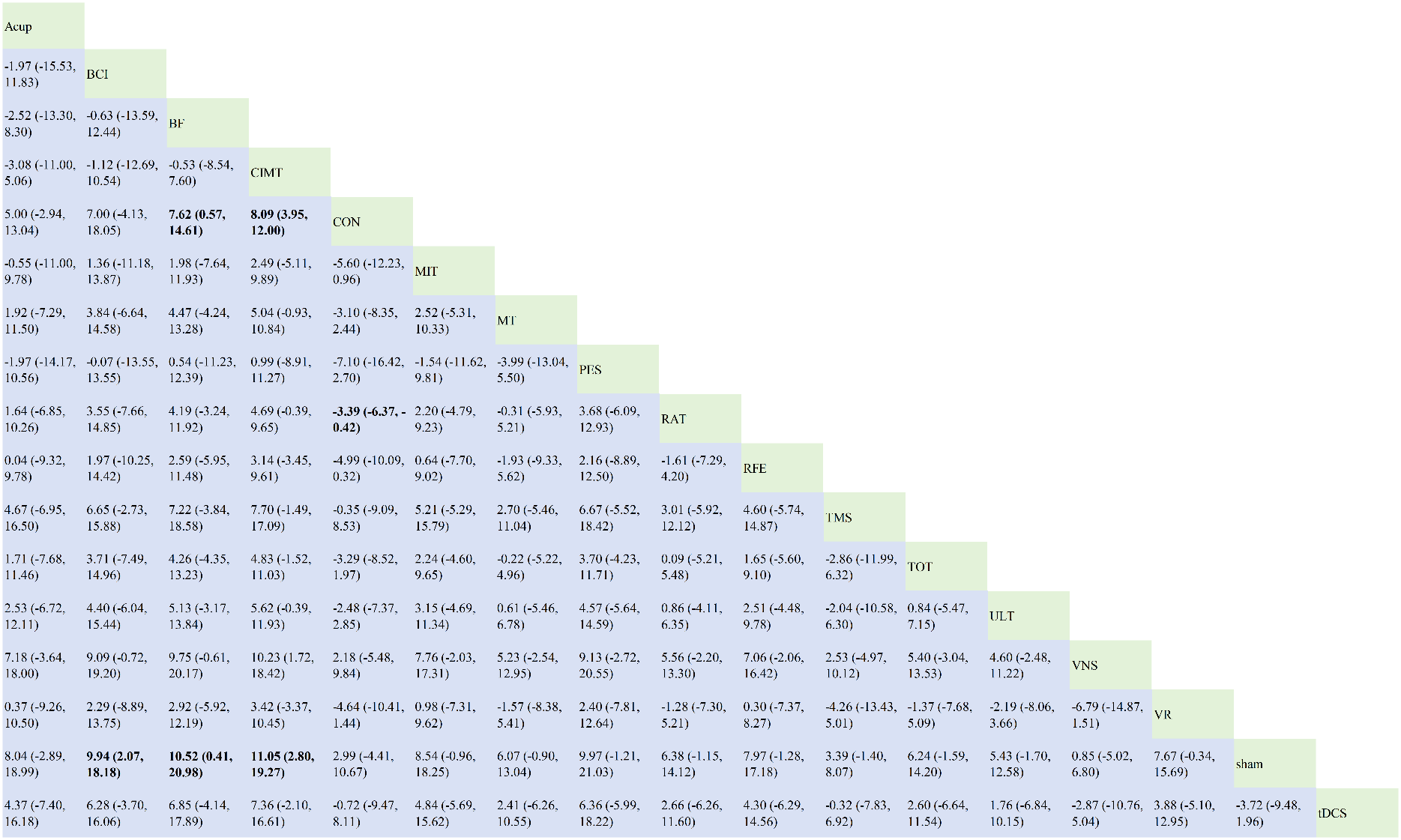
**

Note: The results are reported based on the lower triangular matrix of the ADDIS league table. Therefore, effect estimates are expressed as column-defining treatments versus row-defining treatments (column vs row). Bold values indicate statistically significant differences.

**(C)Ranking Probability Plot of Severe**


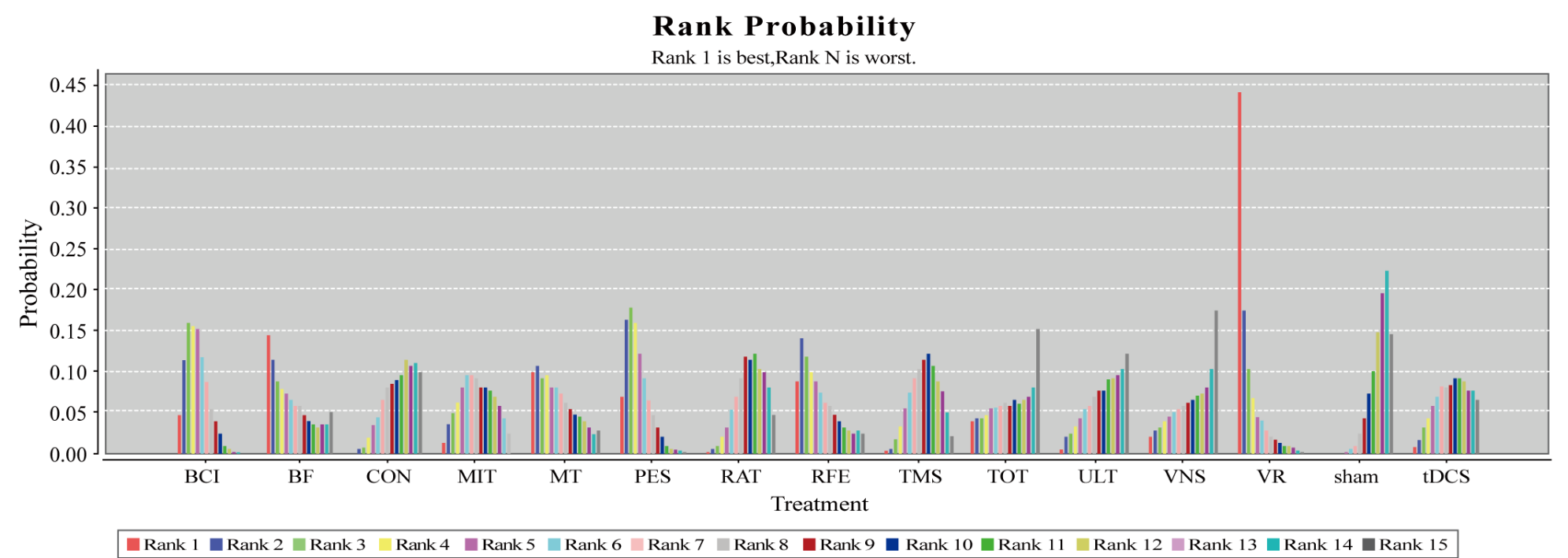


**(D)League Table of Severe**

**
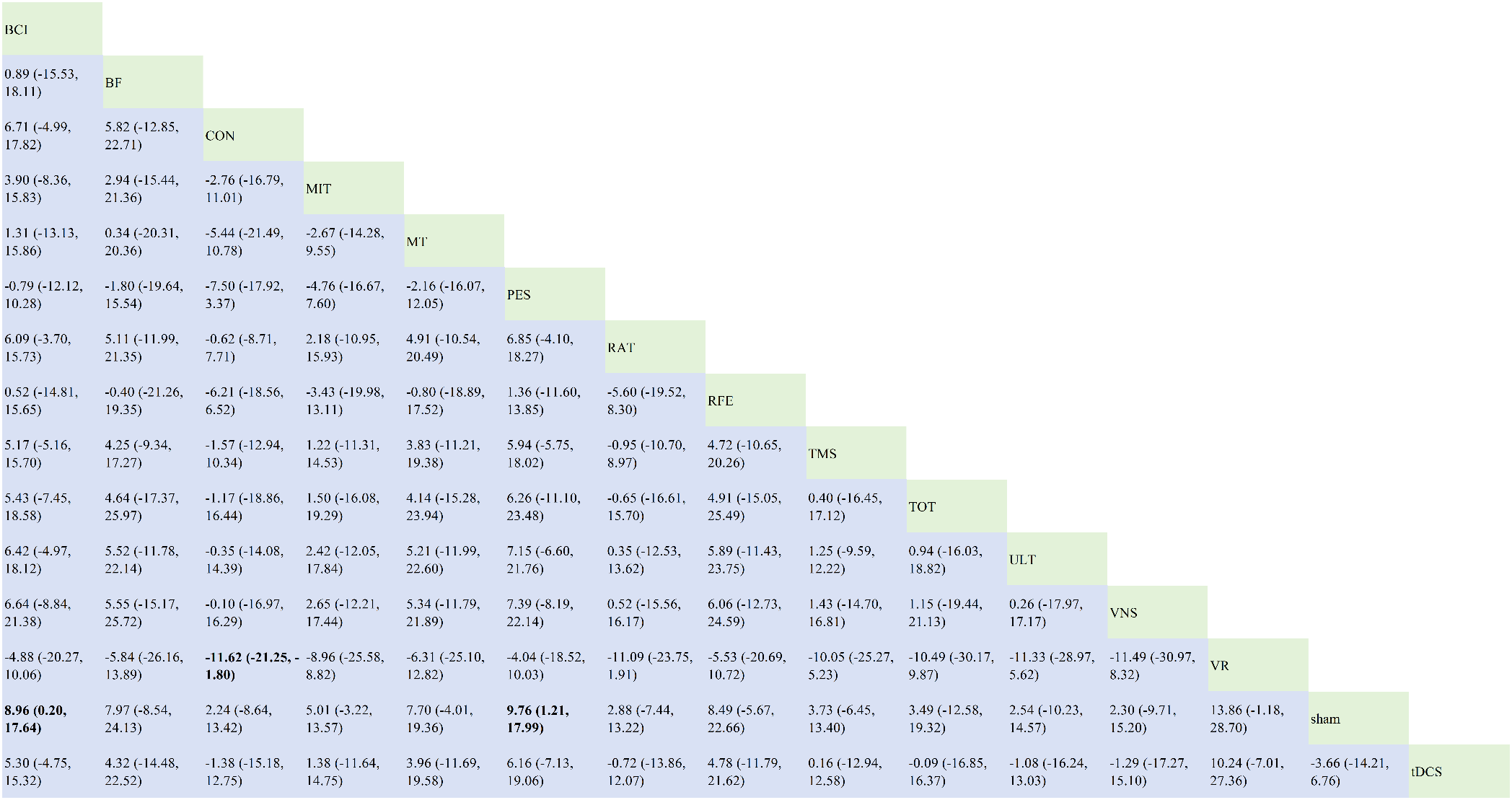
**

Note: The results are reported based on the lower triangular matrix of the ADDIS league table. Therefore, effect estimates are expressed as column-defining treatments versus row-defining treatments (column vs row). Bold values indicate statistically significant differences.

**Figure S10. Sensitivity analysis restricted to sham-controlled studies**

**(A)** **Ranking Probability Plot**


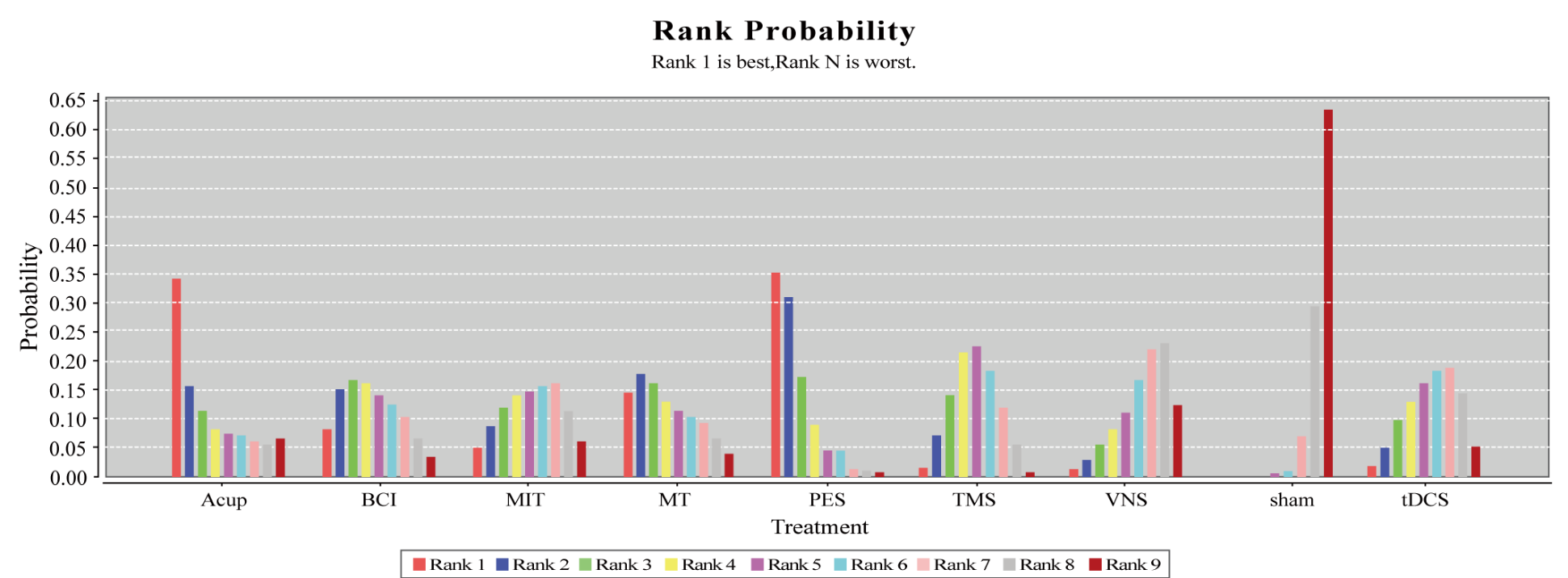


**(B) League Table**

**
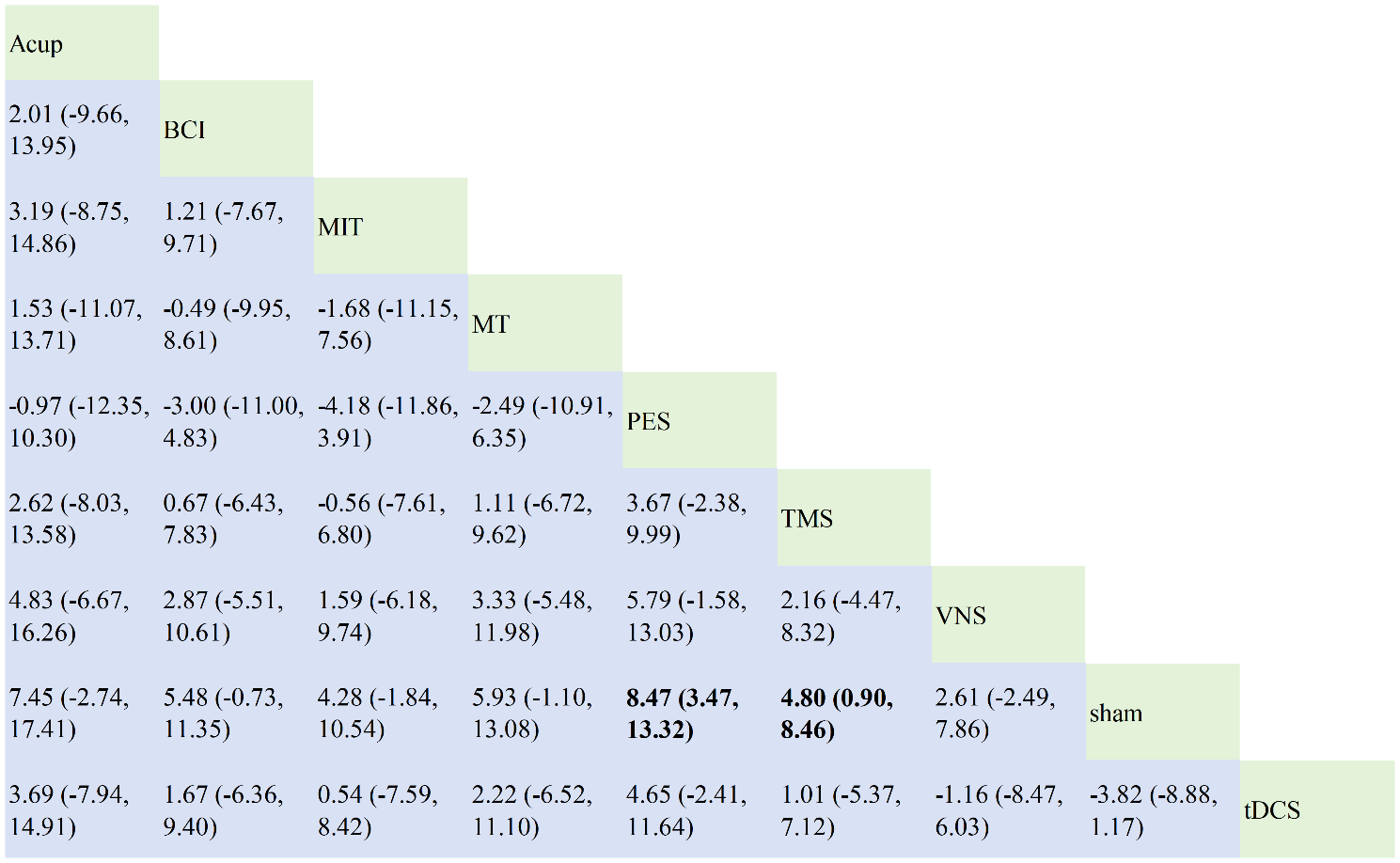
**

Note: The results are reported based on the lower triangular matrix of the ADDIS league table. Therefore, effect estimates are expressed as column-defining treatments versus row-defining treatments (column vs row). Bold values indicate statistically significant differences.

**Figure S11. Sensitivity Analysis Excluding Studies with High Risk of Bias**

**(A)** **Ranking Probability Plot**


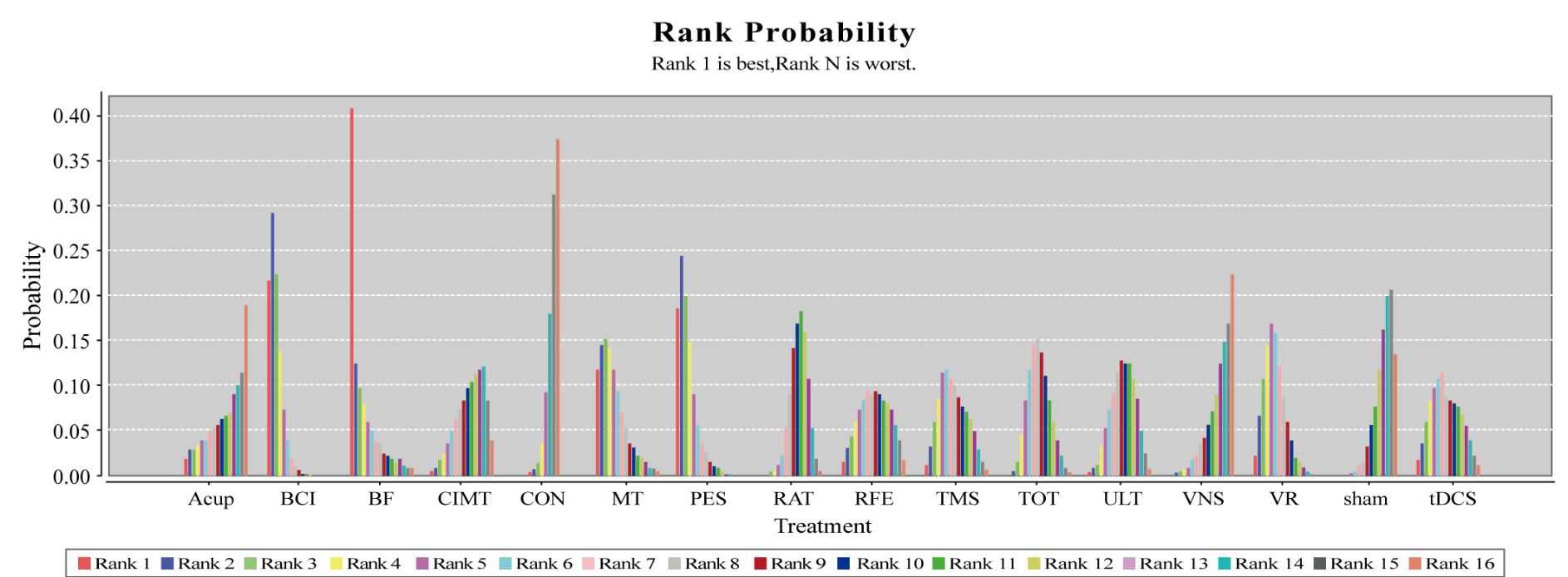


**(B) League Table**

**
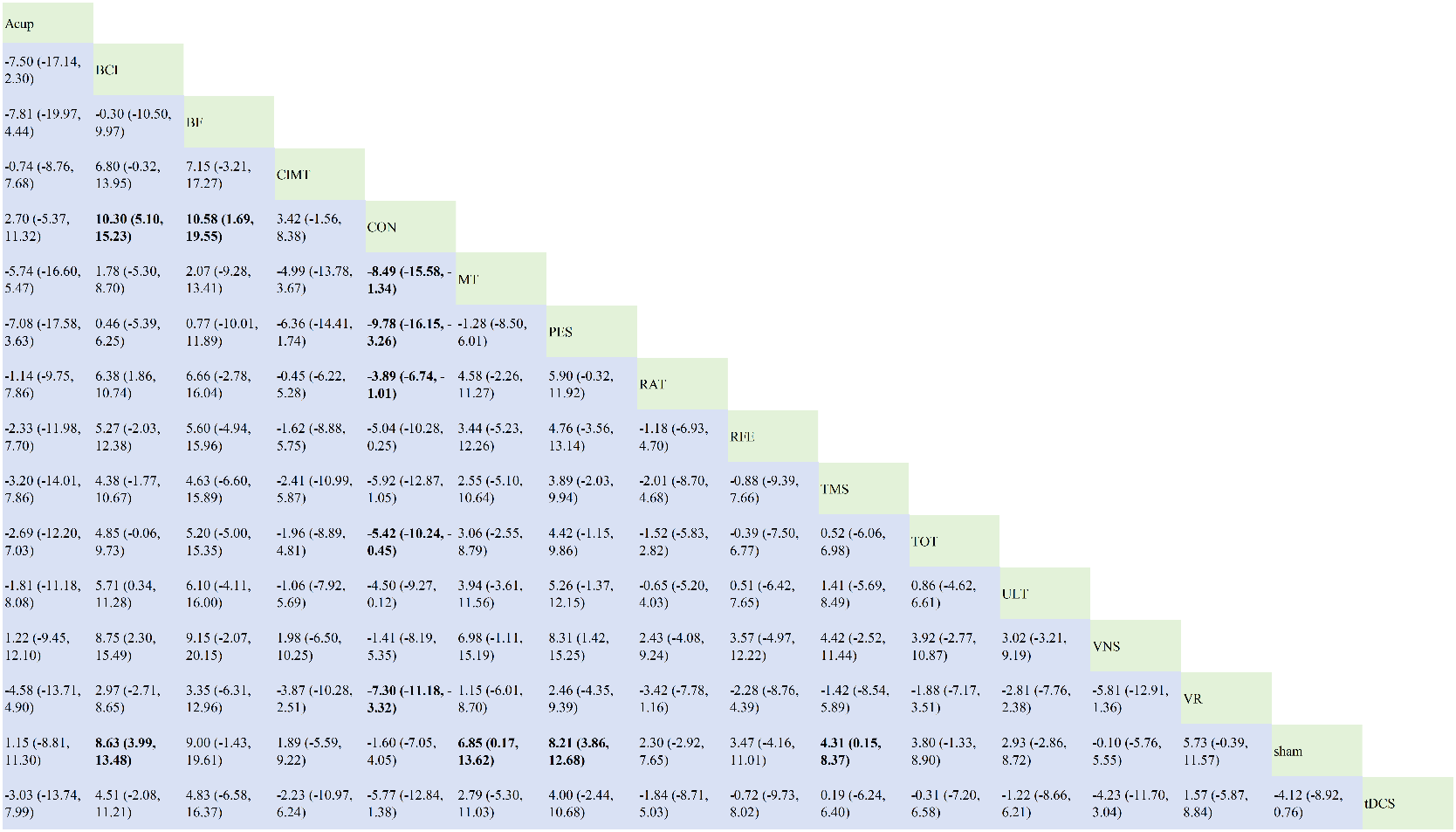
**

Note: The results are reported based on the lower triangular matrix of the ADDIS league table. Therefore, effect estimates are expressed as column-defining treatments versus row-defining treatments (column vs row). Bold values indicate statistically significant differences.

**Figure S12. Sensitivity Analysis Excluding Studies Including Recurrent Stroke Patients**

**(A)** **Ranking Probability Plot**


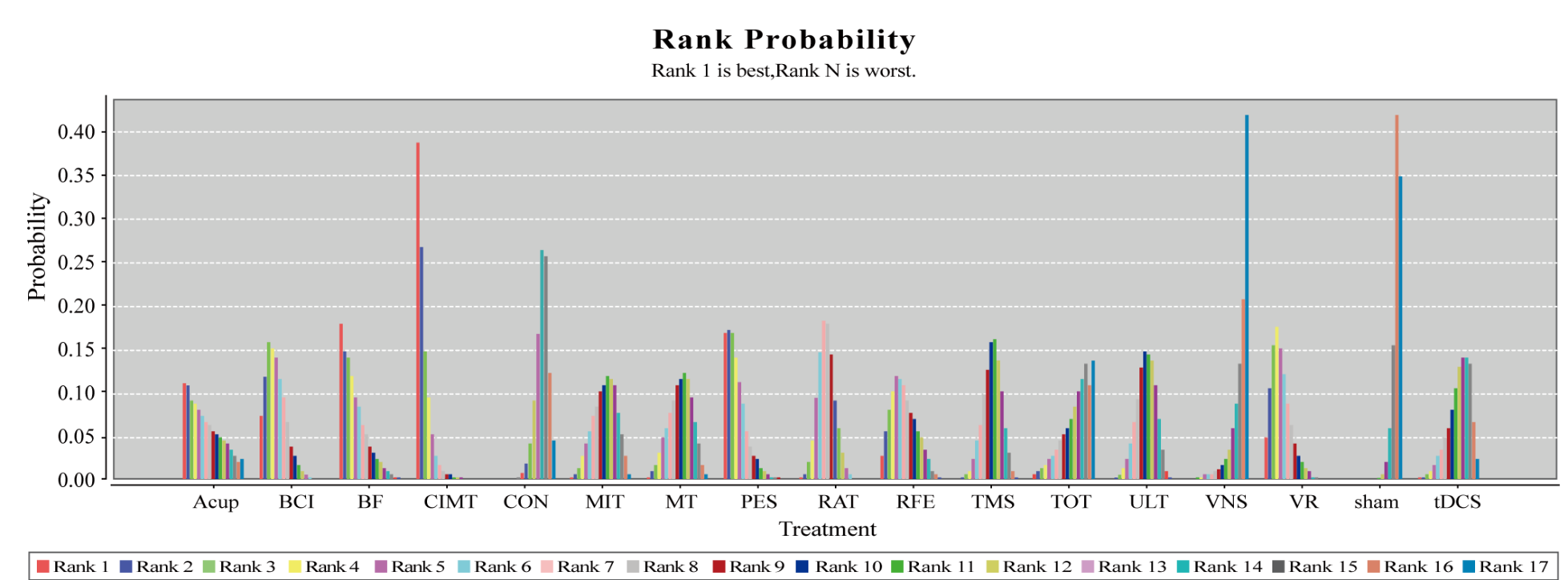


**(B) League Table**

**
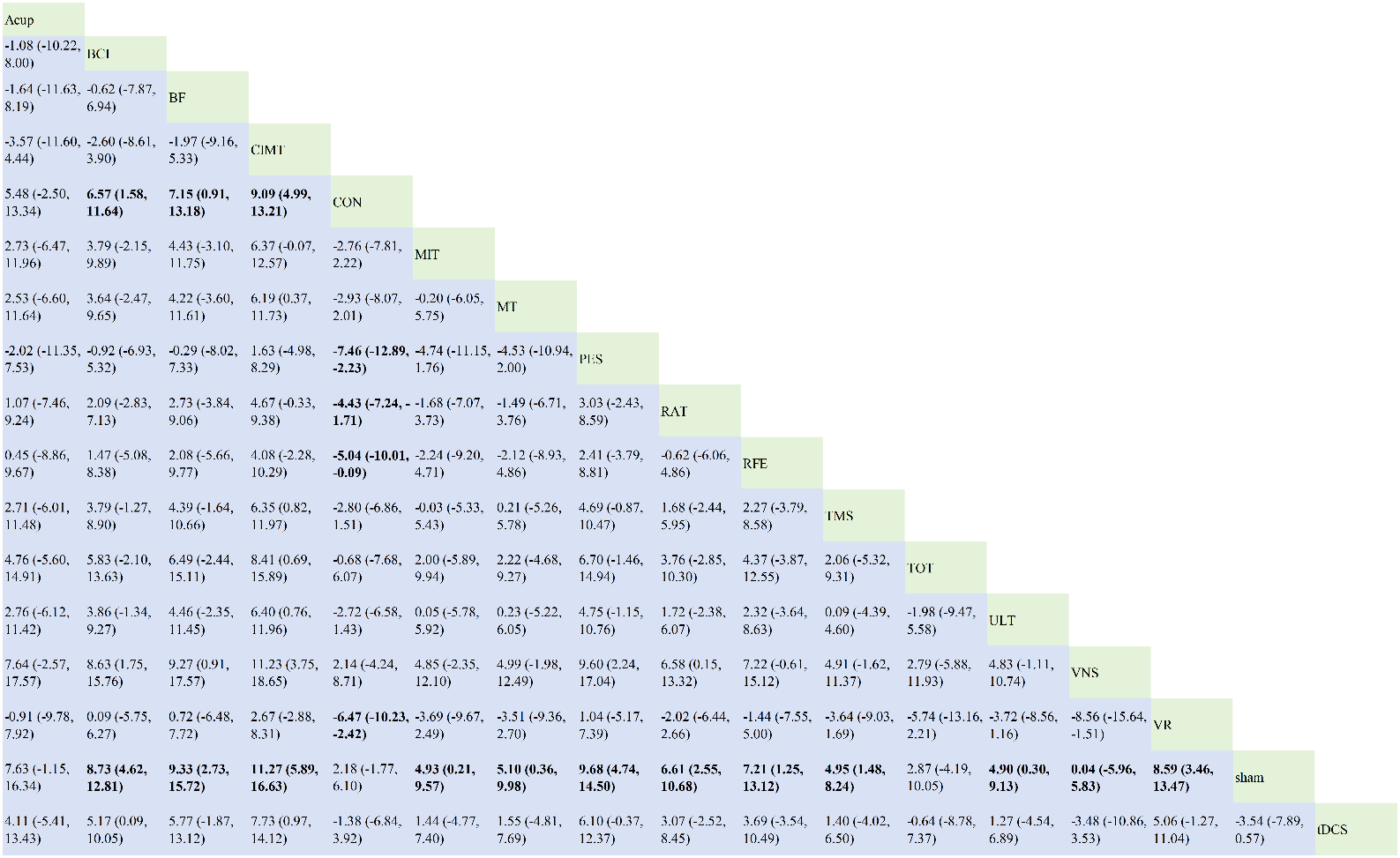
**

Note: The results are reported based on the lower triangular matrix of the ADDIS league table. Therefore, effect estimates are expressed as column-defining treatments versus row-defining treatments (column vs row). Bold values indicate statistically significant differences.

**Figure S13. Sensitivity Analysis Restricted to Add-On Effect Studies**

**(A)** **Ranking Probability Plot**

**
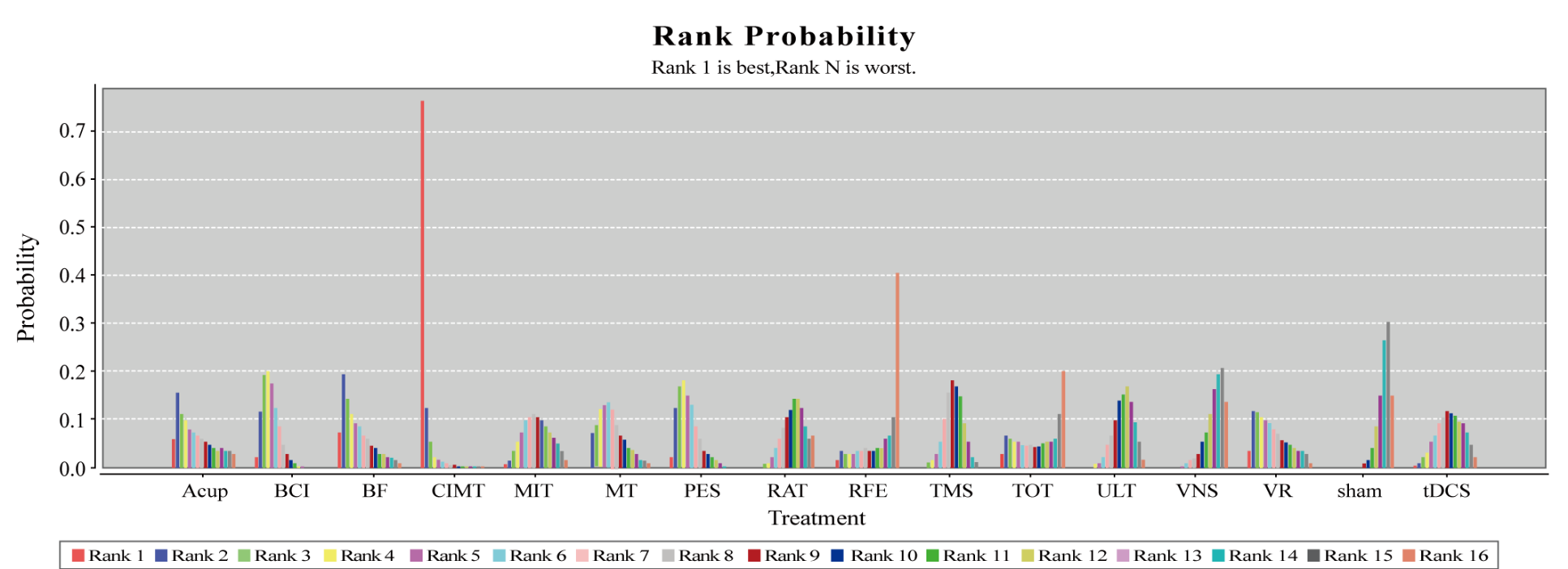
**

**(B) League Table**

**
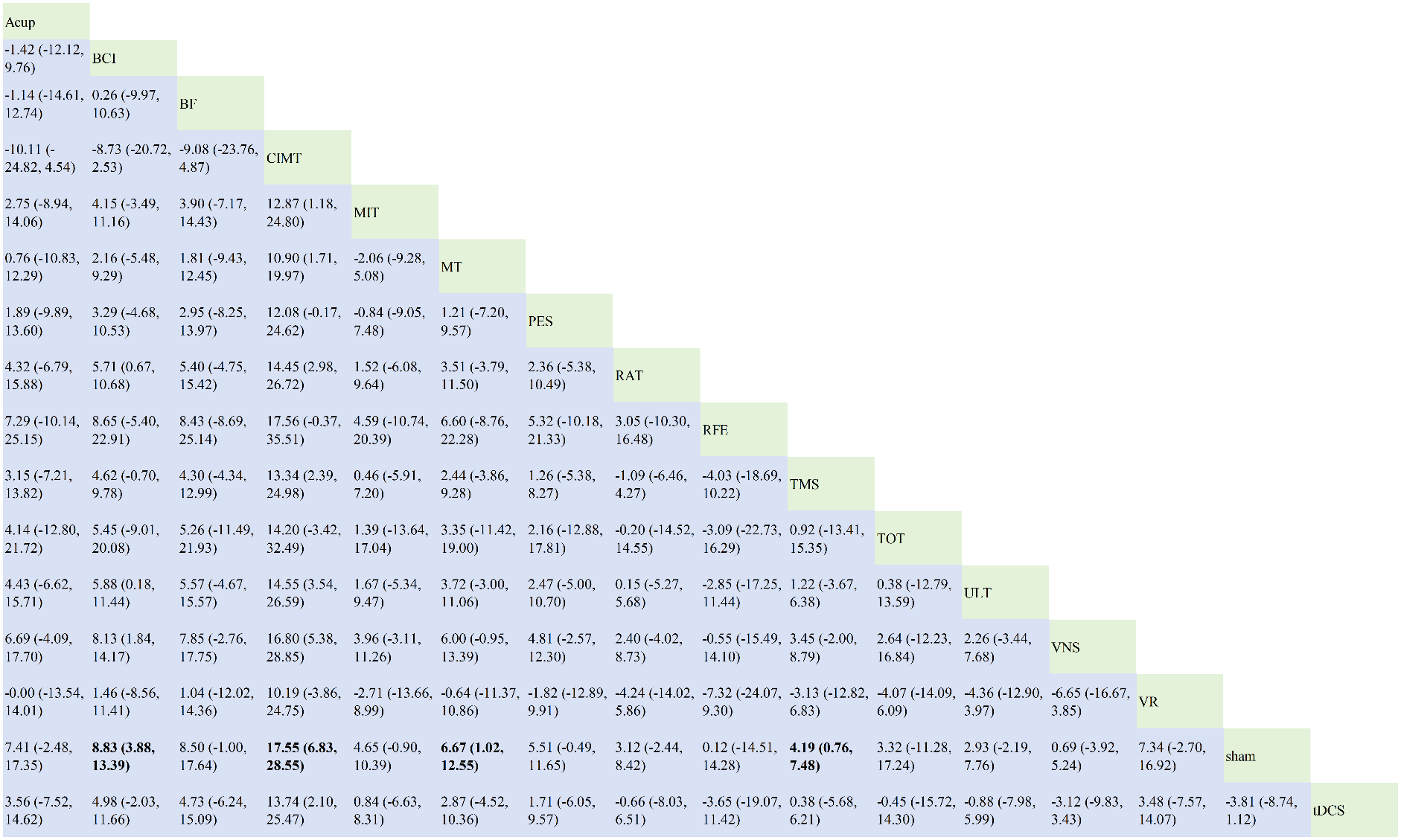
**

Note: The results are reported based on the lower triangular matrix of the ADDIS league table. Therefore, effect estimates are expressed as column-defining treatments versus row-defining treatments (column vs row). Bold values indicate statistically significant differences.

**Figure S14. Sensitivity Analysis Restricted to Monotherapy Studies**

**(A)** **Ranking Probability Plot**

**
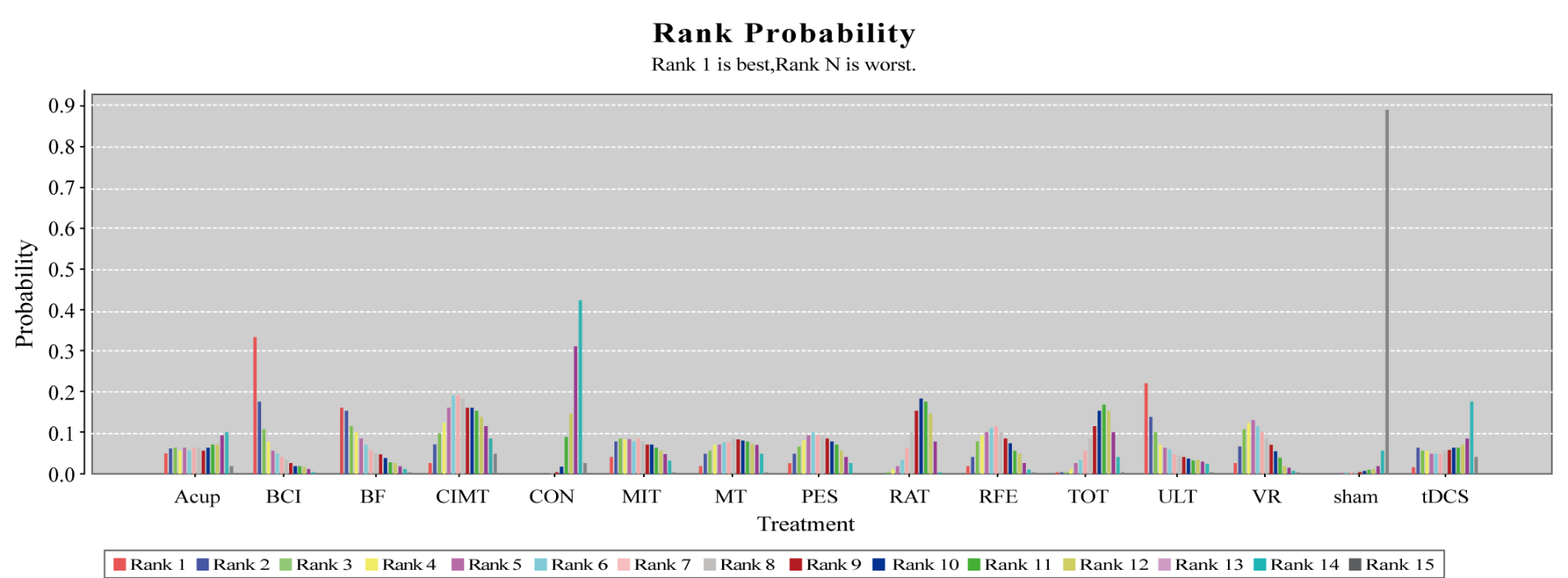
**

**(B) League Table**

**
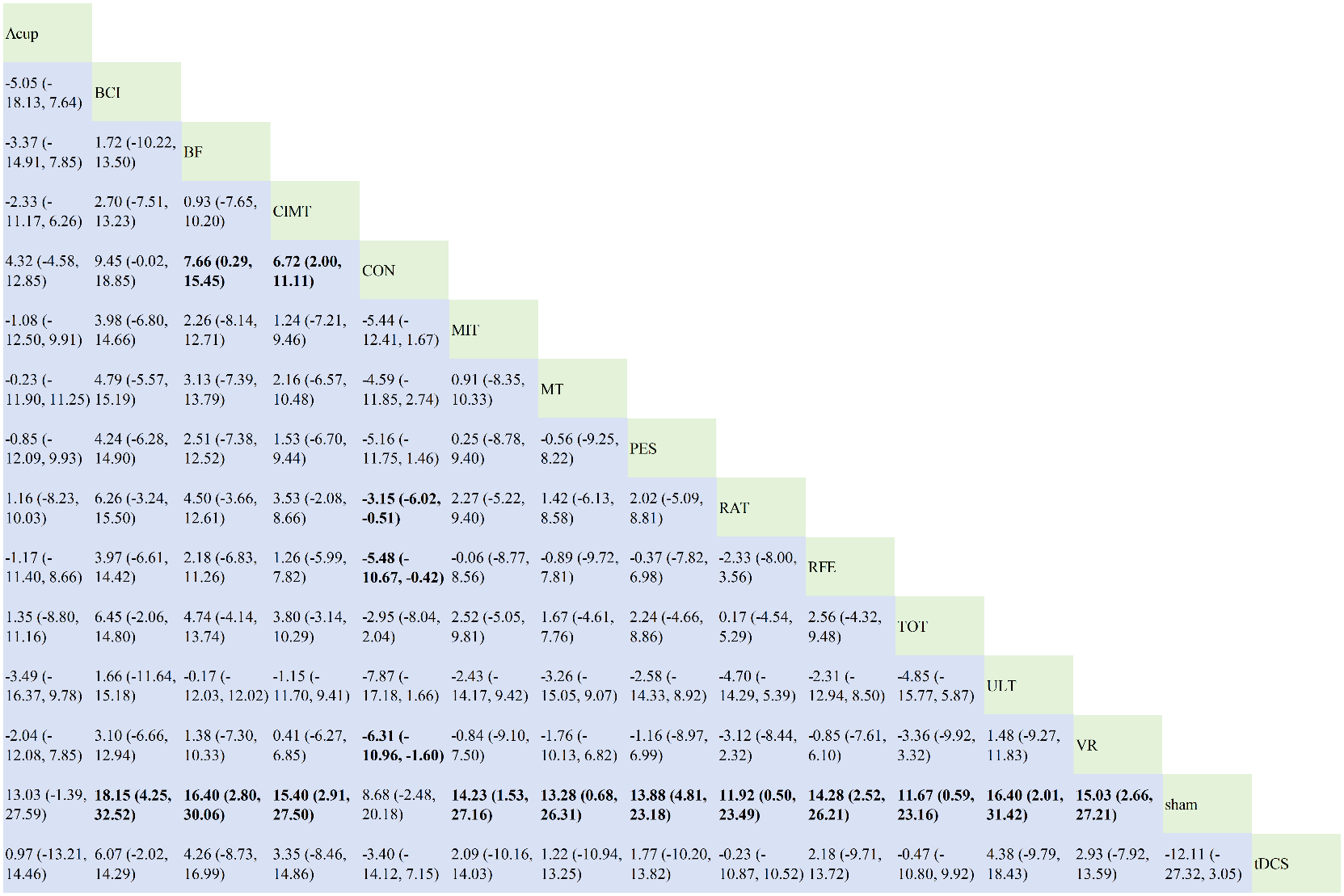
**

Note: The results are reported based on the lower triangular matrix of the ADDIS league table. Therefore, effect estimates are expressed as column-defining treatments versus row-defining treatments (column vs row). Bold values indicate statistically significant differences.

**Figure S15. Funnel plot of FMA-UE, ADL, ARAT, WMFT, BBT, GS and MAS.**

**(A)** **The control group was CON**


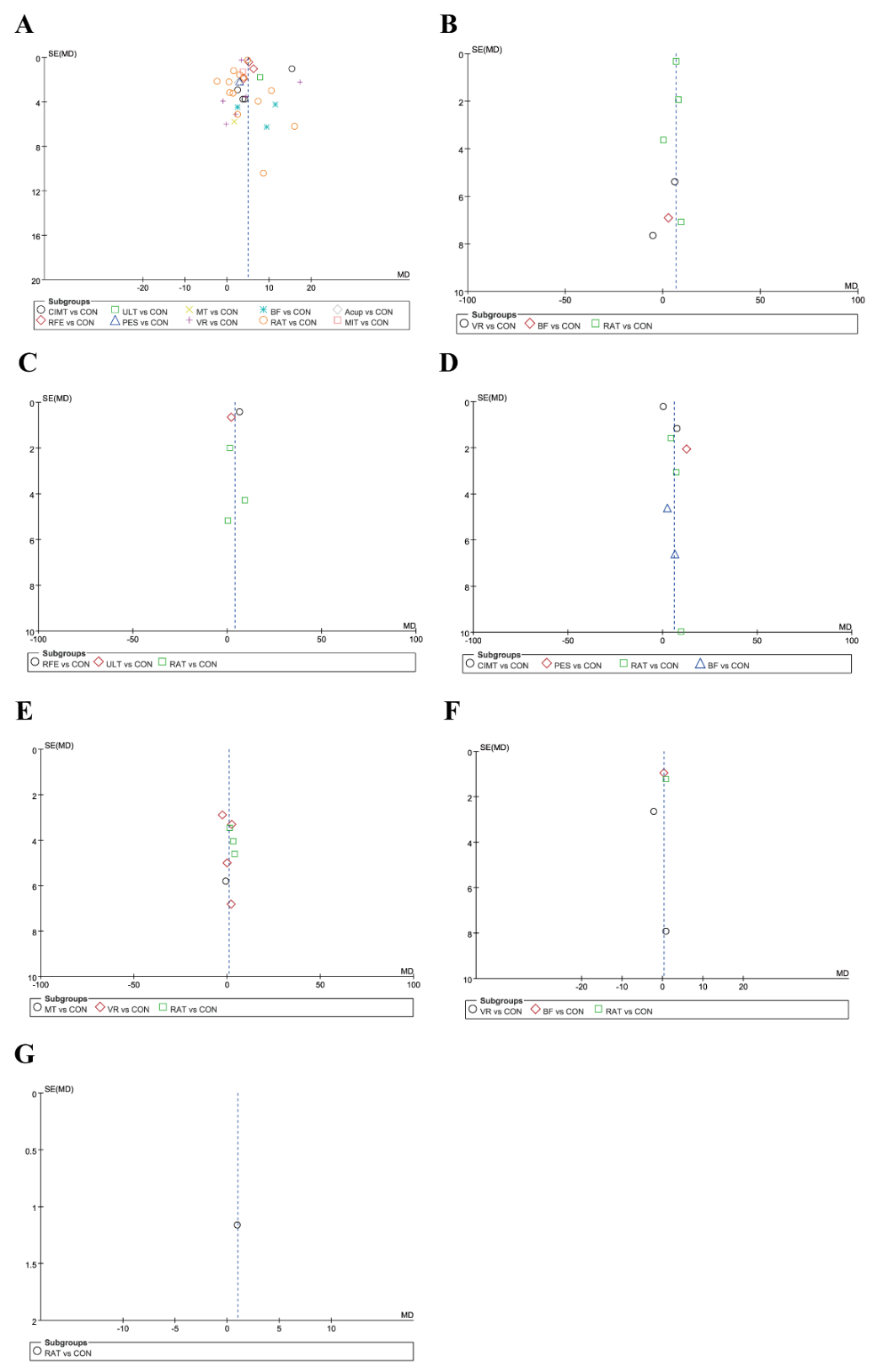


**(B) The control group was sham**


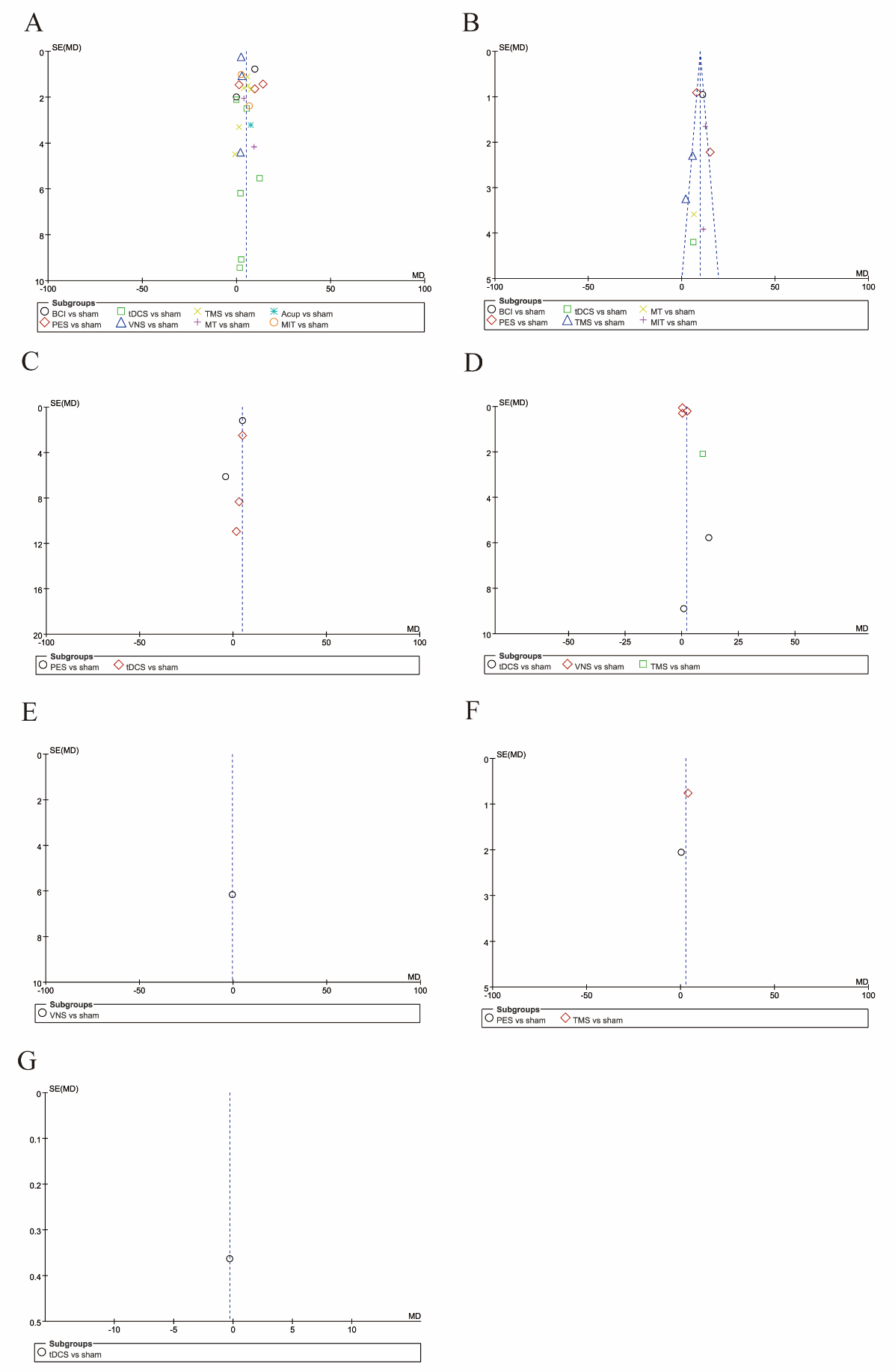


Notes: (A) Funnel plot of improvement of FMA-UE; (B) Funnel plot of improvement of ADL; (C) Funnel plot of improvement of ARAT; (D) Funnel plot of improvement of WMFT; (E) Funnel plot of improvement of BBT; (F) Funnel plot of improvement of GS; (G) Funnel plot of reduction of MAS.

**Table S1. PRISMA NMA Checklist of Items to Include When Reporting A Systematic Review Involving a Network Meta-analysis**

| **Section/Topic** | **Item #** | **Checklist Item** | **Reported on Page #** |
| --- | --- | --- | --- |
| **TITLE** |  |  |  |
| Title | 1 | Identify the report as a systematic review *incorporating a network meta-analysis (or related form of meta-analysis).* | 1 |
|  |  |  |  |
| **ABSTRACT** |  |  |  |
| Structured summary | 2 | Provide a structured summary including, as applicable:  **Background:** main objectives  **Methods:** data sources; study eligibility criteria, participants, and interventions; study appraisal; and *synthesis methods, such as network meta-analysis.*  **Results:** number of studies and participants identified; summary estimates with corresponding confidence/credible intervals; *treatment rankings may also be discussed. Authors may choose to summarize pairwise comparisons against a chosen treatment included in their analyses for brevity.*  **Discussion/Conclusions:** limitations; conclusions and implications of findings.  **Other:** primary source of funding; systematic review registration number with registry name. | 1-2 |
|  |  |  |  |
| **INTRODUCTION** |  |  |  |
| Rationale | 3 | Describe the rationale for the review in the context of what is already known*, including mention of why a network meta-analysis has been conducted.* | 2 |
| Objectives | 4 | Provide an explicit statement of questions being addressed, with reference to participants, interventions, comparisons, outcomes, and study design (PICOS). | 2-3 |
|  |  |  |  |
| **METHODS** |  |  |  |
| Protocol and registration | 5 | Indicate whether a review protocol exists and if and where it can be accessed (e.g., Web address); and, if available, provide registration information, including registration number. | 2 |
| Eligibility criteria | 6 | Specify study characteristics (e.g., PICOS, length of follow-up) and report characteristics (e.g., years considered, language, publication status) used as criteria for eligibility, giving rationale. *Clearly describe eligible treatments included in the treatment network, and note whether any have been clustered or merged into the same node (with justification).* | 2-3 |
| Information sources | 7 | Describe all information sources (e.g., databases with dates of coverage, contact with study authors to identify additional studies) in the search and date last searched. | 3 |
| Search | 8 | Present full electronic search strategy for at least one database, including any limits used, such that it could be repeated. | Appendix S1 |
| Study selection | 9 | State the process for selecting studies (i.e., screening, eligibility, included in systematic review, and, if applicable, included in the meta-analysis). | 3-4 |
| Data collection process | 10 | Describe method of data extraction from reports (e.g., piloted forms, independently, in duplicate) and any processes for obtaining and confirming data from investigators. | 3-4 |
| Data items | 11 | List and define all variables for which data were sought (e.g., PICOS, funding sources) and any assumptions and simplifications made. | 3-4 |
| **Geometry of the network** | **S1** | Describe methods used to explore the geometry of the treatment network under study and potential biases related to it. This should include how the evidence base has been graphically summarized for presentation, and what characteristics were compiled and used to describe the evidence base to readers. | 9 |
| Risk of bias within individual studies | 12 | Describe methods used for assessing risk of bias of individual studies (including specification of whether this was done at the study or outcome level), and how this information is to be used in any data synthesis. | 4 |
| Summary measures | 13 | State the principal summary measures (e.g., risk ratio, difference in means). *Also describe the use of additional summary measures assessed, such as treatment rankings and surface under the cumulative ranking curve (SUCRA) values, as well as modified approaches used to present summary findings from meta-analyses.* | 4-5 |
| Planned methods of analysis | 14 | Describe the methods of handling data and combining results of studies for each network meta-analysis. This should include, but not be limited to:   - *Handling of multi-arm trials;* - *Selection of variance structure;* - *Selection of prior distributions in Bayesian analyses; and* - *Assessment of model fit.* | 4-5 |
| **Assessment of Inconsistency** | **S2** | Describe the statistical methods used to evaluate the agreement of direct and indirect evidence in the treatment network(s) studied. Describe efforts taken to address its presence when found. | 4-5 |
| Risk of bias across studies | 15 | Specify any assessment of risk of bias that may affect the cumulative evidence (e.g., publication bias, selective reporting within studies). | 5 |
| Additional analyses | 16 | Describe methods of additional analyses if done, indicating which were pre-specified. This may include, but not be limited to, the following:   - Sensitivity or subgroup analyses; - Meta-regression analyses; - *Alternative formulations of the treatment network; and* - *Use of alternative prior distributions for Bayesian analyses (if applicable).* | 5 |
|  |  |  |  |
| **RESULTS†** |  |  |  |
| Study selection | 17 | Give numbers of studies screened, assessed for eligibility, and included in the review, with reasons for exclusions at each stage, ideally with a flow diagram. | 5, Figure 1 |
| **Presentation of network structure** | **S3** | Provide a network graph of the included studies to enable visualization of the geometry of the treatment network. | Figure 3 |
| **Summary of network geometry** | **S4** | Provide a brief overview of characteristics of the treatment network. This may include commentary on the abundance of trials and randomized patients for the different interventions and pairwise comparisons in the network, gaps of evidence in the treatment network, and potential biases reflected by the network structure. | 9-10 |
| Study characteristics | 18 | For each study, present characteristics for which data were extracted (e.g., study size, PICOS, follow-up period) and provide the citations. | Table S3 |
| Risk of bias within studies | 19 | Present data on risk of bias of each study and, if available, any outcome level assessment. | 6 |
| Results of individual studies | 20 | For all outcomes considered (benefits or harms), present, for each study: 1) simple summary data for each intervention group, and 2) effect estimates and confidence intervals. *Modified approaches may be needed to deal with information from larger networks.* | Table S4 |
| Synthesis of results | 21 | Present results of each meta-analysis done, including confidence/credible intervals. *In larger networks, authors may focus on comparisons versus a particular comparator (e.g. placebo or standard care), with full findings presented in an appendix. League tables and forest plots may be considered to summarize pairwise comparisons.* If additional summary measures were explored (such as treatment rankings), these should also be presented. | 9-10 |
| **Exploration for inconsistency** | **S5** | Describe results from investigations of inconsistency. This may include such information as measures of model fit to compare consistency and inconsistency models, *P* values from statistical tests, or summary of inconsistency estimates from different parts of the treatment network. | Table S6, 7, 8 |
| Risk of bias across studies | 22 | Present results of any assessment of risk of bias across studies for the evidence base being studied. | 13 |
| Results of additional analyses | 23 | Give results of additional analyses, if done (e.g., sensitivity or subgroup analyses, meta-regression analyses*, alternative network geometries studied, alternative choice of prior distributions for Bayesian analyses,* and so forth). | 10-13 |
|  |  |  |  |
| **DISCUSSION** |  |  |  |
| Summary of evidence | 24 | Summarize the main findings, including the strength of evidence for each main outcome; consider their relevance to key groups (e.g., healthcare providers, users, and policy-makers). | 13-14 |
| Limitations | 25 | Discuss limitations at study and outcome level (e.g., risk of bias), and at review level (e.g., incomplete retrieval of identified research, reporting bias). *Comment on the validity of the assumptions, such as transitivity and consistency. Comment on any concerns regarding network geometry (e.g., avoidance of certain comparisons).* | 15-16 |
| Conclusions | 26 | Provide a general interpretation of the results in the context of other evidence, and implications for future research. | 14-15 |
|  |  |  |  |
| **FUNDING** |  |  |  |
| Funding | 27 | Describe sources of funding for the systematic review and other support (e.g., supply of data); role of funders for the systematic review. This should also include information regarding whether funding has been received from manufacturers of treatments in the network and/or whether some of the authors are content experts with professional conflicts of interest that could affect use of treatments in the network. | 16 |

PICOS = population, intervention, comparators, outcomes, study design.

* Text in italics indicateS wording specific to reporting of network meta-analyses that has been added to guidance from the PRISMA statement.

† Authors may wish to plan for use of appendices to present all relevant information in full detail for items in this section.

**Table S2. Information Related to 15 Interventions.**

| Intervention | No. of trials | No. of participants | Detailed description |
| --- | --- | --- | --- |
| CIMT | 8 | 193 | The signature protocol for CIMT contains three components : (1) intensive graded practice of the paretic upper limb aimed at enhancing task-specific use of the affected limb ; (2) constraining or FU of the non-paretic upper limb with a mitt to promote the use of the more impaired limb during 90% of the waking hours; and (3) adherence-enhancing behavioral methods designed to transfer the gains obtained in the clinical setting or laboratory to the patients' real-world environment |
| RFE | 3 | 74 | RFE is a neurofacilitation-based rehabilitation approach that combines voluntary movement attempts with peripheral facilitation techniques and intensive repetition to promote motor recovery after stroke. |
| TOT | 5 | 114 | Task-oriented training includes a wide range of interventions, such as treadmill training, overground walking training, cycling programs, endurance and circuit training, sit-to-stand exercises, and reaching tasks aimed at improving balance. In addition, functional task-based arm training is commonly incorporated. |
| ULT | 11 | 430 | ULT promotes functional recovery by gradually increasing the complexity of activities through coordinated joint movements and bilateral upper limb exercises. |
| BCI | 7 | 149 | BCI-based training in patients after stroke typically involves asking the patient to mentally perform movements of the paretic limb, while non-invasive sensors record brain activity associated with motor intention. These signals are then decoded in real time and converted into control commands for external devices, which provide feedback to the patient regarding task performance. |
| PES | 6 | 237 | PES delivers electrical stimuli to nerves and muscles via surface electrodes to enhance muscle contraction or improve voluntary motor control, including neuromuscular electrical stimulation, transcutaneous acupoint electrical stimulation, and functional electrical stimulation. |
| tDCS | 8 | 183 | A technique of brain electric stimulation therapy which uses constant, low current delivered via electrodes placed on various locations on the scalp, which enhances neuronal excitability and induces neuroplasticity. |
| VNS | 4 | 117 | VNS delivers electrical impulses to the brain via the vagus nerve, thereby modulating cortical excitability and promoting neuroplasticity. |
| TMS | 9 | 251 | TMS is a therapeutic technique that generates transient magnetic fields by applying an electromagnetic coil over the scalp. These magnetic fields penetrate the cerebral cortex to stimulate the central nervous system, thereby modulating cortical excitability. |
| MT | 10 | 219 | MT involves placing a mirror between the limbs, such that movements of the non-affected limb create a visual illusion of normal movement in the affected limb. This visual feedback has been shown to activate multiple brain regions associated with motor execution, somatosensory processing, and pain modulation. |
| VR | 12 | 396 | VR involves the use of computer hardware and software to create interactive simulations that provide a virtual training environment on movement execution or task achievement. |
| BF | 4 | 91 | BF is a technique that uses instruments to provide patients with real-time feedback on physiological activity, thereby enhancing their awareness of their own movements or functions and strengthening their capacity for voluntary control. |
| RAT | 22 | 674 | RAT is a device-based intervention that enables passive limb movement while providing assistance for single-joint motions and coordinated motor control. |
| Acup | 2 | 51 | The occupational discipline of the traditional Chinese methods of acupuncture therapy for treating disease by inserting needles along specific pathways or meridians. |
| MIT | 5 | 139 | MIT involves the internal simulation and mental rehearsal of motor actions without overt physical movement. |

Table S3. Summary table of stimulation parameters

| study | Intervention | Stimulation parameters |
| --- | --- | --- |
| Ohnishi et al. 2022 | NMES | 50 Hz; 50 μs pulse width; maximum tolerated intensity; 5 s on/5 s off (1:1 duty cycle) |
| Wang et al. 2023a | TEAS | TEAS at LI10 and TE5; 2 Hz; 300 μs pulse width; maximum tolerable intensity |
| Peng et al. 2015 | TEAS | LI15, LI11, TE5, and LI4; 100 Hz; biphasic symmetrical square wave; 250 μs pulse width; 0–100 mA (maximum tolerated intensity) |
| Xia et al. 2021 | TEAS | LI15, LI11, LI10, and TE5 (bilateral); 100 Hz; 200 μs pulse width; maximum tolerated intensity (up to 100 mA) |
| Au-Yeung et al. 2014 | ES | 20 Hz; 200 μs; 2.5–3 × sensory threshold (maximum tolerated intensity); circular surface electrodes (2.2 cm diameter) |
| Knutson et al. 2026 | FES | / |
| Sharma et al. 2020 | TMS | Fc3/Fc4; 1 Hz; 750 pulses (75 trains); inter-train interval 45 s; 110% RMT |
| Wang et al. 2023b | TMS | 1 Hz; 90% RMT; 10 s trains with 2 s inter-train interval; 20 min/session |
| Long et al. 2018 | TMS | non-dominant M1; 1 Hz; 90% RMT; 1000 pulses |
| Dou et al. 2019 | TMS | contralesional M1; 1 Hz; 80% MT; 1200 pulses (2 × 600), inter-train interval 20 s |
| Sun et al. 2024 | TMS | 1 Hz; 90% RMT; 1000 pulses (100 trains), 10 s on / 2 s off; 20 min |
| Sun et al. 2022 | TMS | 1 Hz; 90% RMT; 656 pulses (82 trains); 8 s trains with 3 s inter-train interval; 15 min |
| Yang et al. 2020 | TMS | 1 Hz; 80% MT; 1000 pulses (100 trains); 10 s on / 2 s off; 20 min |
| Zhao et al. 2015 | TMS | ipsilesional M1; 1 Hz; 100% RMT; 1200 pulses; 20 min |
| Seniów et al. 2012 | TMS | 1 Hz; 90% rMT; 1800 pulses; 20 min |
| Allman et al. 2016 | tDCS | anodal tDCS; 1 mA; 20 min; 10 s ramp-up / 10 s ramp-down |
| Gerloff et al. 2024 | tDCS | anodal tDCS; 1 mA; 20 min; 35 cm² electrodes (5 × 7 cm); current density 0.03 mA/cm² |
| Vimolratana et al. 2024 | tDCS | anodal tDCS; 1.5 mA; 20 min; 30 s ramp-up / ramp-down; MINDD STIM (Ybrain, Korea) |
| Wei et al. 2022 | tDCS | 2 mA; 30 s ramp-up / 30 s ramp-down |
| Yin et al. 2015 | tDCS | anodal tDCS; 1.0 mA; 20 min |
| Ming et al. 2025 | tDCS | anodal tDCS; ipsilesional M1; contralateral supraorbital area; 2 mA; 25 cm² electrodes |
| Lee et al. 2014 | tDCS | tDCS; 2 mA; 20 min; stimulation site determined by lowest excitability threshold (motor hotspot) |
| Hou et al. 2026 | tDCS | 2 mA; 20 min/session; 1 session/day, 6 sessions/week for 3 weeks (18 sessions total) |
| Dawson et al. 2021 | VNS | 30 Hz; 100 μs pulse width; 0.5 s trains; 0.6–0.8 mA |
| Kimberley et al. 2018 | VNS | active VNS; 30 Hz; 100 μs pulse width; 0.8 mA; 0.5 s bursts; task-triggered + home stimulation (30 min, every 10 s) |
| Zhang et al. 2020 | VNS | 20 Hz; 0.5 mA; 30 s stimulation; every 2 min; 30 min/session |
| Zhang et al. 2023 | taVNS | 20 Hz; 600 μs pulse width; 0.5 mA; 30 s on / 2 min off |

Note: CIMT: constraint-induced movement therapy; RFE: repetitive facilitative exercise; TOT: task-oriented training; ULT: upper limb training; BCI: brain computer interface; PES: peripheral electrical stimulation; TMS: transcranial magnetic stimulation; RAT: robot-assisted training; BF: biofeedback; tDCS: transcranial direct current stimulation; MT: mirror therapy; VNS: Vagus nerve stimulation; MIT: motor imagery training; VR: virtual reality; Acup: acupuncture.

**Table S4. Main characteristics of included RCTs.**

| Study | Country | Group | Sample size (M/F) | Age, y(mean±SD) | Intervention category | Intervention frequency and duration | Outcome measures | Time since stroke | Phase | Levels of severity | First-ever | Stroke type(ischemic/hemorrhagic) | Hemiplegic side(L/R) | Handness (L/R/A) | Adverse events |
| --- | --- | --- | --- | --- | --- | --- | --- | --- | --- | --- | --- | --- | --- | --- | --- |
| Ohnishi et al. 2022 | Japan | RFE | 22(15/7) | 60.4±11.5 | RFE | 7d/wk, 4wk | FMA-UE | 31.3±13.6d | subacute | severe | Yes | 10/12 | 10/12 | / | / |
|  |  | PES | 25(16/9) | 57.3±14.0 | NMES |  |  | 29.6±12.1d |  |  |  | 13/12 | 14/11 |  |  |
|  |  | CON | 26(17/9) | 63.2±13.0 | CON |  |  | 29.4±9.3d |  |  |  | 13/13 | 14/12 |  |  |
| Wang et al. 2023a | China | PES | 102(77/25) | 60.6±12.5 | CON+TEAS | 5d/wk, 6wk | FMA-UE | 95.6±97.6d | / | / | Yes | 68/34 | 42/60 | / | No |
|  |  | sham | 102(72/30) | 61.9±10.5 | CON+Sham TEAS |  |  | 95.7 ± 90.8d |  |  |  | 71/31 | 46/56 |  |  |
| Peng et al. 2015 | China | PES | 21(9/12) | 66.4±10.8 | TEAS | 5d/wk, 3wk | FMA-UE, MBI | 2.25±0.87M | subacute | Sever | Yes | 14/7 | / | / | / |
|  |  | sham | 20(10/10) | 65.5±11.2 | Sham TEAS |  |  | 2.02±1.05M |  |  |  | 13/7 | / |  |  |
| Xia et al. 2021 | China | PES | 24(18/6) | 56.20±9.91 | CON+TEAS | Once/d, 6d/wk, 4wk | FMA-UE, ARAT, WMFT, MBI | 5.00±2.58W | subacute | Severe | Yes | 15/9 | / | / | / |
|  |  | sham | 24(17/7) | 56.50±7.90 | CON+Sham TEAS |  |  | 5.20±2.57W |  |  |  | 12/12 | / |  |  |
| Sharma et al. 2020 | India | TMS | 47(33/14) | 54.85±13.39 | CON+LF-rTMS | 5d/wk, 2wk | MBI, FMA-UE | 4.96±1.56d | subacute | Moderate | Yes | 47/0 | / | / | Yes |
|  |  | sham | 49(34/15) | 52.89±14.95 | CON+Sham LF-rTMS |  |  | 4.68±1.26d |  |  |  | 49/0 | / |  |  |
| Wang et al. 2023b | China | TMS | 23(17/6) | 61.78±12.63 | CON+LF-rTMS | Once/d, 14d | FMA-UE, MBI | 90.52±17.47d | subacute | Severe | / | 11/12 | 14/9 | / | / |
|  |  | RAT | 23(12/11) | 61.78±11.37 | CON+RAT |  |  | 92.30±16.77d |  |  |  | 9/14 | 11/12 |  |  |
| Long et al. 2018 | China | TMS | 21(16/5) | 57±11.78 | CON+LF-rTMS | Once/d, 15d | FMA-UE | 19.57±2.34d | subacute | Moderate | Yes | 11/10 | 10/11 | / | No |
|  |  | sham | 20(15/5) | 56.85±5.48 | CON+Sham LF-rTMS |  |  | 19.05±2.74d |  |  |  | 10/10 | 9/11 |  |  |
| Dou et al. 2019 | China | TMS | 15(10/5) | 62.20±10.59 | CON+LF-rTMS | 5d/wk, 3wk | FMA-UE | 23.20±17.13d | subacute | Severe | Yes | 10/5 | 6/9 | / | / |
|  |  | sham | 15(9/6) | 56.93±7.34 | CON+Sham LF-rTMS |  |  | 22.33±15.93d |  |  |  | 8/7 | 9/6 |  |  |
| Sun et al. 2024 | China | TMS | 40(24/16) | 59.90±8.79 | CON+LF-rTMS | Once/d, 5d/wk, 4wk | FMA-UE, WMFT, MBI | 2.20±1.04d | acute | / | Yes | / | 19/21 | / | Yes |
|  |  | sham | 40(23/17) | 60.73±8.71 | CON+Sham LF-rTMS |  |  | 2.40±1.03d |  |  |  | / | 21/19 |  |  |
| Sun et al. 2022 | China | TMS | 20(10/10) | 60.55±7.45 | CON+LF-rTMS | Twice/d,6d/wk, 4wk | FMA-UE, MBI | 61.15±17.56d | subacute | Severe | Yes | 20/0 | 15/5 | / | / |
|  |  | ULT | 20(11/9) | 59.35±6.82 | CON+ULT |  |  | 56.05±15.69d |  |  |  | 20/0 | 13/7 |  |  |
| Yang et al. 2020 | China | TMS | 45(24/21) | 53.2±10.0 | CON+LF-rTMS | Once/d, 5d/wk, 4wk | FMA-UE | 52.4±33.1d | subacute | Severe | Yes | 26/19 | 29/16 | / | / |
|  |  | BF | 45(26/19) | 54.5±9.8 | CON+BF |  |  | 53.0±31.6d |  |  |  | 25/23 | 25/20 |  |  |
| Zhao et al. 2015 | China | TMS | 20(15/5) | 57.7±9.5 | CON+LF-rTMS | Once/d, 10d | FMA-UE, GS | 54.1±14.3d | subacute | Moderate | / | 20/0 | 7/13 | / | / |
|  |  | sham | 20(14/6) | 55.5±13.2 | CON+Sham LF-rTMS |  |  | 52.1±13.8 |  |  |  | 20/0 | 7/13 |  |  |
| Allman et al. 2016 | United Kingdom | tDCS | 13(4/9) | 59.5±12.1 | CON+tDCS | Once/d, 9d | FMA-UE, ARAT, WMFT | 51.2±33.4M | chronic | Moderate | Yes | / | 9/4 | / | / |
|  |  | sham | 11(3/8) | 66.8±10.4 | CON+Sham tDCS |  |  | 56.6±39.8M |  |  |  | / | 8/3 |  |  |
| Gerloff et al. 2024 | Germany | tDCS | 58(38/20) | 67(58–74) | CON+tDCS | Once/d, 5d/wk, 2wk | FMA-UE | 21(10–30)d | subacute | Moderate | Yes | / | 31/27 | / | Yes |
|  |  | sham | 61(37/24) | 68(59–75) | CON+Sham tDCS |  |  | 19(9–27)d |  |  |  | / | 24/37 |  |  |
| Vimolratana et al. 2024 | Thailand | tDCS | 15(6/9) | 52.53±15.05 | CON+tDCS | Once/d, 5d | FMA-UE | 4.00(3.00;6.00)d | acute | Moderate | Yes | / | / | 2/13/0 | / |
|  |  | sham | 15(8/7) | 62.27±9.68 | CON+Sham tDCS |  |  | 4.00(4.00;9.00)d |  |  |  | / | / | 4/11/0 |  |
| Wei et al. 2022 | China | tDCS | 9(7/2) | 54.4±16.6 | CON+tDCS | 5x/wk, 3wk | FMA-UE, ARAT, MAS | 25.9±32.4M | chronic | Moderate | Yes | / | 5/4 | / | / |
|  |  | sham | 8(6/2) | 68.4±7.5 | CON+Sham tDCS |  |  | 17.8±20.2M |  |  |  | / | 3/5 |  |  |
| Yin et al. 2015 | China | tDCS | 40(27/13) | 55.70±12.32 | CON+tDCS | Once/d, 5d/wk, 4wk | FMA-UE, ARAT, MBI | 31.55±20.13d | subacute | Severe | Yes | 21/19 | / | / | / |
|  |  | sham | 40(30/10) | 57.68±13.54 | CON+Sham tDCS |  |  | 35.90±19.60d |  |  |  | 23/17 | / |  |  |
| Gurbuz et al. 2016 | Turkey | MT | 16(10/6) | 60.9±10.9 | CON+MT | 5x/wk, 4wk | FMA-UE | 46.1±43.3d | subacute | Severe | Yes | 15/1 | 8/8 | 1/15/0 | / |
|  |  | sham | 15(7/8) | 60.8±20.0 | CON+Sham MT |  |  | 42.4±37.8d |  |  |  | 10/5 | 6/9 | 0/15/0 |  |
| Hsu et al. 2022 | China | MT | 17(7/10) | 56.7±11.5 | MT | 2x/wk, 9wk | FMA-UE, BBT | 39.8±28.8M | chronic | Moderate | / | / | 9/8 | / | No |
|  |  | CON | 17(5/12) | 56.9±13.0 | CON |  |  | 38.1±26.6M |  |  |  | / | 8/9 |  |  |
| Kaviraja et al. 2021 | India | MT | 15 | / | CON+MT | 5d/wk, 4wk | FMA-UE | 2-12M | subacute | Moderate | Yes | / | / | / | / |
|  |  | CIMT | 15 | / | CON+CIMT |  |  | 2-12M |  |  |  | / | / |  |  |
| Kim et al. 2016 | Korea | MT | 12(8/4) | 45.2±4.7 | MT | 5x/wk, 4wk | ARAT, BBT, FMA-UE | >6M | chronic | Moderate | / | 4/8 | 8/4 | / | / |
|  |  | CON | 13(8/5) | 52.6±3.0 | CON |  |  | >6M |  |  |  | 4/9 | 8/5 |  |  |
| Lim et al. 2016 | Korea | MT | 30(11/19) | 65.3 | CON+MT | 5d/wk, 4wk | FMA-UE, MBI | 49.4d | subacute | Moderate | / | 8/22 | 15/15 | / | / |
|  |  | sham | 30(10/20) | 64.5 | CON+Sham MT |  |  | 53.7d |  |  |  | 11/19 | 16/14 |  |  |
| Zhuang et al. 2021 | China | MT | 18(12/6) | 54.0±24.00 | CON+MT | 5d/wk, 4wk | FMA-UE, BBT | 4.0±5.25M | subacute | Moderate | Yes | 5/13 | 9/9 | / | / |
|  |  | sham | 18(12/6) | 58.0±22.75 | CON+Sham MT |  |  | 5.0±7.25M |  |  |  | 7/11 | 10/8 |  |  |
| Du et al. 2016 | China | MT | 35(21/14) | 55.0±7.7 | MT | Twice/d,5d/wk, 12wk | ARAT | <6M | subacute | Severe | Yes | 30/5 | 9/26 | / | / |
|  |  | CIMT | 34(20/14) | 56.3±6.7 | CIMT |  |  | <6M |  |  |  | 27/7 | 7/27 |  |  |
| de Sire et al. 2025 | Italy | MT | 40(19/21) | 71.13 | CON+MT | 5d/wk, 8wk | GS | / | / | / |  | / | 15/25 | / | / |
|  |  | CIMT | 40(21/19) | 73.05 | CON+CIMT |  |  | / |  |  | / | / | 15/25 |  |  |
| Dawson et al. 2021 | United Kingdom | VNS | 53(34/19) | 59.1±10.2 | CON+VNS | 3x/wk, 6wk | FMA-UE, WMFT | 3.1±2.3Y | chronic | Moderate | No | 53/0 | 28/25 | 4/48/1 | Yes |
|  |  | sham | 55(36/19) | 61.1±9.2 | CON+Sham VNS |  |  | 3.3±2.6Y |  |  |  | 55/0 | 29/26 | 5/50/0 |  |
| Kimberley et al. 2018 | United States | VNS | 8(4/4) | 59.5±7.4 | CON+VNS | 3x/wk, 6wk | FMA-UE, WMFT, BBT | 18(11-43)M | chronic | Moderate | No | / | 7/1 | 0/8/0 | Yes |
|  |  | sham | 9(5/4) | 60.0±13.5 | CON+Sham VNS |  |  | 18(6.3-53)M |  |  |  | / | 4/5 | 1/7/1 |  |
| Zhang et al. 2020 | China | VNS | 21(10/11) | 66.10±1.491 | CON+VNS | Once/d, 5d/wk, 3wk | FMA-UE, WMFT | 38.00±1.459d | subacute | Severe | Yes | 21/0 | 8/13 | / | No |
|  |  | sham | 29(21/8) | 64.19±1.027 | CON+Sham VNS |  |  | 36.86±1.959d |  |  |  | 21/0 | 14/7 |  |  |
| Zhang et al. 2023 | China | VNS | 35(15/20) | 58.94±12.12 | CON+taVNS | Once/d, 5d/wk, 8wk | BBT, FMA-UE | 39.74±17.76d | subacute | Moderate | Yes | / | 17/18 | / | / |
|  |  | ULT | 35(18/17) | 56.60±13.23 | CON+BAT |  |  | 38.69±15.01d |  |  |  | / | 15/20 |  |  |
| Meng et al. 2018 | China | ULT | 64(34/30) | 55.38 ± 6.97 | HABIT | Twice/d,5d/wk, 2wk | FMA-UE, ARAT | 8.87 ± 2.69h | acute | Moderate | Yes | 50/14 | 35/29 | / | No |
|  |  | CON | 64(31/33) | 55.19 ± 7.82 | CON |  |  | 9.08 ± 2.35h |  |  |  | 45/19 | 33/31 |  |  |
| Thrane et al. 2015 | Norway | CIMT | 24(19/5) | 65.3±8.0 | CIMT | Once/d, 10d | WMFT, FMA-UE | 16.6±7.2d | subacute | Moderate | / | 23/1 | 14/10 | 8/16/0 | Yes |
|  |  | CON | 23(17/6) | 61.0±14.8 | CON |  |  | 18.0±6.5d |  |  |  | 20/3 | 11/12 | 13/10/0 |  |
| Zhang et al. 2025 | China | RFE | 26(16/10) | 46.8±11.7 | RFE | 5x/wk, 4wk | FMA-UE | 254.7±46.0d | chronic | Moderate | / | / | 12/14 | / | / |
|  |  | CON | 25(16/9) | 50.3±14.0 | CON |  |  | 270.1±55.8d |  |  |  | / | 14/11 |  |  |
| Zhang et al. 2024 | China | ULT | 39(23/16) | 62.8±10.1 | CON+BAT | 5d/wk, 4wk | FMA-UE, WMFT, BBT, MBI | 94.3±51.2d | subacute | Moderate | Yes | 25/14 | 17/22 | / | / |
|  |  | RAT | 38(22/16) | 64.3±10.7 | CON+RAT |  |  | 86.0±48.1d |  |  |  | 25/14 | 18/20 |  |  |
| Park 2022 | Korea | MIT | 17(8/9) | 61.42±8.12 | TOMP | 5x/wk,3wk | FMA-UE | 69.45±21.51M | chronic | Moderate | / | 12/5 | 2/15 | / | / |
|  |  | TOT | 17(8/9) | 62.12±10.20 | TOT |  |  | 72.22±20.37M |  |  |  | 11/6 | 1/16 |  |  |
| Jiang et al. 2022 | China | MIT | 50(29/21) | 57.18±7.03 | MIT | 5d/wk, 24wk | FMA-UE | 3.76±0.80M | subacute | Moderate | Yes | 50/0 | / | / | / |
|  |  | CON | 50(31/19) | 57.40±6.50 | CON |  |  | 3.82±0.94M |  |  |  | 50/0 | / |  |  |
| Wang et al. 2020 | China | MIT | 20(15/5) | 65.10±7.85 | CON+MIT | Once/d, 5d/wk, 4wk | FMA-UE, MBI | 13.70±2.52d | subacute | Severe | Yes | 17/3 | 16/4 | / | / |
|  |  | sham | 20(14/6) | 64.90±7.77 | CON+Sham MIT |  |  | 13.20±2.33d |  |  |  | 16/4 | 18/2 |  |  |
| Zhang et al. 2021 | China | MIT | 20(9/11) | 57.62±7.34 | CON+MIT | Once/d, 5d/wk, 4wk | FMA-UE, ARAT, MBI | 58.27±15.34d | subacute | Severe | Yes | 15/5 | 8/12 | / | / |
|  |  | MT | 20(11/9) | 56.08±5.18 | CON+MT |  |  | 58.27±16.63d |  |  |  | 14/6 | 7/13 |  |  |
| Adie et al. 2017 | United Kingdom | VR | 117(66/51) | 66.8±14.6 | CON+VR | Once/d, 6wk | ARAT | 57.3±48.3d | subacute | / | / | 104/13 | / | 15/102/0 | Yes |
|  |  | ULT | 118(65/53) | 68.0±11.9 | CON+ULT |  |  | 56.3±50.1d |  |  |  | 105/13 | / | 14/104/0 |  |
| Ali et al. 2024 | India | VR | 64(50/14) | 54.4±11.7 | CON+VR | Once/d, 6d/wk, 2wk | FMA-UE, ARAT | 30.0±54d | subacute | Moderate | / | 53/11 | 26/38 | / | / |
|  |  | ULT | 56(41/15) | 57.7±10.9 | CON+ULT |  |  | 22.5±45d |  |  |  | 47/9 | 26/30 |  |  |
| Anwar et al. 2022 | Pakistan | VR | 34 | 51.56±7.199 | VR | 3x/wk, 6wk | FMA-UE | / | chronic | Severe | Yes | / | / | / | / |
|  |  | CON | 34 | 51.35±5.783 | CON |  |  | / |  |  |  | / | / |  |  |
| Kuo et al. 2023 | China | VR | 19(13/6) | 57.47±6.99 | VR | 2x/wk, 9wk | BBT, GS | >6M | chronic | / | Yes | 10/9 | 7/12 | / | / |
|  |  | CON | 18(15/3) | 59.50±10.65 | CON |  |  | >6M |  |  |  | 4/14 | 9/9 |  |  |
| Oh et al. 2019 | Korea | VR | 17(12/5) | 57.4±12.2 | VR | 3x/wk, 6wk | FMA-UE, BBT, GS | <6M | subacute | Moderate | Yes | / | / | / | / |
|  |  | CON | 14(9/5) | 52.6±10.7 | CON |  |  | <6M |  |  |  | / | / |  |  |
| Schuster-Amft et al. 2018 | Switzerland | VR | 22(16/6) | 61.3±13.4 | VR | 4x/wk, 4wk | BBT | 2.4±2.4Y | chronic | / | Yes | 18 / 4 | 8/14 | / | No |
|  |  | CON | 32(23/9) | 61.2±11.2 | CON |  |  | 3.6±3.7Y |  |  |  | 25 / 7 | 16/16 |  |  |
| Shin et al. 2016 | Korea | VR | 24(19/5) | 57.2 ± 10.3 | VR | Once/d, 5x/wk, 4wk | FMA-UE | 13.6±13.4M | chronic | / | Yes | 15/9 | 15/9 | 1/23/0 | No |
|  |  | CON | 22(17/5) | 59.8 ± 13.0 | CON |  |  | 15.0±14.6M |  |  |  | 14/8 | 11/11 | 0/22/0 |  |
| Han et al. 2016 | China | VR | 15(10/5) | 61.4±8.1 | VR | 5d/wk, 2wk | FMA-UE | 25.1±13.7d | subacute | Moderate | Yes | 13/2 | 5/10 | / | Yes |
|  |  | CON | 15(9/6) | 58.8±9.5 | CON |  |  | 29.1±13.6d |  |  |  | 13/2 | 3/12 |  |  |
| Xiao et al. 2019 | China | VR | 16(10/6) | 56.12±9.01 | VR | 5d/wk, 4wk | FMA-UE, MBI | 20.37±6.54d | subacute | Severe | Yes | 10/6 | 7/9 | / | / |
|  |  | CON | 19(12/7) | 53.67±8.03 | CON |  |  | 23.65±7.24d |  |  |  | 11/8 | 10/9 |  |  |
| Chen et al. 2024 | China | Acup | 31 | / | CON+Acup | Once/d, 10d | FMA-UE | 11.50(7.00–20.50)d | subacute | / | / | / | 15/15 | / | No |
|  |  | sham | 15 | / | CON+Sham Acup |  |  | 12.50(4.30–34.80)d |  |  |  | / | 9/7 |  |  |
| Zhang et al. 2017 | China | Acup | 20(13/7) | 62.42±9.75 | Acup | 5d/wk, 12wk | FMA-UE | 0.93±1.34Y | chronic | Moderate | Yes | / | / | / | / |
|  |  | CIMT | 20(13/7) | 63.36±9.94 | CIMT |  |  | 0.85±1.62Y |  |  |  | / | / |  |  |
|  |  | CON | 20(14/6) | 60.50±10.81 | CON |  |  | 1.00±1.53Y |  |  |  | / | / |  |  |
| Ambrosini et al. 2021 | Italy | RAT | 36(25/11) | 60.9±13.7 | RAT | 3x/wk, 9wk | ARAT, BBT | 63.4±58.8d | subacute | / | Yes | 27/9 | 21/15 | / | / |
|  |  | CON | 36(25/11) | 67.8±12.2 | CON |  |  | 62.1±76.9d |  |  |  | 26/10 | 23/13 |  |  |
| Aprile et al. 2020 | Italy | RAT | 111(63/48) | 69.5±10.9 | RAT | 5d/wk, 6wk | FMA-UE, MBI, ARAT | 2W-6M | subacute | Severe | Yes | 81/30 | 63/48 | / | / |
|  |  | CON | 113(64/49) | 68.5±11.5 | CON |  |  | 2W-6M |  |  |  | 84/29 | 55/58 |  |  |
| Bhattacharjee et al. 2024 | India | RAT | 22(13/9) | 53.32±9.93 | RAT | 5d/wk, 3wk | FMA-UE | 4.14±1.21M | subacute | Moderate | Yes | 14/8 | 11/11 | / | No |
|  |  | CON | 22(12/10) | 53.23±10.51 | CON |  |  | 4.09±1.57M |  |  |  | 13/9 | 10/12 |  |  |
| Budhota et al. 2021 | Singapore | RAT | 22(11/11) | 56.32±10.37 | RAT | 3x/wk, 6wk | FMA-UE, ARAT, GS | 458(451.3)d | / | Moderate | Yes | 12/10 | 14/8 | / | No |
|  |  | CON | 22(14/8) | 54.59±10.92 | CON |  |  | 390(327.5)d |  |  |  | 10/12 | 15/7 |  |  |
| Chen et al. 2023 | China | RAT | 40(34/6) | 50.1±10.0 | RAT | 5d/wk, 4wk | FMA-UE, MBI | 50.3±40.3d | subacute | Severe | Yes | 21/19 | 22/18 | / | No |
|  |  | CON | 40(33/7) | 53.7±9.4 | CON |  |  | 58.9±35.4d |  |  |  | 20/20 | 17/23 |  |  |
| Dehem et al. 2019 | Belgium | RAT | 23(11/12) | 67.3±11.1 | RAT | 4x/wk, 9wk | FMA-UE, BBT, WMFT | / | acute | Moderate | Yes | 16/7 | 13/10 | 3/20/0 | / |
|  |  | CON | 22(10/12) | 68.6±19.1 | CON |  |  | / |  |  |  | 19/3 | 10/12 | 3/19/0 |  |
| Hsieh et al. 2018 | China | RAT | 13(8/5) | 50.35±16.65 | RAT | 5d/wk, 4wk | FMA-UE | 14.92±6.59M | chronic | Moderate | Yes | 8/5 | 8/5 | 0/13/0 | No |
|  |  | CON | 12(8/4) | 55.27±10.50 | CON |  |  | 25.33±17.46M |  |  |  | 6/6 | 5/7 | 0/12/0 |  |
| Kim et al. 2023 | Korea | RAT | 18(8/10) | 60.5±7.8 | RAT | Once/d, 30d | WMFT, FMA-UE, MBI | 29.1±6.2d | subacute | Moderate | / | 13/5 | 12/6 | 2/16/0 | / |
|  |  | CON | 18(9/9) | 61.3±8.4 | CON |  |  | 29.6±7.2d |  |  |  | 13/5 | 12/6 | 2/16/0 |  |
| Pavan et al. 2024 | Italy | RAT | 16(11/5) | 65.7±11.3 | RAT | 4d/wk, 5wk | FMA-UE, ARAT | 126.8±85.3d | subacute | Moderate | / | / | / | / | No |
|  |  | ULT | 14(6/8) | 65.7±13.3 | ULT |  |  | 122.7±73.9d |  |  |  | / | / |  |  |
| Prange et al. 2015 | Netherlands | RAT | 35(17/18) | 60.3±9.7 | RAT | 3x/wk, 6wk | FMA-UE | 7.3±3.4W | subacute | / | / | 28/7 | 10/25 | / | / |
|  |  | CON | 43(24/19) | 58±11.4 | CON |  |  | 6.8±3.1W |  |  |  | 25/8 | 17/16 |  |  |
| Rémy-Néris et al. 2021 | France | RAT | 107(67/40) | 58.08±14.05 | RAT | Twice/d, 5d/wk, 4wk | FMA-UE, ARAT | 55.67±21.6d | subacute | Moderate | / | 77/30 | 64/42 | 6/99* | Yes |
|  |  | CON | 108(73/35) | 58.53±13.27 | CON |  |  | 53.93±22.68d |  |  |  | 75/33 | 61/46 | 7/100* |  |
| Tomic et al. 2017 | Serbia | RAT | 13(12/1) | 56.5±7.4 | RAT | 5d/wk, 3wk | FMA-UE, WMFT | 35.3±9.7d | subacute | Moderate | Yes | 12/1 | 5/8 | / | No |
|  |  | CON | 13(9/4) | 58.3±5.2 | CON |  |  | 37.3±7.7d |  |  |  | 11/2 | 6/7 |  |  |
| Wongwatcharanon et al. 2025 | Thailand | RAT | 9(8/1) | 61.89±5.62 | RAT | 3x/wk, 8wk | FMA-UE | 6.34±4.08Y | chronic | Moderate | Yes | 9/0 | 3/6 | 2/7/0 | / |
|  |  | CON | 9(4/5) | 65.33±11.09 | CON |  |  | 9±5.81Y |  |  |  | 9/0 | 5/4 | 1/8/0 |  |
| Hou et al. 2019 | China | RAT | 30(20/10) | 60.36±13.46 | RAT | 5d/wk, 4wk | MBI | 4.32±2.21M | / | Moderate | / | 19/11 | 17/13 | / | / |
|  |  | CON | 25(13/12) | 59.37±12.33 | CON |  |  | 5.88±5.79M |  |  |  | 16/9 | 11/14 |  |  |
| Zhang et al. 2016 | China | RAT | 6(4/2) | 35.5±9.0 | CON+RAT | 5d/wk, 4wk | FMA-UE | 27.0±24.8M | chronic | Moderate | Yes | 4/2 | 3/3 | / | / |
|  |  | ULT | 6(5/1) | 47.0±10.0 | CON+RTP |  |  | 8.0±1.1M |  |  |  | 5/1 | 3/3 |  |  |
| Kim et al. 2015 | Korea | BF | 20(13/7) | 62.5±9.9 | BF | 3x/wk, 4wk | FMA-UE | 12.2±4.9M | chronic | Moderate | Yes | 8/12 | 9/11 | / | / |
|  |  | CON | 20(11/9) | 58.5±11.8 | CON |  |  | 18.4±13.2M |  |  |  | 11/9 | 10/10 |  |  |
| Lin et al. 2015 | China | BF | 16(12/4) | 52.63±10.49 | BF | 3d/wk, 4wk | FMA-UE, WMFT | 27.75±19.04M | chronic | Moderate | / | 9/7 | 8/8 | 4/12/0 | / |
|  |  | CON | 17(16/1) | 57.47±10.29 | CON |  |  | 21.82±21.66M |  |  |  | 6/11 | 8/9 | 0/17/0 |  |
| Seok et al. 2016 | Korea | BF | 10(7/3) | 58.7±12.37 | VBT | Once/d, 2w | GS, WMFT, FMA-UE, MBI | 38.2+14.34d | subacute | Moderate | Yes | 5/5 | 7/3 | 0/10/0 | No |
|  |  | CON | 10(6/4) | 62.0±11.80 | CON |  |  | 32.3+16.51d |  |  |  | 7/3 | 6/4 | 0/10/0 |  |
| Fu et al. 2023 | China | BCI | 30(23/7) | 55.93±11.05 | BCI | 5d/wk,4wk | FMA-UE | 77.50±33.75,175.25d | / | Severe | / | 24/6 | / | / | / |
|  |  | TOT | 31(25/6) | 59.00±14.49 | TOT |  |  | 64.00±37.00,150.00d |  |  |  | 24/7 | / |  |  |
| Ji et al. 2025 | China | BCI | 20(12/8) | 61.75 ± 10.35 | BCI+CON | 5d/wk,4wk | FMA-UE,ARAT,MBI | 49.50 ± 23.36d | subacute | Severe | / | 15/5 | 20/0 | / | No |
|  |  | RAT | 19(15/4) | 60.05 ± 14.35 | RAT+CON |  |  | 42.42 ± 24.50d |  |  |  | 12/7 | 19/0 |  |  |
| Ming et al. 2025 | China | BCI | 15(8/7) | 56.13 ± 8.63 | BCI | 5d/wk,4wk | FMA-UE,ARAT,MBI | 44.07 ± 22.63d | subacute | Severe | Yes | 10/5 | 4/11 | / | / |
|  |  | tDCS | 14(9/5) | 60.79±11.48 | tDCS |  |  | 39.36 ± 12.67d |  |  |  | 10/4 | 5/9 |  |  |
| He et al. 2025 | China | BCI | 25(20/5) | 58.6±12.8 | BCI+CON | 5d/wk,2wk | FMA-UE | 33.3 ± 18.5d | subacute | Severe | Yes | 25/0 | 11/14 | / | / |
|  |  | sham | 23(14/9) | 58.3 ± 12.8 | sham BCI+CON |  |  | 41.8 ± 19.1d |  |  |  | 23/0 | 12/11 |  |  |
| Liang et al. 2020 | China | BCI | 15(12/3) | 50.60±13.46 | BCI+CON | 5d/wk,4wk | FMA-UE,MBI,MAS | 3.00±2.98M | subacute | Severe | Yes | 8/7 | / | / | / |
|  |  | ULT | 15(9/6) | 57.94±8.84 | ULT+CON |  |  | 2.93±1.44M |  |  |  | 7/8 | / |  |  |
| Liu et al. 2023 | China | BCI | 19(15/4) | 51.26±11.06 | BCI+CON | 5d/wk,4wk | FMA-UE,MBI,MAS | 98.26±48.25d | subacute | / | Yes | 8/10 | 10/9 | / | / |
|  |  | RAT | 18(10/8) | 52.89±13.07 | RAT+CON |  |  | 90.28±52.15d |  |  |  | 5/14 | 7/11 |  |  |
| Yang et al. 2023 | China | BCI | 25(18/7) | 55.72±11.55 | BCI+CON | 5d/wk,2wk | FMA-UE,MBI | 66.28±37.44d | subacute | Moderate | Yes | / | / | / | / |
|  |  | ULT | 25(16/9) | 50.92±10.43 | ULT+CON |  |  | 83.16±46.89d |  |  |  | / | / |  |  |
| Wang et al. 2026 | China | MIT | 32(23/9) | 56.97±8.20 | MIT+CON | 5d/wk,4wk | FMA-UE,MBI | 4.41±1.58M | / | Severe | Yes | 22/10 | 13/19 | / | / |
|  |  | sham | 31(24/7 ) | 55.06±8.10 | sham MIT+CON |  |  | 4.87±2.14M |  |  |  | 23/8 | 11/20 |  |  |
| Wu et al. 2013 | China | MT | 16(11/5) | 54.77±11.66 | MT | 5d/wk,4wk | FMA-UE | 19.31±12.57M | chronic | Moderate | Yes | / | 8/8 | / | No |
|  |  | TOT | 17(12/5) | 53.59±10.21 | TOT |  |  | 21.88±15.55M |  |  |  | / | 10/7 |  |  |
| Au-Yeung et al. 2014 | China | PES | 29(17/12) | 69.7±10.6 | ES+CON | 5d/wk,4wk | ARAT,GS | 29.4±8.0h | acute | / | Yes | / | 23/6 | / | / |
|  |  | sham | 21(11/10) | 68.4±9.9 | sham ES+CON |  |  | 28.4±8.7h |  |  |  | / | 12/9 |  |  |
| Knutson et al. 2026 | United States | PES | 36(23/13) | 58.7(10.9) | FES | 22/12wk | FMA-UE,BBT,ARAT | 12.4±5.3M | chronic | Moderate | / | / | 17/19 | / | No |
|  |  | TOT | 38(29/9) | 60.6(12.5) | TOT |  |  | 13.5±5.1M |  |  |  | / | 19/19 |  |  |
| Morone et al. 2026 | Italy | RAT | 38(26/12) | 62±15 | RAT | 5d/wk,5wk | FMA-UE,BBT,MAS | 34±28d | subacute | / | Yes | 38/0 | 22/16 | / | / |
|  |  | CON | 44(26/18) | 63±12 | CON |  |  | 34±29d |  |  |  | 44/0 | 26/18 |  |  |
| Patrizio et al. 2014 | Italy | RAT | 26(15/11) | 67.7(14.2) | RAT | 5d/wk,6wk | FMA-UE | 30±7d | subacute | Moderate | Yes | 25/1 | 16/10 | / | / |
|  |  | CON | 27(16/11) | 67.7(14.2) | CON |  |  | 30±7d |  |  |  | 21/6 | 13/14 |  |  |
| Timmermans et al. 2014 | Netherlands | RAT | 11(8/3) | 61.8(6.8) | RAT | 4d/wk,8wk | FMA-UE,ARAT | 2.8±2.9Y | chronic | Mild | Yes | / | 7/4 | 5/6/0 | No |
|  |  | TOT | 11(8/3) | 56.8(6.4) | TOT |  |  | 3.7±3.0Y |  |  |  | / | 8/3 | 1/10/0 |  |
| Lee et al. 2014 | Korea | tDCS | 19(10/9) | 60.3±11.3 | tDCS | 5d/wk,3wk | FMA-UE,BBT,MAS,MBI | 17.4±9.4d | subacute | Moderate | Yes | 9/10 | 11/8 | / | No |
|  |  | VR | 20(9/11) | 60.6±14.1 | VR |  |  | 16.9±5.5d |  |  |  | 14/6 | 7/13 |  |  |
| Hou et al. 2026 | China | tDCS | 15(9/6) | 66.80±12.67 | tDCS+CON | 6d/wk,3wk | FMA-UE,WMFT | 26(12.00,90.00)d | subacute | Moderate | Yes | 15/0 | 7/8 | / | No |
|  |  | sham | 15(10/5) | 69.13±8.25 | sham tDCS+CON |  |  | 28(17.00,40.00)d |  |  |  | 15/0 | 6/9 |  |  |
| Seniów et al. 2012 | Poland | TMS | 20(12/8) | 63.5(8.9) | TMS+CON | 5d/wk,3wk | FMA-UE | 41.7±21.3)d | subacute | Moderate | Yes | 18/2 | 10/10 | / | / |
|  |  | sham | 20(14/6) | 63.4(9.2) | sham TMS+CON |  |  | 38.0±26.6d |  |  |  | 17/3 | 13/7 |  |  |
| Lin et al. 2009 | China | CIMT | 16(11/5) | 54.14(11) | CIMT | 5d/wk,3wk | FMA-UE | 16.93±11.13M | chronic | Moderate | Yes | / | 9/7 | / | / |
|  |  | CON | 16(11/5) | 57.38(12.78) | CON |  |  | 13.23±8.84M |  |  |  | / | 8/8 |  |  |
| Shimodozono et al. 2013 | Japan | RFE | 26(16/10) | 63.9±12.4 | RFE | 5d/wk,4wk | ARAT,FMA-UE | 6.4±2.1W | subacute | Moderate | Yes | 12/14 | 13/13 | / | No |
|  |  | CON | 23(10/13) | 67.0±15.0 | CON |  |  | 7.4±3.0W |  |  |  | 12/11 | 16/7 |  |  |
| Singh et al. 2013 | India | CIMT | 20(14/6) | 55.2±9.27 | CIMT | 5d/wk,2wk | WMFT,FMA-UE | 18.3+3.31d | subacute | Moderate | Yes | / | / | / | / |
|  |  | CON | 20(11/9) | 56.4±11.40 | CON |  |  | 19.60±3.85d |  |  |  | / | / |  |  |
| Wu et al. 2007 | China | CIMT | 24(16/8 ) | 53.93±11.20 | CIMT | 5d/wk,3wk | FMA-UE | 12.51±9.64M | chronic | Moderate | Yes | / | 11/13 | / | / |
|  |  | CON | 23(17/6 ) | 56.77±12.90 | CON |  |  | 11.98±11.72M |  |  |  | / | 11/12 |  |  |
| Choi et al. 2014 | Korea | VR | 10(5/5) | 64.30+10.3 | VR | 5d/wk,4wk | FMA-UE,BBT,GS,MBI | 20.20+14.1d | subacute | Moderate | / | 8/2 | 6/4 | / | No |
|  |  | CON | 10(5/5) | 64.70+11.3 | CON |  |  | 23.67±20.7d |  |  |  | 6/4 | 6/4 |  |  |
| Sun et al. 2026 | China | VR | 38(29/9) | 57.18±9.40 | VR+CON | 6d/wk,4wk | FMA-UE | 31.68±12.58d | subacute | Moderate | Yes | 38/0 | 19/19 | / | / |
|  |  | ULT | 38(30/8) | 55.66±11.73 | ULT+CON |  |  | 30.03±11.86d |  |  |  | 38/0 | 21/17 |  |  |

Notes: ULT: upper limb training; BCI: brain computer interface; RFE: repetitive facilitative exercise; PES: peripheral electrical stimulation; NMES: neuromuscular electrical stimulation; CON: conventional therapy; sham: sham stimulation; TEAS: transcutaneous electrical acupoint stimulation; TMS: transcranial magnetic stimulation; LF-rTMS: Low-Frequency Repetitive Transcranial Magnetic Stimulation; iTBS: intermittent theta-burst stimulation; RAT: robot-assisted training; BF: biofeedback; tDCS: transcranial direct current stimulation; MT: mirror therapy; CIMT: constraint-induced movement therapy; VNS: Vagus nerve stimulation; taVNS: transcutaneous auricular vagus nerve stimulation; BAT: bilateral arm training; HABIT: Hand-Arm Bimanual Intensive Training; MIT: motor imagery training; TOMP: task-oriented mental practice; TOT: task-oriented training; VR: virtual reality; Acup: acupuncture; RTP: Repetitive Task Practice; VBT: Visual Biofeedback Training; FMA-UE: Fugl-Meyer Assessment for Upper Extremity; ADL: Activities of Daily Living; ARAT: Action Research Arm Test; WMFT: Wolf Motor Function Test; BBT: Box and Block Test; GS: Grip Strength; MAS: Modified Ashworth Scale; MBI: Modified Barthel Index; R: Right; L: Left; A: Ambidextrous.

**Table S5. Risk of bias summary**

| **Study ID** | **Randomization process** | **Deviations from intended interventions** | **Missing outcome data** | **Measurement of the outcome** | **Selection of the reported result** | **Overall Bias** |
| --- | --- | --- | --- | --- | --- | --- |
| Ohnishi H., et al. 2022 | Some concerns | Some concerns | Low | High | Some concerns | High |
| Wang et al.2023a | Low | Low | Low | Low | Low | Low |
| Peng et al. 2015 | Some concerns | Some concerns | Low | Some concerns | Some concerns | Some concerns |
| Xia et al. 2021 | Some concerns | Low | Low | Low | Low | Some concerns |
| Sharma et al. 2020 | Low | Low | Low | Low | Some concerns | Some concerns |
| Wang et al. 2023b | Low | Low | Low | High | Low | High |
| Long et al. 2018 | Some concerns | Low | Low | Low | Some concerns | Some concerns |
| Dou et al. 2019 | Some concerns | Low | Low | High | Some concerns | High |
| Sun et al. 2024 | Some concerns | Low | Low | Low | Some concerns | Some concerns |
| Sun et al. 2022 | Some concerns | Low | Low | High | Some concerns | High |
| Yang et al. 2020 | Some concerns | Low | Low | High | Some concerns | High |
| Zhao et al. 2015 | Some concerns | Some concerns | Low | Low | Some concerns | Some concerns |
| Allman et al. 2016 | Low | Low | Low | Low | Low | Low |
| Gerloff et al. 2024 | Low | Low | Low | Low | Low | Low |
| Vimolratana et al. 2024 | Low | Some concerns | Low | Low | Low | Some concerns |
| Wei et al. 2022 | Low | Low | Low | Low | Low | Low |
| Yin et al. 2015 | Some concerns | Low | Low | Low | Some concerns | Some concerns |
| Gurbuz et al. 2016 | Some concerns | Low | Low | Low | Some concerns | Some concerns |
| Hsu et al. 2022 | Low | Low | Low | Low | High | High |
| Kaviraja et al. 2021 | Some concerns | Some concerns | Low | High | Some concerns | High |
| Kim et al. 2016 | Low | Low | Low | Low | Some concerns | Some concerns |
| Lim et al. 2016 | Some concerns | Low | Low | High | Some concerns | High |
| Zhuang et al. 2021 | Low | Low | Low | Low | High | High |
| Du et al. 2016 | Some concerns | Low | Low | High | Some concerns | High |
| Dawson et al. 2021 | Low | Low | Low | Low | Low | Low |
| Kimberley et al. 2018 | Low | Low | Low | Low | Low | Low |
| Zhang et al. 2020 | Some concerns | Low | Low | Low | High | High |
| Zhang et al. 2023 | Some concerns | Low | Low | Low | Some concerns | Some concerns |
| Meng et al. 2018 | Low | Some concerns | Low | Low | Low | Some concerns |
| Thrane et al. 2015 | Low | Low | Low | Low | Some concerns | Some concerns |
| Zhang et al. 2025 | Low | Some concerns | Low | Low | Low | Some concerns |
| Zhang et al. 2024 | Some concerns | Low | Low | Low | Some concerns | Some concerns |
| Park 2022 | Low | High | Low | Low | Some concerns | High |
| Jiang et al. 2022 | Some concerns | Low | Low | High | Some concerns | High |
| Wang et al. 2020 | Some concerns | Low | Low | High | Some concerns | High |
| Zhang et al. 2021 | Some concerns | Low | Low | High | Some concerns | High |
| Adie et al. 2017 | Low | Low | Low | Low | Low | Low |
| Ali et al. 2024 | Low | Low | Low | Low | Low | Low |
| Anwar et al. 2022 | Some concerns | Some concerns | Low | Low | Some concerns | Some concerns |
| Kuo et al. 2023 | Low | Low | Low | Low | Low | Low |
| Oh et al. 2019 | Low | Some concerns | Low | Low | Some concerns | Some concerns |
| Schuster-Amft et al. 2018 | Low | Low | Low | Low | Low | Low |
| Shin et al. 2016 | Low | Low | Low | Low | Some concerns | Some concerns |
| Han et al. 2016 | Some concerns | Some concerns | Low | Low | Some concerns | Some concerns |
| Xiao et al. 2019 | Some concerns | Low | Low | Low | Some concerns | Some concerns |
| Chen et al. 2024 | Low | High | Low | Low | Low | High |
| Zhang et al. 2017 | Some concerns | Low | Low | Low | Some concerns | Some concerns |
| Ambrosini et al. 2021 | Low | Low | Low | Low | Some concerns | Some concerns |
| Aprile et al. 2020 | Some concerns | Low | High | Low | Low | High |
| Bhattacharjee et al. 2024 | Low | Low | Low | Low | Some concerns | Some concerns |
| Budhota et al. 2021 | Some concerns | Low | Low | Low | Low | Some concerns |
| Chen et al. 2023 | Some concerns | Low | Low | Low | Low | Some concerns |
| Dehem et al. 2019 | Low | Low | Low | Low | Low | Low |
| Hsieh et al. 2018 | Some concerns | High | Low | Low | Some concerns | High |
| Kim et al. 2023 | Some concerns | Low | Low | Low | Some concerns | Some concerns |
| Pavan et al. 2024 | Some concerns | Low | Low | Low | Some concerns | Some concerns |
| Prange et al. 2015 | Low | Some concerns | Low | Low | Some concerns | Some concerns |
| Rémy-Néris et al. 2021 | Low | Low | Low | Low | Low | Low |
| Tomic et al. 2017 | Some concerns | Low | Low | Low | High | High |
| Wongwatcharanon et al. 2025 | Some concerns | Low | Low | High | Low | High |
| Hou et al. 2019 | Some concerns | Low | Low | High | Some concerns | High |
| Zhang et al. 2016 | Some concerns | Low | Low | Low | Some concerns | Some concerns |
| Kim et al. 2015 | Low | Low | Low | Low | Some concerns | Some concerns |
| Lin et al. 2015 | Some concerns | Low | Low | Low | Low | Some concerns |
| Seok et al. 2016 | Some concerns | Some concerns | Low | High | Some concerns | High |
| de Sire et al. 2025 | Some concerns | Low | Low | Low | Some concerns | Some concerns |
| Fu et al. 2023 | Low | Low | Low | Low | Low | Low |
| Ji et al. 2025 | Low | Low | Low | Low | Low | Low |
| Ming et al. 2025 | Low | High | Low | Low | Low | High |
| He et al. 2025 | Low | Low | Low | Low | Low | Low |
| Liang et al. 2020 | Low | Some concerns | Low | Low | Some concerns | Some concerns |
| Liu et al. 2023 | Low | Some concerns | Low | Low | Some concerns | Some concerns |
| Yang et al. 2023 | Low | Low | Low | Low | Some concerns | Some concerns |
| Wang et al. 2026 | Low | High | Low | Low | Some concerns | High |
| Wu et al. 2013 | Low | Low | Low | Low | Low | Low |
| Au-Yeung et al. 2014 | Low | Low | Low | High | Some concerns | High |
| Knutson et al. 2026 | Low | Low | Low | Low | Low | Low |
| Morone et al. 2026 | Low | Low | Low | Low | Low | Low |
| Patrizio et al. 2014 | Low | Low | Low | Low | Some concerns | Some concerns |
| Timmermans et al. 2014 | Low | Some concerns | Low | Low | Low | Some concerns |
| Lee et al. 2014 | Low | Some concerns | Low | Low | Some concerns | Some concerns |
| Hou et al. 2026 | Low | Low | Low | Low | Low | Low |
| Seniów et al. 2012 | Low | Low | Low | Low | Some concerns | Some concerns |
| Lin et al. 2009 | Some concerns | Low | Low | Low | Some concerns | Some concerns |
| Shimodozono et al. 2013 | Low | Some concerns | Low | Low | Some concerns | Some concerns |
| Singh et al. 2013 | Some concerns | High | Low | High | Some concerns | High |
| Wu et al. 2007 | Low | Low | Low | Low | Some concerns | Some concerns |
| Choi et al. 2014 | Low | Some concerns | Low | High | Some concerns | High |
| Sun et al. 2026 | Low | Low | Low | Low | Some concerns | Some concerns |

**Table S6. The PSRF value and Node-splitting test result of improvement in FMA-UE**

**(a)The PSRF value of improvement in FMA-UE**

| Parameter | PSRF |
| --- | --- |
| d.Acup.sham | 1.00 |
| d.BF.TMS | 1.00 |
| d.CON.Acup | 1.00 |
| d.CON.BF | 1.00 |
| d.CON.CIMT | 1.00 |
| d.CON.MIT | 1.00 |
| d.CON.MT | 1.00 |
| d.CON.PES | 1.00 |
| d.CON.RAT | 1.00 |
| d.CON.RFE | 1.00 |
| d.CON.ULT | 1.00 |
| d.CON.VR | 1.00 |
| d.PES.TOT | 1.00 |
| d.RAT.BCI | 1.00 |
| d.ULT.VNS | 1.00 |
| d.VR.tDCS | 1.00 |
| sd.d | 1.00 |

**(b)Node-splitting test result of improvement in FMA-UE**

| Name | Direct Effect | Indirect Effect | Overall | *P-Value* |
| --- | --- | --- | --- | --- |
| Acup, CIMT | 0.26 (-8.44, 9.02) | 5.17 (-2.72, 12.90) | 2.93 (-3.85, 9.66) | 0.38 |
| Acup, CON | -2.50 (-10.99, 6.17) | -7.74 (-14.70, -0.62) | -5.34 (-11.71, 1.10) | 0.34 |
| Acup, sham | -7.50 (-17.42, 2.56) | -6.26 (-14.77, 2.25) | -6.57 (-13.20, 0.08) | 0.85 |
| BCI, RAT | -8.56 (-14.31, -2.58) | -3.08 (-8.05, 1.91) | -5.30 (-9.18, -1.45) | 0.16 |
| BCI, TOT | -5.51 (-13.79, 2.84) | -5.99 (-11.05, -0.83) | -5.84 (-10.21, -1.47) | 0.92 |
| BCI, ULT | -9.09 (-20.35, 2.01) | -4.73 (-9.78, 0.28) | -5.37 (-10.10, -0.73) | 0.49 |
| BCI, sham | -5.47 (-10.85, 0.08) | -12.56 (-17.14, -7.94) | -9.62 (-13.23, -5.94) | 0.05 |
| BCI, tDCS | -6.92 (-14.49, 0.59) | -5.21 (-11.32, 0.98) | -5.85 (-10.60, -0.96) | 0.72 |
| BF, CON | -7.65 (-14.56, -0.62) | -7.67 (-16.88, 1.76) | -7.51 (-12.97, -2.01) | 1.00 |
| BF, TMS | -4.33 (-12.48, 4.08) | -4.41 (-12.66, 3.92) | -4.25 (-9.93, 1.62) | 0.99 |
| CIMT, CON | -6.93 (-10.97, -2.78) | -13.66 (-21.75, -5.29) | -8.24 (-11.91, -4.39) | 0.15 |
| CIMT, MT | -10.86 (-19.62, -1.94) | -1.46 (-7.42, 4.78) | -4.46 (-9.52, 0.60) | 0.08 |
| CON, MIT | 3.85 (-4.28, 11.68) | 4.17 (-1.44, 9.83) | 4.04 (-0.44, 8.56) | 0.93 |
| CON, MT | 1.98 (-11.65, 15.38) | 3.98 (-0.48, 8.50) | 3.78 (-0.60, 8.03) | 0.79 |
| CON, PES | 3.04 (-5.23, 11.26) | 7.41 (2.32, 12.47) | 6.15 (1.88, 10.44) | 0.37 |
| CON, RAT | 3.47 (0.83, 6.07) | 1.79 (-2.91, 6.51) | 3.02 (0.77, 5.38) | 0.55 |
| CON, RFE | 5.26 (0.78, 9.74) | -0.11 (-13.89, 13.44) | 5.12 (0.89, 9.16) | 0.46 |
| CON, ULT | 7.85 (-0.28, 16.29) | 1.55 (-2.83, 5.85) | 2.97 (-0.93, 6.76) | 0.18 |
| CON, VR | 5.60 (1.61, 9.62) | 6.56 (-0.14, 13.20) | 5.86 (2.35, 9.37) | 0.81 |
| MIT, MT | 0.06 (-13.22, 13.71) | -0.39 (-5.97, 5.49) | -0.28 (-5.59, 4.96) | 0.96 |
| MIT, TOT | -5.15 (-15.00, 4.63) | -0.32 (-6.01, 5.35) | -1.55 (-6.50, 3.35) | 0.40 |
| MIT, sham | -4.28 (-10.20, 1.52) | -6.49 (-12.81, -0.26) | -5.35 (-9.69, -1.05) | 0.61 |
| MT, TOT | -2.19 (-8.21, 3.89) | -0.31 (-6.08, 5.73) | -1.26 (-5.55, 3.01) | 0.66 |
| MT, ULT | -10.01 (-24.38, 4.93) | 0.41 (-5.06, 5.79) | -0.83 (-5.98, 4.20) | 0.19 |
| MT, sham | -5.98 (-12.79, 0.71) | -4.44 (-9.83, 0.99) | -5.09 (-9.26, -0.85) | 0.74 |
| PES, RFE | 0.45 (-7.84, 9.08) | -1.29 (-7.94, 5.36) | -1.01 (-6.64, 4.36) | 0.73 |
| PES, TOT | -3.70 (-11.33, 4.01) | -3.59 (-9.37, 1.98) | -3.63 (-8.21, 0.80) | 0.98 |
| PES, sham | -8.44 (-13.09, -3.88) | -5.55 (-12.09, 0.89) | -7.45 (-11.20, -3.65) | 0.48 |
| RAT, RFE | -3.29 (-16.54, 9.83) | 2.71 (-2.21, 7.46) | 2.11 (-2.48, 6.52) | 0.39 |
| RAT, TMS | -0.01 (-7.55, 7.45) | 0.30 (-4.26, 4.68) | 0.32 (-3.70, 4.10) | 0.96 |
| RAT, TOT | 1.51 (-4.18, 6.97) | -2.04 (-6.85, 2.75) | -0.48 (-4.18, 3.08) | 0.34 |
| RAT, ULT | -3.90 (-11.44, 4.13) | 1.17 (-3.47, 5.60) | -0.05 (-4.10, 3.72) | 0.26 |
| TMS, ULT | 0.14 (-7.64, 7.85) | -0.66 (-6.00, 4.54) | -0.33 (-4.81, 3.97) | 0.86 |
| TMS, sham | -4.78 (-8.34, -1.17) | -4.13 (-10.19, 2.11) | -4.61 (-7.60, -1.47) | 0.86 |
| TOT, VR | 3.96 (-6.04, 13.93) | 3.03 (-2.04, 8.34) | 3.35 (-1.26, 7.90) | 0.88 |
| ULT, VNS | -7.46 (-15.22, 0.46) | -0.27 (-6.89, 6.28) | -3.31 (-8.30, 1.94) | 0.17 |
| ULT, VR | 4.34 (-3.75, 12.58) | 2.29 (-3.20, 7.66) | 2.90 (-1.59, 7.47) | 0.69 |
| VNS, sham | -2.61 (-7.53, 2.11) | 4.69 (-4.39, 13.56) | -0.97 (-5.32, 3.31) | 0.15 |
| VR, tDCS | 0.82 (-15.30, 16.54) | -3.92 (-10.01, 2.10) | -3.40 (-8.93, 2.50) | 0.59 |
| sham, tDCS | 3.84 (-1.11, 8.84) | 3.52 (-4.15, 11.39) | 3.78 (-0.29, 7.90) | 0.94 |

**Note:** ULT: upper limb training; PES: peripheral electrical stimulation; TMS: transcranial magnetic stimulation; RAT: robot-assisted training; BF: biofeedback; tDCS: transcranial direct current stimulation; MT: mirror therapy; VNS: Vagus nerve stimulation; MIT: motor imagery training; VR: virtual reality; Acup: acupuncture; CON: conventional care or sham stimulation.

**Table S7. The PSRF value and Node-splitting test result of improvement in ADL**

**(a)The PSRF value of improvement in ADL**

| Parameter | PSRF |
| --- | --- |
| d.BCI.RAT | 1.00 |
| d.BCI.ULT | 1.00 |
| d.BCI.sham | 1.00 |
| d.BCI.tDCS | 1.00 |
| d.CON.BF | 1.00 |
| d.RAT.CON | 1.00 |
| d.RAT.TMS | 1.00 |
| d.sham.MIT | 1.00 |
| d.sham.MT | 1.00 |
| d.sham.PES | 1.00 |
| d.tDCS.VR | 1.00 |
| sd.d | 1.00 |

**(b)Node-splitting test result of improvement in ADL**

| Name | Direct Effect | Indirect Effect | Overall | *P-Value* |
| --- | --- | --- | --- | --- |
| BCI, RAT | -4.70 (-11.03, 0.36) | -8.13 (-16.78, -0.48) | -5.78 (-10.99, -1.66) | 0.46 |
| BCI, ULT | -15.52 (-27.95, -3.20) | -6.11 (-13.60, 0.27) | -8.15 (-15.32, -2.52) | 0.19 |
| BCI, sham | -12.29 (-18.02, -4.59) | -12.12 (-21.44, -6.61) | -12.11 (-17.45, -7.60) | 0.97 |
| BCI, tDCS | -9.20 (-15.99, -2.49) | -6.54 (-16.90, 3.63) | -8.62 (-13.92, -3.14) | 0.64 |
| CON, RAT | 6.39 (1.82, 10.28) | 5.16 (-14.94, 24.67) | 6.36 (2.06, 10.11) | 0.91 |
| CON, VR | 2.11 (-7.89, 11.96) | 2.80 (-14.83, 20.88) | 2.16 (-6.30, 10.62) | 0.94 |
| MIT, MT | -5.66 (-20.70, 9.80) | -5.88 (-16.94, 5.34) | -5.89 (-14.74, 2.96) | 0.98 |
| MIT, sham | -12.26 (-18.05, -6.27) | -12.03 (-30.20, 6.00) | -12.28 (-17.60, -6.75) | 0.98 |
| MT, sham | -6.42 (-16.01, 3.12) | -6.39 (-22.70, 9.62) | -6.38 (-14.40, 1.78) | 1.00 |
| RAT, TMS | 0.62 (-6.18, 7.38) | -4.79 (-11.44, 2.13) | -1.90 (-7.03, 3.09) | 0.22 |
| RAT, ULT | -3.64 (-12.40, 4.97) | -1.30 (-9.97, 5.91) | -2.31 (-8.43, 3.00) | 0.68 |
| TMS, ULT | 1.93 (-4.39, 8.44) | -5.13 (-13.57, 3.12) | -0.40 (-6.22, 4.65) | 0.15 |
| TMS, sham | -4.43 (-10.40, 1.91) | -4.49 (-13.50, 6.13) | -4.44 (-9.17, 0.70) | 0.99 |
| VR, tDCS | 0.90 (-13.89, 16.53) | 1.45 (-10.90, 14.94) | 1.43 (-8.05, 11.21) | 0.95 |
| sham, tDCS | 6.38 (-3.97, 16.86) | 2.38 (-5.50, 10.71) | 3.54 (-2.31, 10.30) | 0.51 |

**Note:** ULT: upper limb training; PES: peripheral electrical stimulation; TMS: transcranial magnetic stimulation; RAT: robot-assisted training; BF: biofeedback; tDCS: transcranial direct current stimulation; MT: mirror therapy; VNS: Vagus nerve stimulation; MIT: motor imagery training; VR: virtual reality; CON: conventional care or sham stimulation.

**Table S8. The PSRF value and Node-splitting test result of improvement in ARAT**

**(a)The PSRF value of improvement in ARAT**

| Parameter | PSRF |
| --- | --- |
| d.BCI.tDCS | 1.00 |
| d.CON.RFE | 1.00 |
| d.CON.ULT | 1.00 |
| d.MT.CIMT | 1.00 |
| d.MT.MIT | 1.00 |
| d.PES.sham | 1.00 |
| d.RAT.BCI | 1.00 |
| d.RAT.CON | 1.00 |
| d.RAT.TOT | 1.00 |
| d.TOT.MT | 1.00 |
| d.TOT.PES | 1.00 |
| d.TOT.VR | 1.00 |
| sd.d | 1.00 |

**(b)Node-splitting test result of improvement in ARAT**

| Name | Direct Effect | Indirect Effect | Overall | *P-Value* |
| --- | --- | --- | --- | --- |
| BCI, RAT | -6.93 (-16.48, 2.28) | -8.32 (-26.14, 8.82) | -7.11 (-14.96, 0.42) | 0.87 |
| BCI, tDCS | -2.28 (-11.38, 7.07) | -1.04 (-18.93, 17.58) | -2.07 (-9.24, 5.55) | 0.89 |
| CON, RAT | 3.10 (-2.75, 9.58) | -3.47 (-20.37, 13.77) | 2.44 (-3.11, 8.35) | 0.43 |
| CON, ULT | 2.33 (-6.31, 10.80) | 8.67 (-7.71, 24.84) | 3.34 (-3.48, 11.35) | 0.43 |
| PES, TOT | -4.13 (-13.32, 4.78) | -3.03 (-21.20, 15.46) | -3.91 (-10.83, 3.79) | 0.89 |
| PES, sham | -3.22 (-10.51, 5.66) | -4.59 (-23.30, 14.80) | -3.56 (-9.48, 3.91) | 0.87 |
| RAT, TOT | 1.10 (-4.62, 6.10) | -2.89 (-16.26, 9.55) | 0.55 (-4.49, 4.73) | 0.50 |
| TOT, VR | 2.97 (-7.70, 13.94) | -3.44 (-18.60, 11.82) | 0.36 (-7.61, 9.20) | 0.44 |
| ULT, VR | -1.65 (-10.86, 7.81) | 4.88 (-10.81, 20.67) | -0.06 (-7.74, 7.61) | 0.43 |
| sham, tDCS | 4.48 (-3.78, 12.49) | 3.27 (-15.39, 21.74) | 4.16 (-2.86, 11.14) | 0.89 |

**Note:** ULT: upper limb training; PES: peripheral electrical stimulation; RAT: robot-assisted training; tDCS: transcranial direct current stimulation; MT: mirror therapy; MIT: motor imagery training; VR: virtual reality; CON: conventional care or sham stimulation.

**Table S9. The PSRF value of improvement in BBT**

**(a)The PSRF value of improvement in BBT**

| Parameter | PSRF |
| --- | --- |
| d.CON.MT | 1.00 |
| d.CON.RAT | 1.00 |
| d.CON.VR | 1.00 |
| d.MT.TOT | 1.00 |
| d.RAT.ULT | 1.00 |
| d.TOT.PES | 1.00 |
| d.ULT.VNS | 1.00 |
| d.VNS.sham | 1.00 |
| d.VR.tDCS | 1.00 |
| sd.d | 1.00 |

**Note:** ULT: upper limb training; RAT: robot-assisted training; MT: mirror therapy; VNS: Vagus nerve stimulation; VR: virtual reality; CON: conventional care or sham stimulation.

**Table S10. Summary table for credibility assessment using confidence in network meta-analysis (CINeMA) for the following outcomes.**

| Comparison | Number of studies | Within-study bias | Reporting bias | Indirectness | Imprecision | Heterogeneity | Incoherence | Confidence rating |
| --- | --- | --- | --- | --- | --- | --- | --- | --- |
| **FMA-UE** |  |  |  |  |  |  |  |  |
| Acup:CIMT | 1 | Some concerns | Low risk | No concerns | Some concerns | Some concerns | No concerns | Very low |
| Acup:CON | 1 | Some concerns | Low risk | No concerns | Some concerns | No concerns | No concerns | Low |
| Acup:sham | 1 | Major concerns | Low risk | No concerns | No concerns | Some concerns | No concerns | Very low |
| BCI:RAT | 2 | Some concerns | Low risk | No concerns | No concerns | Some concerns | No concerns | Low |
| BCI:TOT | 1 | No concerns | Low risk | No concerns | No concerns | Some concerns | No concerns | Moderate |
| BCI:ULT | 1 | Some concerns | Low risk | No concerns | No concerns | Some concerns | No concerns | Low |
| BCI:sham | 2 | Some concerns | Low risk | No concerns | No concerns | No concerns | Some concerns | Low |
| BCI:tDCS | 1 | Major concerns | Low risk | No concerns | No concerns | Some concerns | No concerns | Very low |
| BF:CON | 3 | Some concerns | Low risk | No concerns | No concerns | Some concerns | No concerns | Low |
| BF:TMS | 1 | Major concerns | Low risk | No concerns | Some concerns | No concerns | No concerns | Very low |
| CIMT:CON | 5 | Some concerns | Low risk | No concerns | No concerns | No concerns | Some concerns | Low |
| CIMT:MT | 1 | Major concerns | Low risk | No concerns | Some concerns | No concerns | No concerns | Very low |
| CON:MIT | 1 | Major concerns | Low risk | No concerns | Some concerns | No concerns | No concerns | Very low |
| CON:MT | 1 | Major concerns | Low risk | No concerns | Some concerns | No concerns | No concerns | Very low |
| CON:PES | 1 | Major concerns | Low risk | No concerns | No concerns | Some concerns | No concerns | Very low |
| CON:RAT | 13 | Some concerns | Low risk | No concerns | No concerns | Some concerns | No concerns | Low |
| CON:RFE | 3 | Some concerns | Low risk | No concerns | No concerns | Some concerns | No concerns | Low |
| CON:ULT | 1 | Some concerns | Low risk | No concerns | Some concerns | No concerns | No concerns | Low |
| CON:VR | 6 | Some concerns | Low risk | No concerns | No concerns | Some concerns | No concerns | Low |
| MIT:MT | 1 | Major concerns | Low risk | No concerns | Some concerns | Some concerns | No concerns | Very low |
| MIT:TOT | 1 | Major concerns | Low risk | No concerns | Some concerns | Some concerns | No concerns | Very low |
| MIT:sham | 2 | Major concerns | Low risk | No concerns | No concerns | Some concerns | No concerns | Very low |
| MT:TOT | 2 | Some concerns | Low risk | No concerns | Some concerns | Some concerns | No concerns | Very low |
| MT:ULT | 1 | Some concerns | Low risk | No concerns | Some concerns | Some concerns | No concerns | Very low |
| MT:sham | 2 | Major concerns | Low risk | No concerns | No concerns | Some concerns | No concerns | Very low |
| PES:RFE | 1 | Major concerns | Low risk | No concerns | Some concerns | Some concerns | No concerns | Very low |
| PES:TOT | 1 | No concerns | Low risk | No concerns | Some concerns | No concerns | No concerns | Moderate |
| PES:sham | 3 | Some concerns | Low risk | No concerns | No concerns | Some concerns | No concerns | Low |
| RAT:RFE | 1 | Some concerns | Low risk | No concerns | Some concerns | Some concerns | No concerns | Very low |
| RAT:TMS | 1 | Major concerns | Low risk | No concerns | No concerns | Major concerns | No concerns | Very low |
| RAT:TOT | 2 | Some concerns | Low risk | No concerns | No concerns | Major concerns | No concerns | Very low |
| RAT:ULT | 1 | Some concerns | Low risk | No concerns | No concerns | Major concerns | No concerns | Very low |
| TMS:ULT | 1 | Major concerns | Low risk | No concerns | No concerns | Major concerns | No concerns | Very low |
| TMS:sham | 6 | Some concerns | Low risk | No concerns | No concerns | Some concerns | No concerns | Low |
| TOT:VR | 1 | Some concerns | Low risk | No concerns | Some concerns | No concerns | No concerns | Low |
| ULT:VNS | 1 | Some concerns | Low risk | No concerns | Some concerns | Some concerns | No concerns | Very low |
| ULT:VR | 1 | Some concerns | Low risk | No concerns | Some concerns | Some concerns | No concerns | Very low |
| VNS:sham | 3 | No concerns | Low risk | No concerns | No concerns | Major concerns | No concerns | Very low |
| VR:tDCS | 1 | Some concerns | Low risk | No concerns | Some concerns | Some concerns | No concerns | Very low |
| sham:tDCS | 6 | No concerns | Low risk | No concerns | Some concerns | No concerns | No concerns | Moderate |
| **ADL** |  |  |  |  |  |  |  |  |
| BCI:RAT | 2 | No concerns | Low risk | No concerns | No concerns | Some concerns | No concerns | Moderate |
| BCI:ULT | 1 | Some concerns | Low risk | No concerns | No concerns | No concerns | No concerns | Moderate |
| BCI:sham | 1 | Some concerns | Low risk | No concerns | No concerns | No concerns | No concerns | Moderate |
| BCI:tDCS | 1 | Major concerns | Low risk | No concerns | No concerns | No concerns | No concerns | Low |
| BF:CON | 1 | Major concerns | Low risk | No concerns | Major concerns | No concerns | Major concerns | Very low |
| CON:RAT | 4 | Some concerns | Low risk | No concerns | No concerns | Some concerns | No concerns | Low |
| CON:VR | 2 | Some concerns | Low risk | No concerns | Some concerns | No concerns | No concerns | Low |
| MIT:MT | 1 | Major concerns | Low risk | No concerns | Some concerns | No concerns | No concerns | Very low |
| MIT:sham | 2 | Major concerns | Low risk | No concerns | No concerns | No concerns | No concerns | Low |
| MT:sham | 1 | Major concerns | Low risk | No concerns | Some concerns | No concerns | No concerns | Very low |
| PES:sham | 2 | Some concerns | Low risk | No concerns | No concerns | No concerns | Major concerns | Very low |
| RAT:TMS | 1 | Major concerns | Low risk | No concerns | No concerns | No concerns | No concerns | Low |
| RAT:ULT | 1 | Some concerns | Low risk | No concerns | No concerns | No concerns | No concerns | Moderate |
| TMS:ULT | 1 | Major concerns | Low risk | No concerns | No concerns | No concerns | No concerns | Low |
| TMS:sham | 2 | Some concerns | Low risk | No concerns | No concerns | Some concerns | No concerns | Low |
| VR:tDCS | 1 | Some concerns | Low risk | No concerns | Some concerns | No concerns | No concerns | Low |
| sham:tDCS | 1 | Some concerns | Low risk | No concerns | No concerns | Some concerns | No concerns | Low |
| **ARAT** |  |  |  |  |  |  |  |  |
| BCI:RAT | 1 | No concerns | Low risk | No concerns | No concerns | Some concerns | No concerns | Moderate |
| BCI:tDCS | 1 | Major concerns | Low risk | No concerns | Some concerns | Some concerns | No concerns | Very low |
| CIMT:MT | 1 | Major concerns | Low risk | No concerns | Some concerns | Some concerns | Major concerns | Very low |
| CON:RAT | 3 | Major concerns | Low risk | No concerns | Some concerns | Some concerns | No concerns | Very low |
| CON:RFE | 1 | Some concerns | Low risk | No concerns | No concerns | Some concerns | Major concerns | Very low |
| CON:ULT | 1 | Some concerns | Low risk | No concerns | Some concerns | No concerns | No concerns | Low |
| MIT:MT | 1 | Major concerns | Low risk | No concerns | Major concerns | No concerns | Major concerns | Very low |
| MT:TOT | 1 | Major concerns | Low risk | No concerns | Some concerns | Some concerns | Major concerns | Very low |
| PES:TOT | 1 | No concerns | Low risk | No concerns | Some concerns | No concerns | No concerns | Moderate |
| PES:sham | 2 | Some concerns | Low risk | No concerns | Some concerns | No concerns | No concerns | Low |
| RAT:TOT | 3 | Some concerns | Low risk | No concerns | No concerns | Major concerns | No concerns | Very low |
| TOT:VR | 1 | No concerns | Low risk | No concerns | Major concerns | No concerns | No concerns | Very low |
| ULT:VR | 1 | No concerns | Low risk | No concerns | Major concerns | No concerns | No concerns | Very low |
| sham:tDCS | 3 | Some concerns | Low risk | No concerns | Some concerns | No concerns | No concerns | Low |
| **BBT** |  |  |  |  |  |  |  |  |
| CON:MT | 1 | Major concerns | Low risk | No concerns | Major concerns | No concerns | No concerns | Very low |
| CON:RAT | 3 | No concerns | Low risk | No concerns | Some concerns | No concerns | No concerns | Moderate |
| CON:VR | 4 | No concerns | Low risk | No concerns | No concerns | No concerns | No concerns | High |
| MT:TOT | 1 | Some concerns | Low risk | No concerns | No concerns | No concerns | No concerns | Moderate |
| MT:ULT | 1 | Major concerns | Low risk | No concerns | Major concerns | No concerns | No concerns | Very low |
| PES:TOT | 1 | No concerns | Low risk | No concerns | No concerns | No concerns | No concerns | High |
| RAT:ULT | 1 | Some concerns | Low risk | No concerns | No concerns | No concerns | No concerns | Moderate |
| ULT:VNS | 1 | Some concerns | Low risk | No concerns | No concerns | No concerns | No concerns | Moderate |
| VNS:sham | 1 | No concerns | Low risk | No concerns | Major concerns | No concerns | No concerns | Low |
| VR:tDCS | 1 | Some concerns | Low risk | No concerns | Some concerns | Some concerns | No concerns | Very low |
